# Baclofen, a GABA_B_R Agonist, Ameliorates Immune-Complex Mediated Acute Lung Injury by Modulating Pro-Inflammatory Mediators

**DOI:** 10.1371/journal.pone.0121637

**Published:** 2015-04-07

**Authors:** Shunying Jin, Michael L. Merchant, Jeffrey D. Ritzenthaler, Kenneth R. McLeish, Eleanor D. Lederer, Edilson Torres-Gonzalez, Mostafa Fraig, Michelle T. Barati, Alex B. Lentsch, Jesse Roman, Jon B. Klein, Madhavi J. Rane

**Affiliations:** 1 Department of Medicine, University of Louisville, Louisville, Kentucky, United States of America; 2 Department of Biochemistry and Molecular Biology, University of Louisville, Louisville, Kentucky, United States of America; 3 Robley Rex VA Medical Center, Zorn Avenue, Louisville, Kentucky, United States of America; 4 Department of Physiology, University of Louisville, Louisville, Kentucky, United States of America; 5 Department of Pathology, University of Louisville, Louisville, Kentucky, United States of America; 6 Department of Surgery, University of Cincinnati, Cincinnati, OH, United States of America; The Hospital for Sick Children and The University of Toronto, CANADA

## Abstract

Immune-complexes play an important role in the inflammatory diseases of the lung. Neutrophil activation mediates immune-complex (IC) deposition-induced acute lung injury (ALI). Components of gamma amino butyric acid (GABA) signaling, including GABA B receptor 2 (GABA_B_R2), GAD65/67 and the GABA transporter, are present in the lungs and in the neutrophils. However, the role of pulmonary GABA_B_R activation in the context of neutrophil-mediated ALI has not been determined. Thus, the objective of the current study was to determine whether administration of a GABA_B_R agonist, baclofen would ameliorate or exacerbate ALI. We hypothesized that baclofen would regulate IC-induced ALI by preserving pulmonary GABA_B_R expression. Rats were subjected to sham injury or IC-induced ALI and two hours later rats were treated intratracheally with saline or 1 mg/kg baclofen for 2 additional hours and sacrificed. ALI was assessed by vascular leakage, histology, TUNEL, and lung caspase-3 cleavage. ALI increased total protein, tumor necrosis factor α (TNF-α and interleukin-1 receptor associated protein (IL-1R AcP), in the bronchoalveolar lavage fluid (BALF). Moreover, ALI decreased lung GABA_B_R2 expression, increased phospho-p38 MAPK, promoted IκB degradation and increased neutrophil influx in the lung. Administration of baclofen, after initiation of ALI, restored GABA_B_R expression, which was inhibited in the presence of a GABA_B_R antagonist, CGP52432. Baclofen administration activated pulmonary phospho-ERK and inhibited p38 MAPK phosphorylation and IκB degradation. Additionally, baclofen significantly inhibited pro-inflammatory TNF-α and IL-1βAcP release and promoted BAL neutrophil apoptosis. Protective effects of baclofen treatment on ALI were possibly mediated by inhibition of TNF-α- and IL-1β-mediated inflammatory signaling. Interestingly, GABA_B_R2 expression was regulated in the type II pneumocytes in lung tissue sections from lung injured patients, further suggesting a physiological role for GABA_B_R2 in the repair process of lung damage. GABA_B_R2 agonists may play a potential therapeutic role in ALI.

## Introduction

Acute lung injury (ALI) and acute respiratory distress syndrome (ARDS) are a major cause of morbidity and mortality in critically ill patients [1]. The incidence of ALI is 20,000 per year in the US and is associated with high health care costs. Pathogenesis of ALI/ARDS is associated with damage to vascular endothelium and the alveolar epithelium. No effective therapies exist for treatment of ALI. Studies show that the long-term quality of life is adversely affected in patients who survive ALI due to chronic lung disease [2,3]. Therefore, there is an urgent need to identify new molecular targets to allow for generation of new therapies to treat ALI/ARDS and possibly improve the quality of life of these patients.

Different animal models of experimental lung injury have been used to study the mechanisms regulating ALI [4]. In the current study we utilized the immune-complex deposition (IC) model of ALI because it is a very reproducible model of neutrophil-mediated lung injury. Bronchoalveolar lavage fluid (BALF) obtained 4 h after IC deposition contained mainly neutrophils (>95%), as previously described by Dr. Peter Ward’s group [5]. This acute inflammatory lung injury model generated by intrapulmonary deposition of IC is characterized by an extensive neutrophil accumulation, interstitial and intra-alveolar edema, and hemorrhage [5–9]. Deposition of IC leads to the activation of complement in distal airways leading to generation of complement activation product, C5a. Alveolar macrophages release early response cytokines Tumor Necrosis Factor-α (TNF-α) and interleukin-1 β (IL-1β) which play a critical role in regulating neutrophil recruitment and neutrophil survival during ALI [5,10,11].

There is ample evidence demonstrating that immune complexes play an important role in human inflammatory lung diseases including ALI. Dr Ward described clinical evidence of immune complexes in various inflammatory diseases of the lung [12]. IC-induced ALI is a neutrophil-dependent lung injury. In addition to neutrophils [13, 14], GABA_B_Rs are also expressed in the lung [15–17] but their role in regulating neutrophil-mediated lung injury has not been previously reported. GABA_B_Rs are constitutively endocytosed and sorted to the recycling pathway to be reinserted into the plasma membrane while a small fraction of receptors are degraded in lysosomes [18]. Therefore, in the current study we hypothesized that baclofen administration after initiation of IC-induced ALI would regulate ALI by preserving GABA_B_R expression.

The role of GABA signaling in the lung has been considered for over two decades. In their review on GABAB receptors in the lung, Chapman *et al* discuss the effects of GABA_B_ agonists on multiple functions including smooth muscle contraction, microvascular leakage, bronchospasm and cough [15]. GABA receptor signaling has been documented in pulmonary cells including airway epithelium and smooth muscle cells [16, 19, 20]. GABA receptors are also expressed in alveolar type II epithelial cells [21]. GABA receptor activation was found to decrease ventilator-induced lung injury in rats [22], while lung inflammation was reduced in LPS-induced lung injury in rats through activation of GABA A receptor [23]. These mechanisms have also been implicated in pulmonary injury induced by hyperbaric oxygen [24]. Therefore, it appears that GABA involvement in lung injury is not model dependent. However, the exact mechanisms by which GABA may influence ALI remain uncertain and effects on cell permeability are being considered. In fact, the GABA A receptor has been implicated in control of endothelial cell permeability [25]. It has also been postulated that lung injury in humans triggers the activation of developmental pathways that promote inflammation and disrepair. This is intriguing considering that GABA, through GABA receptors, was shown to accelerate fetal lung development, likely through an enhanced cell proliferation, and importantly, through fluid secretion [26]. Finally although baclofen has not been tested in humans with acute lung injury, others have reported that baclofen reduces bronchial hyperreactivity in humans by inhibiting release of pro-inflammatory substance p [27]. As a role for GABA_B_Rs in regulating IC-induced ALI has not been established, in the present study we investigated the specific role of GABA_B_R in ALI, using baclofen as a specific agonist and CGP52432 as a specific GABA_B_R antagonist.

Multiple mechanisms of ameliorating IC-induced lung injury have been defined. Studies using neutralizing antibodies or selectin-Ig chimeric proteins in IC-induced models of ALI, as well as in selectin-/- mice, demonstrated the importance of selectins in neutrophil recruitment and ALI [28, 29]. Anti-TNF-α and anti-IL-1β antibodies have been shown to inhibit IC-induced ALI by blocking lung NFκB activation [30]. Pulmonary ICAM-1 expression has been shown to contribute to IC-induced ALI as treatment with anti-ICAM-1 antibody reduced vascular permeability by 48% and reduced neutrophil recruitment by 40% as measured by MPO buildup in the lungs [31, 32]. However, therapies based on these basic research findings have yet to be tested in patients. Therefore, there is a need to find existing FDA approved drugs that can serve as effective therapies to treat varying degrees of ALI/ARDS. Baclofen is an FDA approved drug used in the treatment of pain management [33] and alcohol addiction [34], and in the current study we evaluated its role in regulating IC-induced ALI.

Our studies demonstrated a protective role for baclofen by ameliorating IC-induced ALI. Baclofen prevented loss of GABA_B_R2 expression after ALI and significantly inhibited release of pro-inflammatory modulators TNF-α and Interleukin-1 receptor accessory protein (IL-1R AcP), while concurrently promoting BAL neutrophil apoptosis. Therefore, GABA_B_R2 agonists might serve a therapeutic role in treating patients with ALI by controlling neutrophil-mediated lung tissue damage.

## Materials and Methods

### Reagents

Rabbit polyclonal IgG anti-bovine serum albumin was purchased from MP Biochemical. Alexa fluor 488 conjugated bovine serum albumin was obtained from Invitrogen. Anti-β-actin, anti-pERK and anti-p38 MAPK antibodies were purchased from SantaCruz Biotechnology. Anti-GABA_B_R2, anti-pCREB, and anti-active caspase-3 antibodies were obtained from Cell Signaling Incorporated. CGP52432 was obtained from Abcam (Cambridge, MA, USA). Anti-GABA_B_R2 antibody was obtained from Thermo Scientific (Rockford, IL). Hematoxylin and eosin was obtained from Fischer Scientific (Pittsburgh, PA, USA). Anti-GAPDH and anti-TNF-α, and the ApopTag (TUNEL kit) were obtained from Millipore. All other reagents utilized in this study were obtained from Sigma Aldrich (St Louis, MO, USA) unless otherwise stated. Naphthol AS-D chloroacetate esterase (NACE) kit was obtained from Sigma Aldridge.

### Ethical Statement

Animals work reported in the manuscript was performed after approval of the protocol by University of Louisville Animal Care Use Committee (IACUC).

### Immune complex (IC) deposition induced acute lung injury (ALI) in rats

Pathogen-free male Long-Evans rats (250–300 g) (Harlan Sprague-Dawley, Indianapolis, IN) were subjected to ALI as previously described [5,6,35]. Rabbit polyclonal immunoglobulin G anti-bovine serum albumin (BSA; 2.5 mg, MP-Biomedics, Solon, OH) was injected intratracheally in 0.3 ml of phosphate buffered saline(PBS), pH 7.4. Immediately thereafter, 10 mg of BSA (Sigma-Aldridge, St Louis, MO) with 250 μg of fluorescein (FITC)-BSA (Invitrogen, Carlsbad, California) in 0.5 ml of PBS were injected intravenously. In control rats, 0.3 ml saline was administered intratracheally while 10 mg of BSA in 0.5 ml of PBS with trace amount of FITC-BSA (0.25 mg/50 μl) was injected intravenously. Four hours after initiation of IC-induced ALI, rats were administered isofluorane 3%-5% by inhalation and were administered ketamine/xylazine, 80/10 mg/kg prior to exsanguination and thoracotomy. Animal care was performed following the National Institute of Health guidelines. Animal studies were approved by the University of Louisville (Louisville, KY) Institutional Animal Care and Use Committee.

### Intervention studies

For intervention studies, 1 mg/kg of baclofen was instilled intratracheally 2 hours after initiation of lung injury, while saline was administered to control animals. In separate experiments involving GABA_B_R antagonist, CGP52432, 1 mg/kg was instilled intratracheally 1 h prior to subjecting animals to ALI. Two hours after initiation of ALI, animals were then treated with saline or 1 mg/kg baclofen, and after 2 additional hours animals were sacrificed for collection of lung tissue.

### Collection of bronchoalveolar lavage fluid (BALF)

The pulmonary circulation was flushed *via* the pulmonary artery with 5 ml of PBS, bronchoalveolar lavage fluid (BALF) was collected with a syringe, and the lungs were surgically dissected. BALF was subjected to centrifugation and BAL cells (95% neutrophils in lung injured rats) were separated from BAL cell supernatants. A small volume of cells were subjected to cytospin and stained with Wright-Giemsa Stain. The remaining cells were lysed in a lysis buffer containing 1% (vol/vol) Nonidet P-40, 10% (vol/vol) glycerol, 137 mM NaCl, 20 mM Tris·HCl, pH 7.4, 1 μg/ml aprotinin, 1 μg/ml leupeptin, 5 mM PMSF, 20 mM NaF, 1 mM sodium pyrophosphate, 1 mM sodium orthovanadate, and 1% (vol/vol) Triton X-100. Lung tissue was frozen in liquid nitrogen and homogenized in the above lysis buffer and subjected to centrifugation to separate cell debris from the solubilized lung cell lysate. Protein estimation was performed on cell lysates and supernatants, and samples were subjected to western blot analysis with appropriate antibodies.

### Western Blot analysis

Proteins from lung tissue homogenates and BAL cell lysates and supernatants were separated by 10% SDS-PAGE or 4%-12%-SDS-PAGE, and immunoblotting was performed as previously described [35].

### Assessment of lung injury

Lung injury was quantified by measuring leakage of FITC-BSA into lung parenchyma by fluorometry, by measuring the BALF protein concentrations using a Bradford assay kit using bovine serum albumin standards (BioRad). Moreover, IC-induced lung damage was also assessed by performing lung histology analysis with Hematoxylin and Eosin (H&E) staining. H&E stained rat lung tissue sections were scanned using Aperio ScanScope (Aperio Technologies, Vista, CA, USA). Scanned images of the rat lungs at 1.8x magnification which covered 95–100% of the area of the lung were used to determine lung injury. The extent of lung injury was determined by five scorers in a blinded fashion using an injury scale of 0 to 8, where 0–2 was considered a normal lung 3 to 5 is moderate lung injury and 6 to 8 was severe lung injury.

### TUNEL and Naphthol AS-D chloroacetate esterase (NACE) staining

For TUNEL and NACE analysis, unlavaged lungs were fixed in 3.7% paraformaldehyde overnight. Fixed tissue was paraffin embedded and tissue section slides were made. These slides were stained using the NACE staining kit (Sigma Aldridge) according to the manufacturer’s protocol. The slides were also subjected to a TUNEL assay using an *in situ* cell-death detection kit obtained from Millipore according to manufacturer’s protocol.

### **GABA**_B_ **R2 immunohistochemistry (IHC) staining of human control and injured lung tissue sections**

Human control lung tissue section was obtained from a patient with peripheral nodule who had a wedge biopsy with unremarkable lung tissue included. Since patients with clinical acute lung injury with pulmonary edema and respiratory failure are rarely biopsied pending their stabilization, those who get in the organizing phase get biopsied and are characterized as pathological ALI (n = 4). These lung tissue sections were obtained from the files of the Department of Pathology at University of Louisville Hospital. They were immunostained by anti-GABA_B_R2 antibody at the Special Procedures Laboratory at the University of Louisville. Tissue sections were processed for IHC using the Leica Bond III automated system. Heat induced epitope retrieval was carried out for 20 minutes using Leica’s Epitope Retrieval 1 solution. A 1:100 dilution of the primary antibody (goat anti-rabbit GABA_B_R2 antibody, Thermo scientific, Rockford, IL) was used and proteins were detected using DAB (3,3’-diaminobenzidine) Refine kit with the standard Bond Polymer Refine Detection protocol. The slides were reviewed by the a pulmonary pathologist (Dr. Mostafa Fraig) and scored as to the degree of staining and extent of staining within different types of epithelium in the lung (bronchial vs. alveolar) using a semiquantitative method, where 0 sore was used for negative staining and weak focal staining involving 33% of cells is 1+, between 33–66% positive staining is 2+ and staining above 66% is 3+.

### Mass spectrometry analysis of BAL fluid (BALF)

To achieve greater sensitivity for identification of low-abundance proteins, BALF supernatants were immunodepleted of seven high-abundance serum proteins (albumin, IgG, transferrin, fibrinogen, IgA, [alpha]-2 macroglobulin, IgM) using a ProteomeLab IgY R7 LC-2 column according to manufacturer guidelines. Proteins in the flow-through fractions were reduced, alkylated, and digested with trypsin, as previously described [36, 37]. Tryptic peptides were separated by two-dimensional liquid chromatography and analyzed by a LTQ linear ion trap mass spectrometer (Thermo Fisher Scientific, Waltham, Mass). The acquired mass spectrometry data were searched against a rat RefSeq protein database modified to include BSA using the SEQUEST (version 27 revision 11) algorithm, as described previously [36, 37]. The database analysis was performed with SequestSorcerer (Sage-N Research, San Jose, Calif), and high-probability peptide and protein identifications were assigned from the SEQUEST results using the ProteinProphet (tools.proteomecenter.org/software.php) and SageN Sorcerer statistical platforms. Scaffold 3 proteomic analysis software (ProteomeSoftware, Inc, Portland, Ore) was used for quantitative comparison using a label-free spectral counting method [36. 37].

### Statistical analysis

Statistical analysis was performed using ANOVA with post-hoc t-test. “n” indicates the number of animals per group. The number of animals used for each group varied from 3 animals to 8 animals per group. The exact number of animals used per group in each experiment is listed in the results section. Data were expressed as mean ± SEM and were averaged, analyzed and graphed. Statistical significance was assumed at p ≤ 0.05.

## Results

### Baclofen ameliorates IC-mediated ALI

**Fig 1:**
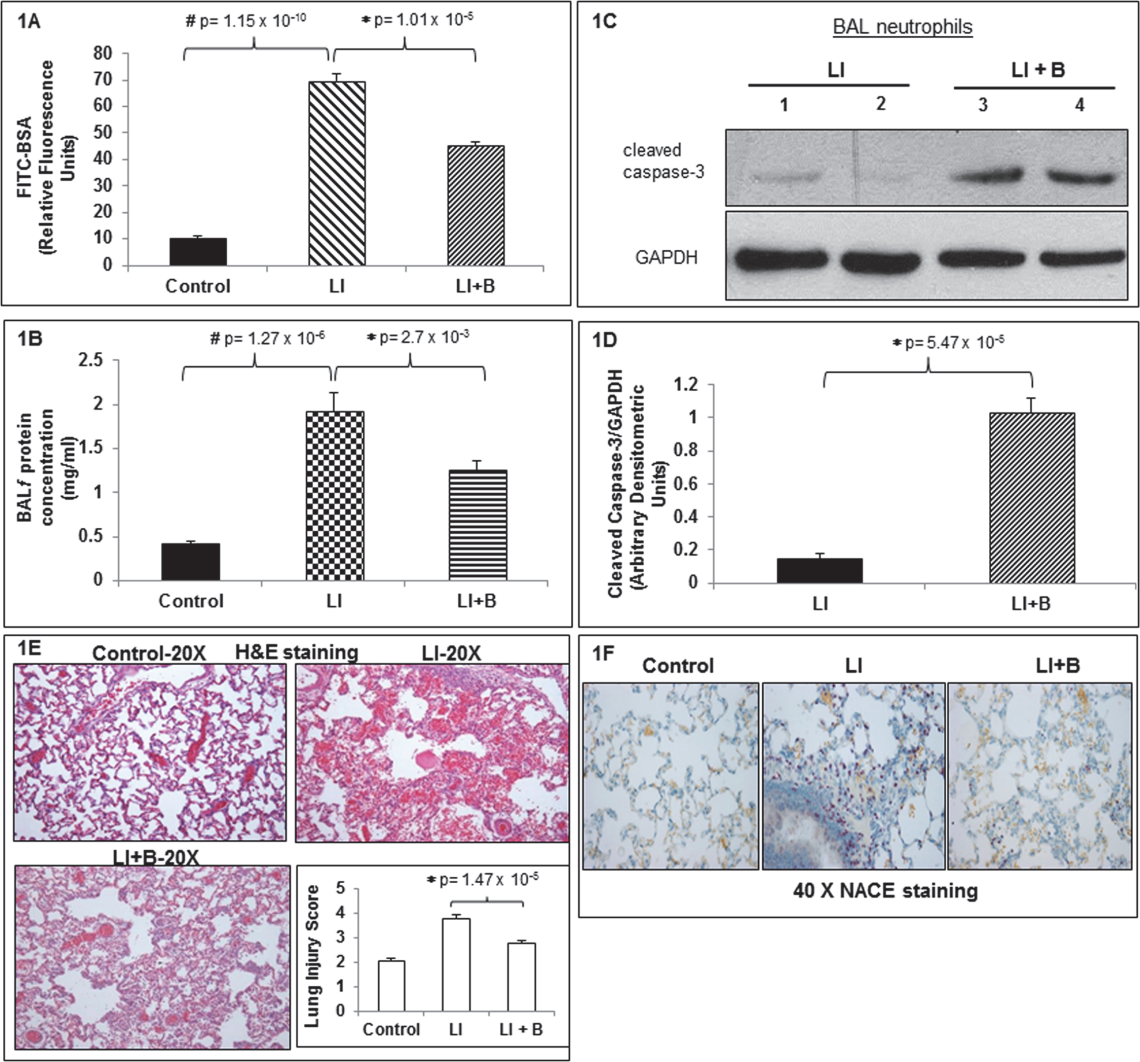
Baclofen ameliorates Immunoglobulin G-immune complex (IC) deposition induced acute lung injury (ALI). (A) Fluorescence of FITC-BSA was measured in BALF samples from three animal groups: Control, lung injury (LI), or LI followed by baclofen administration (LI+B). Data is expressed as mean relative fluorescent units ± SEM, n = 7 animals per group. (B) Total protein was measured in BALF samples from three animal groups: control, lung injury (LI), LI followed by baclofen (LI+B). Data are expressed as mean ± SEM, n = 7 animals per group. (C) Bronchoalveolar lavage cells collected from two animal groups: lung injury (LI) (lane 1), or LI followed by baclofen administration (LI+B) (lanes 2 and 3), were lysed and proteins were separated by SDS-PAGE followed by immunoblot analysis for cleaved caspase-3. A representative immunoblot from four independent experiments performed with 4 animals per group. (D) We have quantitated caspase-3 cleavage data from 4 independent experiments using 4 animals per group. LI promoted neutrophil survival while baclofen treatment after initiating LI induced BAL neutrophil apoptosis. (E) This is a representative image of Hematoxylin-Eosin (H&E) staining that was performed on lung tissue sections from 3 animal groups: control, LI and LI +B with 4 animals in control group and 8 animals each in LI and LI+B groups. Magnification 20X. IC deposition significantly increased lung injury score while baclofen treatment after initiating ALI significantly inhibited the lung injury score (bottom right panel). **(E)** This is a representative image of Naphthol AS-D chloroacetate esterase staining that was performed on lung sections from 3 animal groups: control, LI and LI+B with 3 animals per group. Magnification 40X.

To determine the effects of baclofen administration on ALI, three groups of rats were used. A control group received intratracheal saline while BSA with trace amounts of FITC-labeled BSA was administered intravenously. The other two groups received intratracheal anti-BSA while BSA and trace amounts of FITC-labeled BSA were administered intravenousy. Two hr after initiating ALI, one group received intratracheal saline while second group received intratracheal 1 mg/kg baclofen. After additional 2 h rats were killed and bronchoalveolar lavage (BAL) was performed, or lung tissue obtained for histology. Fig 1A shows the BALF fluorescence resulting from leakage of FITC-labeled BSA from blood into the lungs. ALI was associated with a significant increase in BALF fluorescence compared to control that was significantly ameliorated in animals treated with baclofen. The data was averaged and quantitated using 7 animals per group. Fig 1B shows total protein in BALF from control rats and with rats subjected to ALI with or without baclofen administration 2 h after initiating ALI. ALI was associated with significant increase in BALF protein compared to control that was significantly decreased after baclofen treatment. The average number of neutrophils was very low in the control animals (0.13 x 10^6^ ± 0.015 x 10^6^, n = 7 animals). ALI was associated with a significant increase in total neutrophils (6.8 x 10^6^ ± 0.8 x 10^6^, n = 7 animals) that was not significantly altered by administration of baclofen (8.25 x 10^6^ ± 2.05 x 10^6^, n = 7 animals). Thus, baclofen administration significantly reduced the increased vascular permeability and total protein concentrations without affecting neutrophil infiltration in BALF. Next we determined the survival status of cells (neutrophils) in BALF from animals subjected to ALI with or without baclofen treatment using 4 animals per group. We did not include a control lane as no neutrophils are present in the BALF of control animals. BAL cells from animal groups subjected to ALI with or without baclofen were lysed and equal protein amounts were subjected to anti-cleaved caspase-3 (marker of apoptosis) immunoblot analysis. As expected in Fig 1C we see little to no caspase-3 cleavage in BAL neutrophils from lung injured animals (Fig 1C) suggestive of BAL neutrophil survival (delayed neutrophil apoptosis) and more inflammation. In contrast, baclofen treatment after initiating ALI, promoted induction of caspase-3 cleavage (Fig 1C) in BAL neutrophils, suggestive of BAL neutrophil apoptosis and resolution of inflammation. We have quantitated caspase-3 cleavage data from 4 independent experiments performed with 4 animals in each of the two groups tested (Fig 1D). To determine effect of baclofen treatment on IC-induced lung damage, H&E staining was performed on three groups of animals control, lung injured (LI), lung injured followed by baclofen administration (LI+B). Fig 1E shows a representative image of H&E staining on lung tissue sections from three groups of animals tested with 4 animals in control group and 8 animals each in the LI and LI+B groups. H&E staining revealed interstitial and intra-alveolar edema, and hemorrhage after ALI which was markedly inhibited after baclofen administration (Fig 1E). IC deposition significantly increased lung injury score while baclofen treatment after initiating ALI significantly inhibited the lung injury score (Fig 1E).

NACE staining was performed on the lung tissue sections on the 3 groups of animals mentioned above to determine neutrophil infiltration in the lung tissue after ALI. Fig 1F shows a representative image of NACE staining performed on lung tissue sections from the three groups of animals with 3 animals per group. NACE staining demonstrated massive neutrophil infiltration into lung tissue after ALI compared to control. Interestingly, as opposed to unaltered neutrophil migration in BALF, baclofen administration after IC deposition markedly inhibited neutrophil infiltration in the lung tissue possibly due to decreased neutrophil trafficking in the lung tissue, decreased neutrophil chemotactic activity in the lung tissue or due to increased rate of neutrophil apoptosis in the lung tissue as seen in Fig 1C. The precise mechanisms underlying decreased neutrophil infiltration in the lung tissue after baclofen administration remains to be determined.

### Baclofen inhibits pulmonary TNF-α expression in lung injured animals

**Fig 2:**
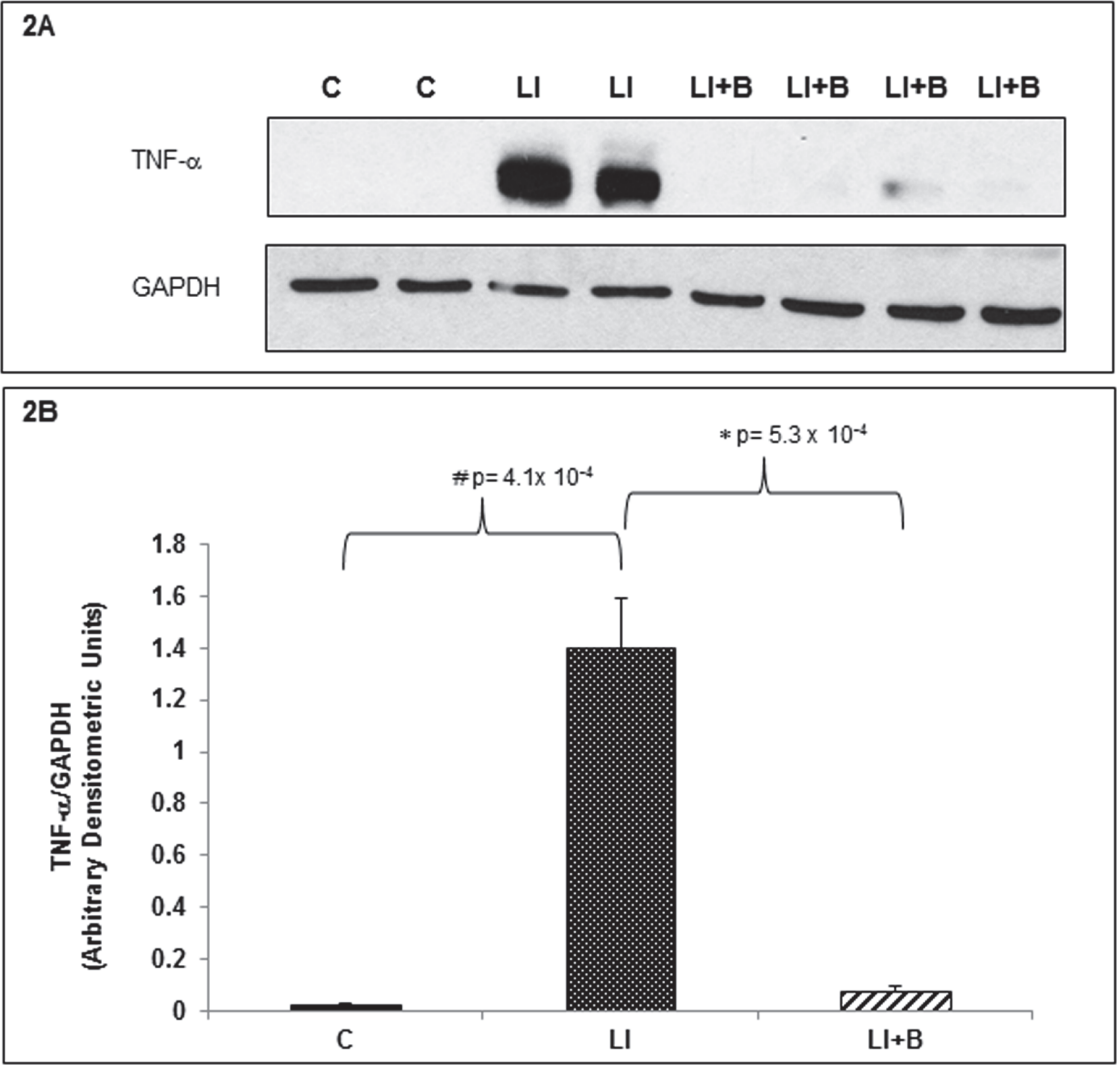
Baclofen inhibits pulmonary TNF-α expression in lung injured animals. (A) Lung tissue homogenates from control, LI and LI+B treated animals were immunoblotted with anti-TNF-α and anti-GADH antisera. GAPDH was used as an internal control. Four animals per group were used. (B) Densitometric units of TNF-α for all 4 animals/per group were normalized to their corresponding GAPDH densitometric units. The data was averaged, analyzed and quantitated.

While we have not identified the cellular target of baclofen, we examined changes in pro-inflammatory mediator TNF-α in the control lung tissue and in the injured lung tissue before and after baclofen treatment. Fig 2A and 2B demonstrates that compared to the control lung tissue, TNF-α expression was markedly increased in the lung tissue after ALI and this was significantly blocked by baclofen administration suggesting a global baclofen effect on the injured lung tissue and confirming the therapeutic potential of baclofen in ALI. Four animals were used in each of the three groups tested.

### Baclofen inhibits release of pro-inflammatory mediators into BALF

**Fig 3:**
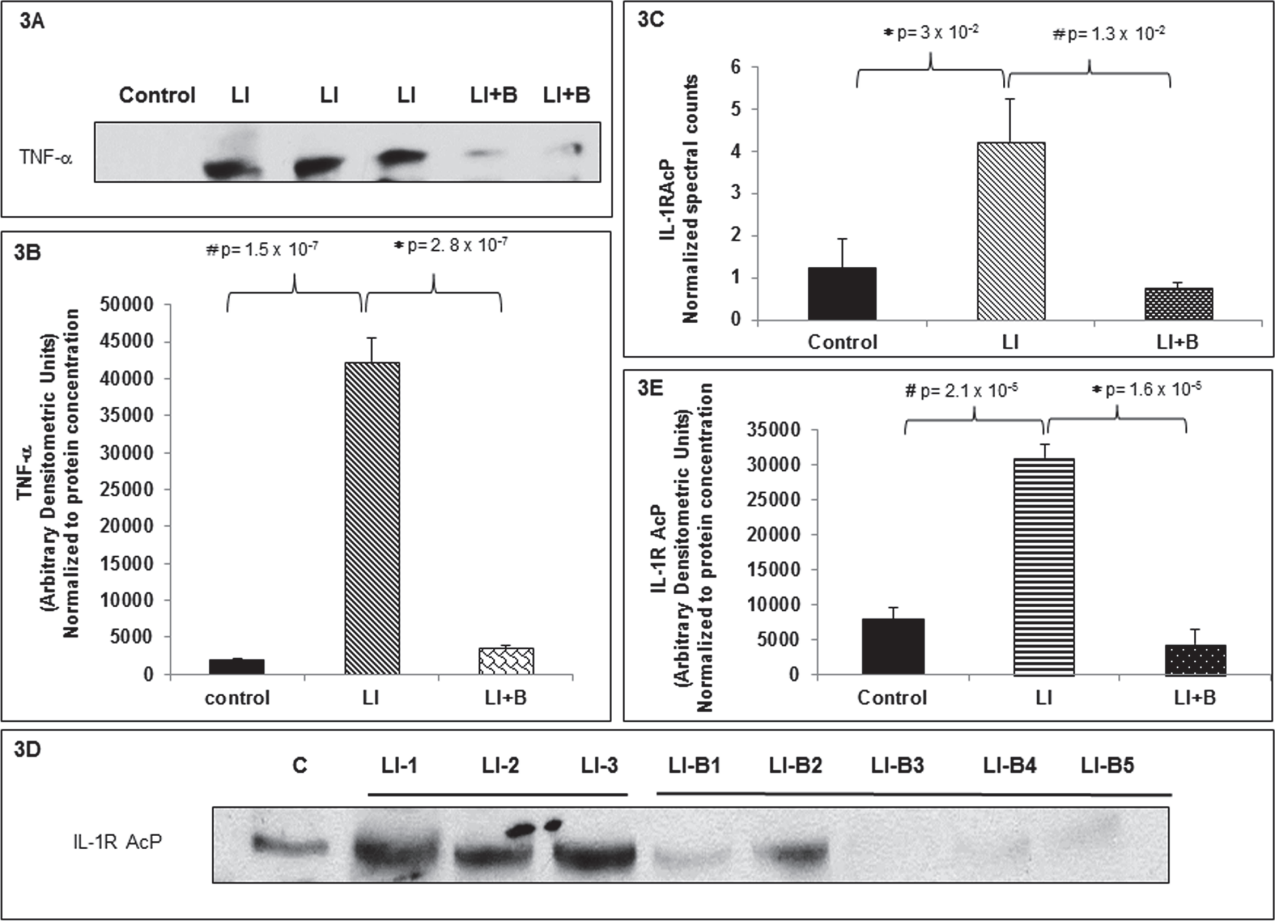
Baclofen inhibits release of pro-inflammatory mediators in BAL supernatants of lung injured rats. (A) Bronchoalveolar lavage fluid (BALF) was centrifuged to separate BAL cells from BAL supernatant. BAL supernatant was collected from three animal groups: control (lane 1), lung injury (LI) (lane 2, 3, 4), or LI followed by baclofen administration (LI+B) (lanes 5 and 6), and the proteins were separated by SDS-PAGE followed by immunoblot analysis for TNF-α. (B) TNF-α Immunoblots were scanned and band densities were quantified using ImageJ software. Arbitrary densitometric values obtained were normalized to protein concentration for each sample. Data is expressed as mean ± SEM (n = 5 animals per group). (C) BAL supernatants from three animal groups: control, lung injury (LI), LI followed by baclofen administration (LI+B) were subjected to proteomic analysis to identify and quantitate a BALF protein that was present in lung injured animals and was regulated by baclofen treatment. Interleukin-1 receptor accessory protein beta (IL-R AcP) was identified in all three animal groups tested. Spectral counts for IL-1R AcP were plotted against control, LI, LI+B groups. Data is expressed as mean ± SEM (n = 3 per group). (D) Mass spectrometry data was validated by immunoblotting BAL supernatants from three animal groups: control, lung injury (LI), or LI followed by baclofen administration (LI+B) with anti-IL-1R AcP antibody. (E) IL-1R AcP immunoblots were scanned and band densities were quantified using ImageJ software. Arbitrary densitometric values obtained were normalized to protein concentration for each sample. Data is expressed as mean ± SEM (n = 5 animals per group).

To examine potential mechanisms underlying baclofen’s protective effects on ALI we subjected BALF supernatants from control, I and LI+B treated animal groups with 5 animals per group to immunoblot analysis with anti-TNF-α antibody. Fig 3A and 3B demonstrates that TNF-α release in BALF was significantly induced after ALI as compared to control. Baclofen treatment significantly inhibited TNF-α release in BALF. The immunoblot data shown in Fig 3A was normalized to the total protein loaded in each condition as the immunoblot was performed on BALF supernatants hence an internal loading control such as β-actin or GAPDH could not be performed. Thus TNF-α arbitrary densitometric values were normalized to protein concentration for each condition.

To screen for additional inflammatory modulators regulated by baclofen, BALF supernatants from the above described 3 groups of animals with 3 animals per group were subjected to proteomic analysis. Mass spectrometry identified IL-1R AcP (gi|268607708) as a differentially regulated protein within the 3 groups tested. Fig 3C demonstrates that IL-1R AcP spectral counts were significantly increased in the BALF after ALI compared to control. Baclofen administration after initiating ALI significantly decreased IL-1R AcP spectral counts as compared to lung injured animals. We validated this proteomic data by performing immunoblot analysis on BALF supernatants. Fig 3D and 3E demonstrates that IL-1R AcP was significantly increased in the BALF after ALI, compared to control, and baclofen treatment significantly inhibited IL-1R AcP protein in the BALF. The immunoblot data shown in Fig 3D was normalized to the total protein loaded in each condition. These results suggest that baclofen administration significantly inhibits release of pro-inflammatory modulators TNF-α and IL-1R AcP in BALF of lung injured animals.

### Lung apoptosis was inhibited by baclofen

**Fig 4:**
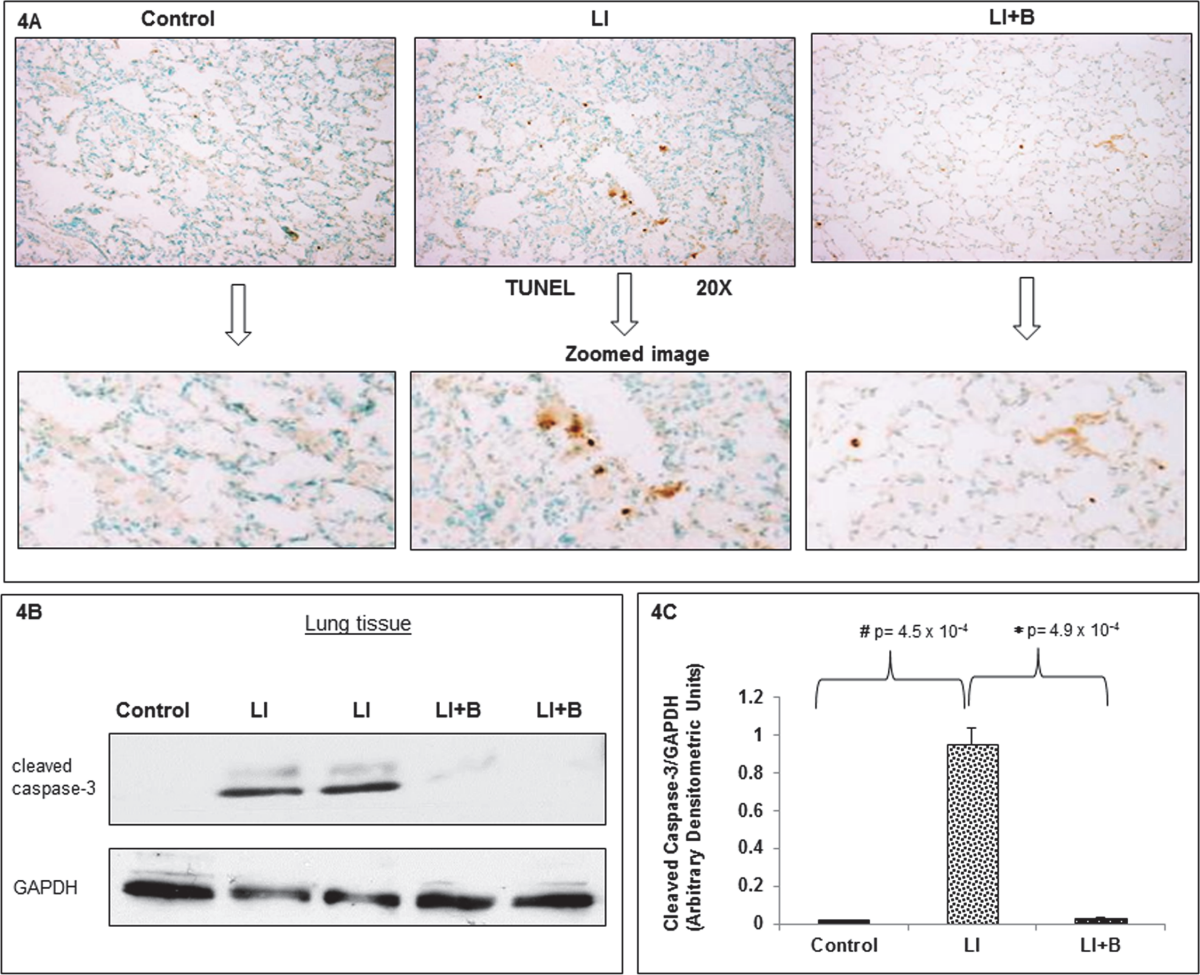
Prevention of IC deposition-induced lung apoptosis by administration of baclofen. (A) Lung tissue sections from three animal groups: control, lung injury (LI), or LI followed by baclofen administration (LI+B), were subjected to terminal deoxynucleotidyl transferase dUTP nick-end labeling (TUNEL) staining to study the changes in apoptosis in various groups. Shown are representative images with 20X images and zoomed images are shown below. N = 3 animals per group were subjected to TUNEL staining. (B) Lung tissue homogenates from three animal groups: control (lane 1), lung injury (LI) (lanes 2 and 3), or LI followed by baclofen administration (LI+B) (lanes 4 and 5), were subjected to immunoblot analysis with anti-cleaved caspase-3 and anti-GAPDH antisera. A representative blot is shown from n = 3 independent experiments performed with 3 animals per group. (C) Cleaved caspase-3 and GAPDH immunoblots were scanned and band densities were quantified using ImageJ software. Cleaved caspase-3 band density is normalized to its respective GAPDH band intensity. Data is expressed as mean ± SEM (n = 3 animals per group).

Having documented amelioration of lung damage and induction of BAL neutrophil apoptosis after baclofen treatment, we next wanted to determine levels of apoptosis in the lung tissue sections and lung tissue homogenates in the presence and absence of baclofen administration. Fig 4A demonstrates TUNEL staining of lung tissue sections from the three groups of animals with 3 animals per group. The evaluation of lung tissue sections revealed increased TUNEL staining in ALI tissue sections compared to control lungs. Baclofen after IC deposition demonstrated a decrease in TUNEL positivity (Fig 4A). Lung tissue homogenates from the 3 groups of animals with 3 animals per group were immunoblotted for cleaved caspase-3, a marker of apoptosis. Cleaved caspase-3 was detected in the lung from ALI animals, while little or no cleaved caspase-3 was present in controls or in animals receiving baclofen. These results indicate that baclofen administration prevents ALI induced apoptosis in lung tissue.

### **Baclofen administration prevents ALI induced decreases in lung GABA**_B_ **R expression which is blocked in the presence of GABA**_B_ **R antagonist**

**Fig 5:**
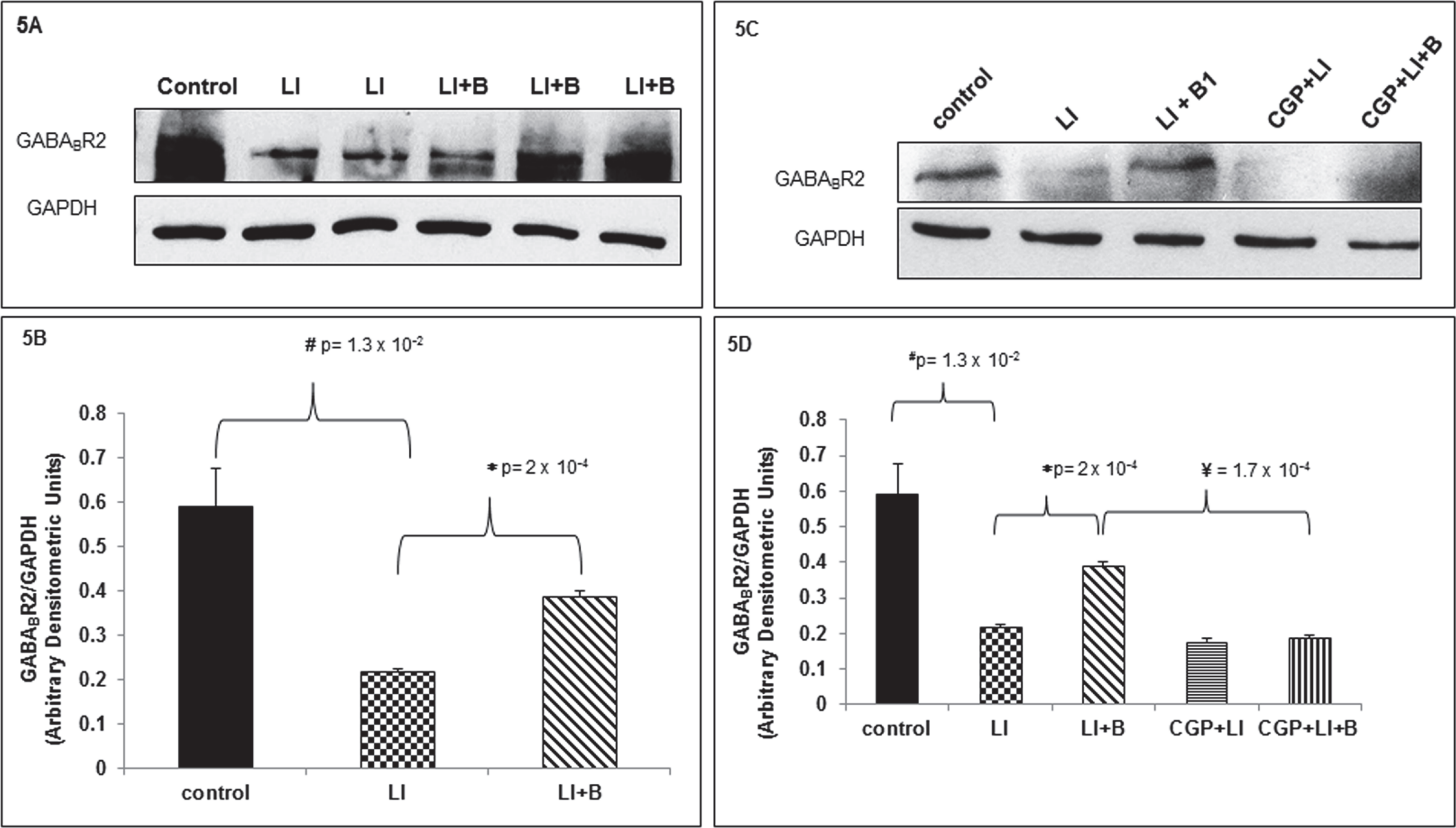
Baclofen reverses altered expression of GABA_B_Rs in the lung tissue from rats subjected to IC induced ALI. (A) Lung tissue homogenates from three animal groups: control (lane 1), lung injury (LI) (lanes 2 and 3), or LI followed by baclofen administration (LI+B) (lanes 4, 5, and 6), were subjected to immunoblot analysis with anti-GABA_B_R2 and anti-GAPDH antisera. (B) GABA_B_R2 and GAPDH immunoblots from above three animal groups were scanned and band densities were quantified using ImageJ software GABA_B_R2 band density is normalized to its respective GAPDH band intensity. Data is expressed as mean ± SEM (n = 3 animals per group). (C) Lung tissue homogenates from five animal groups: control, lung injury (LI), or LI followed by baclofen administration (LI+B), CGP52432 pretreatment followed by lung injury (CGP+LI), and CGP52432 pretreatment followed by lung injury and baclofen administration (CGP+LI+B) were subjected to immunoblot analysis with anti-GABA_B_R2 and anti-GAPDH antisera. (D) GABA_B_R2 and GAPDH immunoblots from above five animal groups were scanned and band densities were quantified using ImageJ software GABA_B_R2 band density is normalized to its respective GAPDH band intensity. Data is expressed as mean ± SEM (n = 3 animals per group).

While GABA_B_Rs are expressed in the lung cells any changes in its expression after IC induced ALI are unclear [15–17]. According to previous reports we demonstrate expression of GABA_B_R2 in the lung tissue of control animals. Interestingly, we demonstrate for the first time significant decrease in pulmonary GABA_B_R2 expression in animals subjected to ALI compared to control (Fig 5A and 5B). Baclofen significantly restored pulmonary GABA_B_R2 expression in animals subjected to ALI (Fig 5A and 5B). Three animals were used in each of the 3 groups tested. To examine whether baclofen exerted its effects by binding specifically to GABA_B_Rs two additional groups were examined. These two groups included animals that were pretreated intratracheally with 1 mg/kg CGP52432 (GABA_B_R antagonist) and subjected to ALI with or without baclofen treatment 2 h after initiation of ALI. CGP52432 pretreatment significantly blocked baclofen-induced preservation of GABA_B_R2 expression after ALI (Fig 5C and 5D). CGP52432 pretreatment followed by ALI decreased GABA_B_R2 expression to the same extent as ALI alone (Fig 5D). Three animals were used in each of the 3 groups tested. These results indicate that baclofen preserves GABA_B_R2 expression after ALI by binding GABA_B_Rs.

### **Regulation of GABA**_B_ **R2 expression in type II pneumocytes of human lung tissue sections**

**Fig 6:**
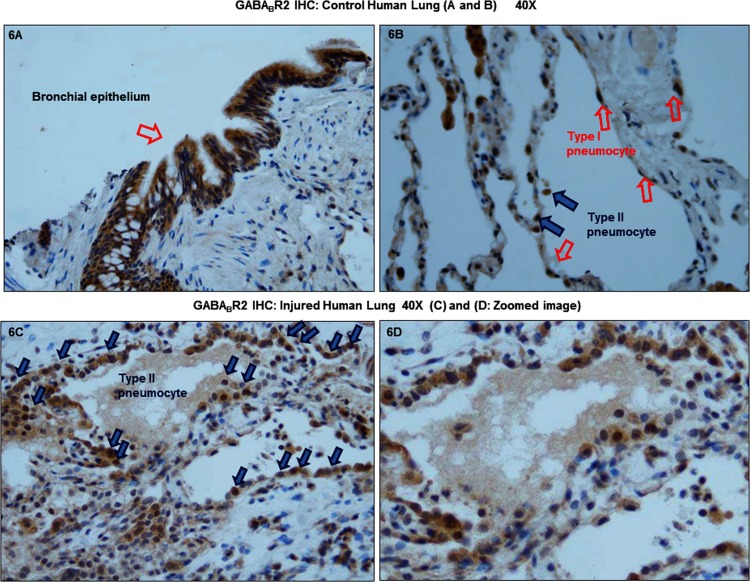
GABA_B_R2 expression is regulated in type II pneumocytes of lung tissue sections of lung injured patients compared to the controls. This is a representative image (40X) of anti-GABA_B_R2 immunohistochemistry that was performed on lung tissue sections from control and lung injured patient (lung injured patients n = 4). (A) Anti-GABA_B_R2 was detected in the bronchial epithelium of the control lungs and (B) in the type I and type II pneumocytes of control lung. (C) Increased type II pneumocytes were detected in the lungs of the lung injured patients. Moreover, GABA_B_R2 expression was mainly detected in the type II pneumocytes in these patients suggesting a physiological role for GABA_B_R2 in the repair to lung damage. (D) zoomed image of 6C.

We obtained human lung tissue sections from control and lung injured patients. These human lung tissue sections were subjected to anti-GABA_B_R2 immunohistochemistry. In Fig 6 (panel A and B) we see that in the control lung GABA_B_R2 expression is detected mainly in the bronchial epithelium and in type I and II pneumocytes. In contrast, in injured lung (Fig 6C and 6D), we see increased type II pneumocytes compared to the control lung section and GABA_B_R2 staining was mainly detected in type II pneumocytes suggesting a physiological role for GABA_B_R2 in the repair and reaction to some insults to the lung parenchyma. Considering the bronchial epithelium as an internal positive control (3+). The level of GABA_B_R2 staining in type II pneumocytes of ALI patients is 2+ and in healthy control is 0–1+.

### Baclofen administration alters signaling kinase activation in ALI

**Fig 7:**
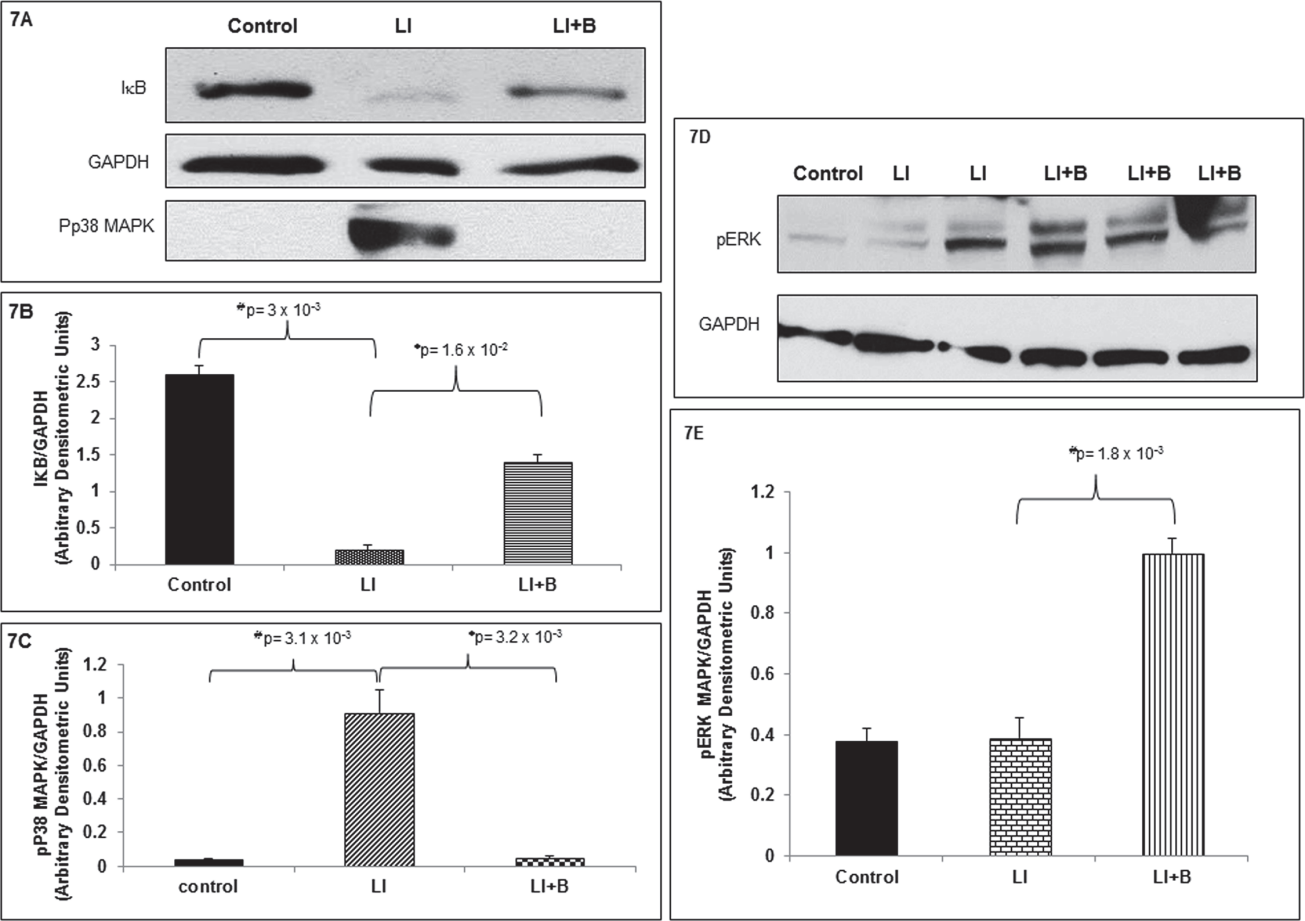
Baclofen administration alters signaling kinase activation in ALI. (A) Lung tissue homogenates from three animal groups: control, lung injury (LI), or LI followed by baclofen administration (LI+B), were subjected to immunoblot analysis with anti-I B, anti-pp38 MAPK, and anti-GAPDH antisera. (B and C) IκB, pp38 MAPK, and GAPDH immunoblots from above three animal groups were scanned and band densities were quantified using ImageJ software. Phosphorylated p38 MAPK and IκB band densities were normalized to their respective GAPDH band intensity. Data is expressed as mean ± SEM (n = 3 animals per group). (D) Lung tissue homogenates from three animal groups: control (lane 1), lung injury (LI) (lanes 2 and 3), or LI followed by baclofen administration (LI+B) (lanes 4, 5, and 6) were subjected to immunoblot analysis with anti-pERK MAPK and anti-GAPDH antisera. (E) pERK MAPK and GAPDH immunoblots from above three animal groups with 3 animals per group were scanned and band densities were quantified using ImageJ software. Phosphorylated ERK MAPK band densities were normalized to their respective GAPDH band intensity. Data is expressed as mean ± SEM (n = 3 animals per group).

IC-induced ALI results in increased lung cell apoptosis which is inhibited by baclofen treatment. Therefore, we wanted to identify signaling kinases that were activated in the lung after ALI in baclofen treated and untreated animals. Lung tissue homogenates from three animal groups with 3 animals per group were immunoblotted with, IκB, phospho-p38 MAPK, and pERK antisera. Pro-inflammatory NF-κB activation in the lung has been documented in IC-induced ALI [38, 39] and degradation of IκB, an inhibitor of NFκB, has been used in this model to document increased NFκB activation [30]. In Fig 7A we demonstrated lung IκB expression was significantly decreased after ALI compared to control, consistent with IκB degradation and NFκB activation (Fig 7A and 7B) [30, 38]. Baclofen treatment after induction of ALI preserved IκB expression (Fig 7A and 7B) suggestive of blockade of NFκB activation. In addition, in Fig 7A we demonstrated that ALI significantly increased p38 MAPK phosphorylation in the lung tissue compared to control (Fig 7A and 7C) which was significantly inhibited by baclofen treatment after induction of ALI (Fig 7A and 7C). Furthermore, ERK phosphorylation was significantly enhanced in animals treated with baclofen after induction of ALI, as compared to control or animals subjected to ALI (Fig 7D and 7E). These results suggest that baclofen administration inhibits NFκB activation (by inhibiting IκB degradation) and p38 MAPK activation while promoting ERK activation.

## Discussion

Neutrophils are known contributors of ALI/ARDS; however effective therapies to limit neutrophil induced lung injury are not available. In the current study we demonstrate that IC induced ALI is ameliorated by baclofen administration 2 h after initiation of ALI. We demonstrate for the first time release of interleukin-1β/interleukin-1 receptor (IL-1 β/IL-1R) signaling modulator, IL-1R accessory protein (IL-1R AcP) in BALFs of lung injured animals. Baclofen treatment significantly inhibited release of pro-inflammatory TNF-α and IL-1R AcP in BALF, promoted induction of BAL neutrophil apoptosis, decreased lung apoptosis, decreased vascular permeability and ameliorated ALI. H&E and NACE staining demonstrated decreased edema, hemorrhage, and decreased neutrophil staining in lung tissue of baclofen treated animals subjected to ALI. Thus, our work has identified a possible novel role for baclofen, a drug previously approved for pain management [33] and for treatment of alcohol addiction [34], to be used in the treatment of patients with ALI to control neutrophil-mediated lung tissue damage by modulating inflammatory mediators in the BALF and by promoting BAL neutrophil apoptosis.

While GABA_B_Rs are present in the lungs, regulation of GABA_B_R expression and/or activation during ALI has not been documented. For the first time we demonstrate decreased pulmonary GABA_B_R2 expression and concurrent activation of NFκB and p38 MAPK signaling pathways after ALI. There is ample evidence pointing to the importance of GABA_B_R signaling in neuronal tissues [18, 40] however, beneficial role of GABA_B_Rs in non-neuronal tissues is limited. GABA_B_Rs are constitutively endocytosed and sorted to the recycling pathway to be reinserted into the plasma membrane while a small fraction of receptors are degraded in lysosomes [18]. However, during sustained activation of NMDA and AMPA receptors such as during cerebral ischemia, GABA_B_R1 and R2 are phosphorylated and targeted for lysosomal degradation resulting in decreased GABA_B_R receptor expression at the plasma membrane [41]. Most recently GABA_B_Rs have been shown to be polyubiquitinated *via* Lys^48^ linkage and degraded by the proteasomes *via* endoplasmic reticulum associated degradation [42]. GABA_B_R2 has been shown to bind transcription factor CCAAT/enhancer-binding protein (C/EBP) homologous protein (CHOP). Binding of CHOP to GABA_B_R2 resulted in intracellular accumulation of GABA_B_Rs and in a reduced number of cell surface receptors [43]. The precise mechanisms underlying down-regulation of lung GABA_B_R2 expression after IC induced ALI remain to be elucidated.

We directly assessed expression of GABA_B_R2 in human lung tissue sections obtained from control and lung injured patients. GABA_B_R2 expression was detected in control lungs in the bronchial epithelium and in type I and II pneumocytes. In the injured lung there were increased type II pneumocytes compared to the control lung section and GABA_B_R2 staining was mainly detected in type II pneumocytes. It is important to note that pulmonary edema and respiratory failure that are hallmarks of clinical ALI and are rarely biopsied immediately as these patients need to be stabilized before undergoing biopsies. These patients are characterized by increased type II pneumocytes, edema and less fibrosis. These patients eventually end up in organizing pneumonia with diffuse alveolar damage. Type II pneumocytes are considered to be the caretakers of the alveolar compartment and respond to damage of vulnerable type I pneumocytes by dividing and replicating to replace damaged type I pneumocytes. Therefore, as expected we saw an increase in type II pneumocytes in the injured lung suggesting that tissue damage was followed by repair and these type II pneumocytes stained intensely with anti-GABA_B_R2 antibody suggesting an important physiological role for GABA_B_R2 in the repair process of lung damage. It is interesting to note that similar to what we see in humans, we observed increased GABA_B_R2 expression in lung homogenates from rats treated with baclofen after initiating IC-induced ALI. Baclofen treatment enhanced GABA_B_R2 expression in the lung and concurrently ameliorated IC-induced ALI. Our animal model may not be entirely representative of the human pathology as animal studies are performed in controlled condition, minimizing inherent human variability that may also result from behaviors such as smoking and alcohol abuse. Nevertheless, GABA_B_R2 seems unequivocally involved in lung injury response both in the human and in the animal model; although the comparison between the two may be difficult considering samples were collected later after the onset of injury in the humans. Similar to our observation, decreased renal GABA_B_R expression is also documented in a model of acute kidney injury [44]. Additionally, GABA_B_R down-regulation has previously also been shown to be associated with neurological disorders, possibly due to dysregulated receptor trafficking mechanisms and/or due to interaction with receptor associated proteins [45–49]. These changes could affect the excitation/inhibition balance of GABA_B_Rs contributing to the disease state. Baclofen treatment may be protective as it restores GABA_B_Rs in the pathological conditions of ALI and thereby promotes efficient GABA_B_R signaling inhibiting disease pathology. Beneficial effects of baclofen on ALI occurred by specifically binding to GABA_B_R as treatment of animals with CGP52432, an antagonist for GABA_B_R prevented baclofen mediated preservation of pulmonary GABA_B_R2 expression after ALI. Protective effects of baclofen *via* binding GABA_B_Rs have been shown to prevent CCl_4_ induced liver fibrosis [50] and in mustard oil-induced colon inflammation [51].

In addition to decreased lung GABA_B_R2 expression after ALI, we documented enhanced p38 MAPK phosphorylation and apoptosis in the lung tissue. Baclofen treatment restored GABA_B_R2 expression and inhibited p38 MAPK activation and lung cell apoptosis. P38 MAPK activation is associated with increased apoptosis of lung epithelial cells exposed to hydrochloric acid [52]. Moreover, the role of p38 MAPK in regulating expression of GABA_B_Rs has previously been demonstrated [53]. Down-regulation of dorsal horn GABA_B_R expression in the spinal nerve ligation model of neuropathic pain has been shown to be p38 MAPK dependent. GABA_B_Rs expression is preserved by intrathecal administration of p38 MAPK inhibitor SB203580. Furthermore enhancing GABA_B1_ receptor expression provides neuroprotection by inhibiting p38 MAPK-mediated nitric oxide induced apoptosis [54]. Baclofen treatment after IC induced ALI, restored GABA_B_R expression, inhibited p38 MAPK, and activated ERK phosphorylation. GABA_B_R agonists have been previously shown to activate ERK phosphorylation in the brain tissue as a protective mechanism [55–58]. Thus, p38 MAPK activation in response to ALI may contribute to decreased lung GABA_B_Rs and increased apoptosis of lung cells while baclofen induced ERK phosphorylation may provide a protective effect by inhibiting lung cell apoptosis. Pulmonary NFκB activity contributes to IC induced ALI [30] and p38 MAPK mediated NFκB activity has been shown to contribute to LPS-induced ALI [59–61]. Similar to previous published studies we demonstrate decreased lung IκB expression after ALI, indicative of increased NFκB activation in IC model of ALI [30].

In our studies we demonstrated that IC induced neutrophil recruitment in BALF was not altered by baclofen treatment. This was not surprising as baclofen was administered 2 h after initiating lung injury and it has previously been documented that neutrophils are recruited into the BALF within 2 h after initiating ALI [5]. Interestingly, baclofen treatment after IC deposition induced BAL neutrophil apoptosis and markedly abrogated NACE staining in the lung tissue. The decrease in NACE staining of lung injured animals treated with baclofen may be due to increased BAL neutrophil apoptosis or possibly due to decreased neutrophil trafficking in the lung tissue or due to decreased neutrophil chemotactic activity in the lung tissue. Baclofen has previously been shown to inhibit chemotactic activity of various cytokines by promoting heterologous desensitization of Gαi coupled G protein coupled receptors [62]. However, in the current study, the precise mechanisms underlying decreased neutrophil infiltration in the lung tissue after baclofen administration remains to be determined.

Baclofen was administered 2 h after initiating ALI and it is well documented in the IC model of ALI that TNF-α and IL-1β are released within minutes resulting in increased activation of selectins and adhesion molecules on endothelial cells which results in transmigration of neutrophils into the lung interstitium and alveolar compartment [29]. Moreover, TNF-α and IL-1 β signaling is critical to lung NFκB activation which is maximal 4 h after IgG immune-complex deposition [30]. Interestingly, we demonstrated a significant increase in pulmonary TNF-α expression after ALI that was significantly inhibited by baclofen administration after initiating ALI. This decrease in TNF-α expression in the lung may contribute to decreased IκB degradation/NFκB activation in the lung after baclofen administration. Moreover, within 30 m of IC deposition neutrophil recruitment is achieved, but baclofen administration 2 h after initiating lung injury blocks TNF-α and IL-1R AcP release in BALF. This blockade of TNF-α and IL-1R AcP release in BALF may also contribute to the inhibition of lung NFκB activation (documented by degradation of IκB). In addition, baclofen mediated blockage of TNF-α and IL-1R AcP release is also accompanied by BAL cell (neutrophil) apoptosis. Similar to our studies it has been demonstrated that baclofen administration inhibits TNF-α release in allergic contact dermatitis [62]. Moreover, pro-inflammatory TNF-α is known to promote neutrophil survival and inflammation in IC-induced ALI [10] and anti-TNF-α treatment has been shown to block IC-induced ALI by inhibiting neutrophil activation [30]. Furthermore, IL-1 β has previously shown to prolong neutrophil lifespan [11]. Transfection of interleukin-1 receptor1 accessory protein β (IL-1R AcP) has been shown to increase IL-1β affinity for IL-1R1 [63]. Additionally, it has been demonstrated that presence of IL-1R AcP is essential for IL-1 β to initiate signaling *via* the IL-1R1 [63]. The importance of IL-1R AcP in mediating IL-1 βsignaling was demonstrated in IL-1R AcP-deficient mice and IL-1R AcP deficient fibroblasts. Loss of IL-1R1 AcP expression decreased affinity of IL-1R1 to its ligands [63]. Thus, it is quite likely that baclofen induced blockade of TNF-α and IL-1R AcP in BALF fluid of lung injured animals, may contribute to BAL neutrophil apoptosis by blockade of pro-inflammatory IL-1β signaling. These results collectively suggest an anti-inflammatory role for baclofen in the setting of IC induced ALI by inhibiting release of TNF-α and IL-1R AcP in the BALF. Similar to our current study, GABA_B_R activation is anti-inflammatory in the setting of allergic contact dermatitis [62], autoimmune inflammation [64], and rheumatoid arthritis [65].

## Conclusions

In the current study we have demonstrated beneficial effects of baclofen, a GABA_B_ receptor agonist, in the treatment of experimental immune-complex deposition mediated ALI. Baclofen exerted its effects in treatment of ALI by preserving pulmonary GABA_B_R2 expression, by inhibiting lung cell apoptosis and by promoting BAL neutrophil apoptosis. Baclofen treatment enhanced BAL neutrophil apoptosis concurrent with inhibition of pro-inflammatory TNF-α and IL-1R AcP release in the BALF of lung injured animals. Therefore, baclofen a previously approved drug for pain management in spasticity, alcohol and drug addiction might serve a new therapeutic role in treating patients with varying degrees of ALI by controlling neutrophil-mediated lung tissue damage.

## Supporting Information

S1 Fig Baclofen treatment after initiating ALI promotes caspase-3 cleavage in BAL neutrophil. (TIF)

S2 Fig Baclofen treatment after initiating ALI inhibits pulmonary TNF-α expression. (TIF)

S3 Fig Baclofen treatment after initiating ALI inhibits TNF-α expression in BAL supernatants. (TIF)

S4 Fig Baclofen treatment after initiating ALI inhibits IL-1RAcP expression in BAL supernatants. (TIF)

S5 Fig Baclofen treatment after initiating ALI inhibits pulmonary caspase-3 cleavage. (TIF)

S6 Fig Baclofen treatment after initiating ALI preserves pulmonary GABA_B_R2 expression by binding GABA_B_R2s. (TIF)

S7 Fig Baclofen treatment after initiating ALI inhibits pulmonary pP38 MAPK. (TIF)

S8 Fig Baclofen treatment after initiating ALI preserves pulmonary I B expression. (TIF)

S9 Fig Baclofen treatment after initiating ALI promotes pulmonary pERK MAPK. (TIF)

S1 Dataset Lung score sheet #1. (XLS)

S2 Dataset Lung score sheet #2. (XLS)

S3 Dataset Lung score sheet #3. (XLS)

S4 Dataset Lung score sheet #4. (XLS)

S5 Dataset Lung score sheet #5. (XLS)

S6 Dataset Quantitation in Fig 1A, 1B, and 1D. (XLSX)

S7 Dataset Quantitation in Fig 1E. (XLSX)

S8 Dataset Quantitation in Fig 2B. (XLSX)

S9 Dataset Quantitation in Fig 3B, 3C and 3E. (XLSX)

S10 Dataset Quantitation in Fig 4C. (XLSX)

S11 Dataset Quantitation in Fig 5D. (XLSX)

S12 Dataset Quantitation in Fig 7B, 7C and 7E. (XLSX)

S13 Dataset H&E staining of lung tissue sections from control, LI, LI+B treated groups used for lung tissue scoring. (PDF)

S14 Dataset Anti-GABA_B_R2 immunohistochemistry in human control and ALI patient tissue sections. (PDF)
