# Supplementary figures and images for: Baclofen, a GABABR Agonist, Ameliorates Immune-Complex Mediated Acute Lung Injury by Modulating Pro-Inflammatory Mediators
Source: PLoS One. 2015 Apr 7;10(4):e0121637. doi: 10.1371/journal.pone.0121637 (PMC4388838; doi:10.1371/journal.pone.0121637)

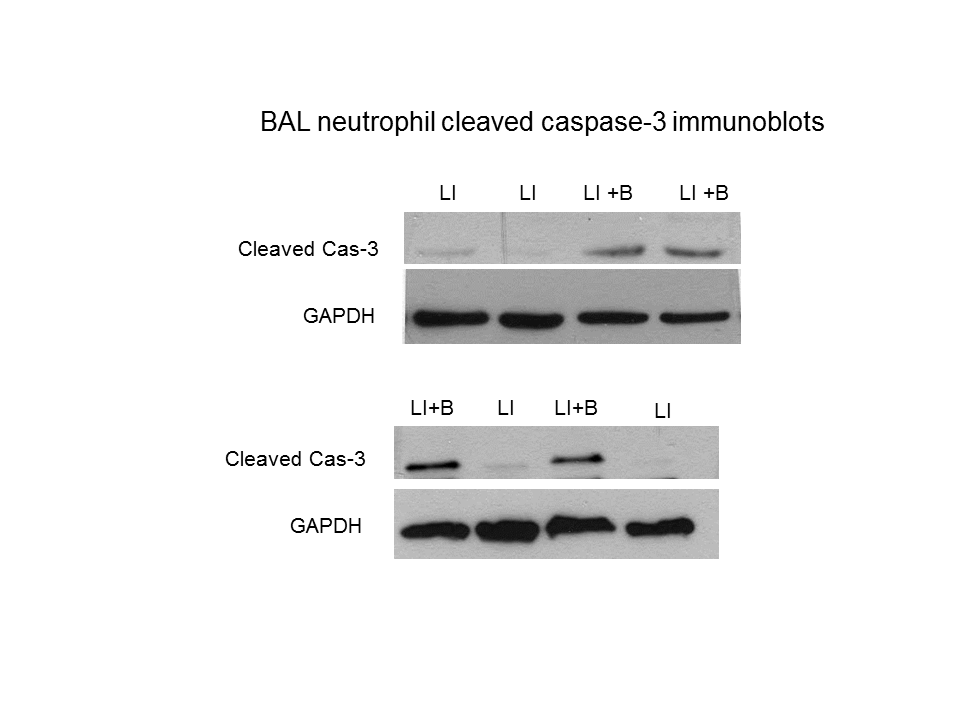

Supplement: S1 Fig — (TIF) [file pone.0121637.s001.TIF]

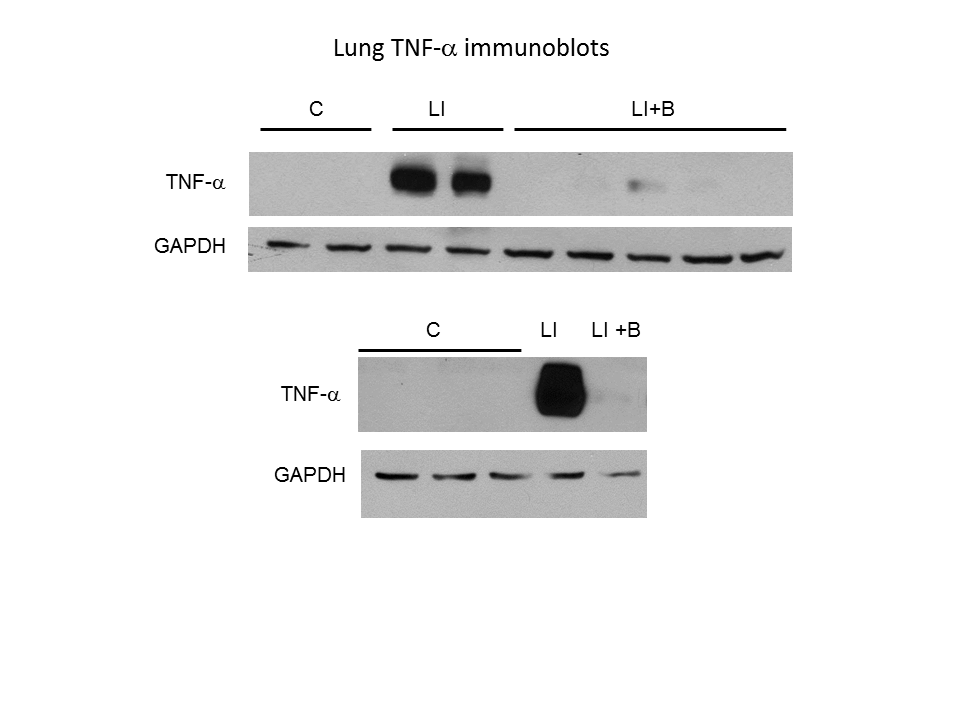

Supplement: S2 Fig — (TIF) [file pone.0121637.s002.Tif]

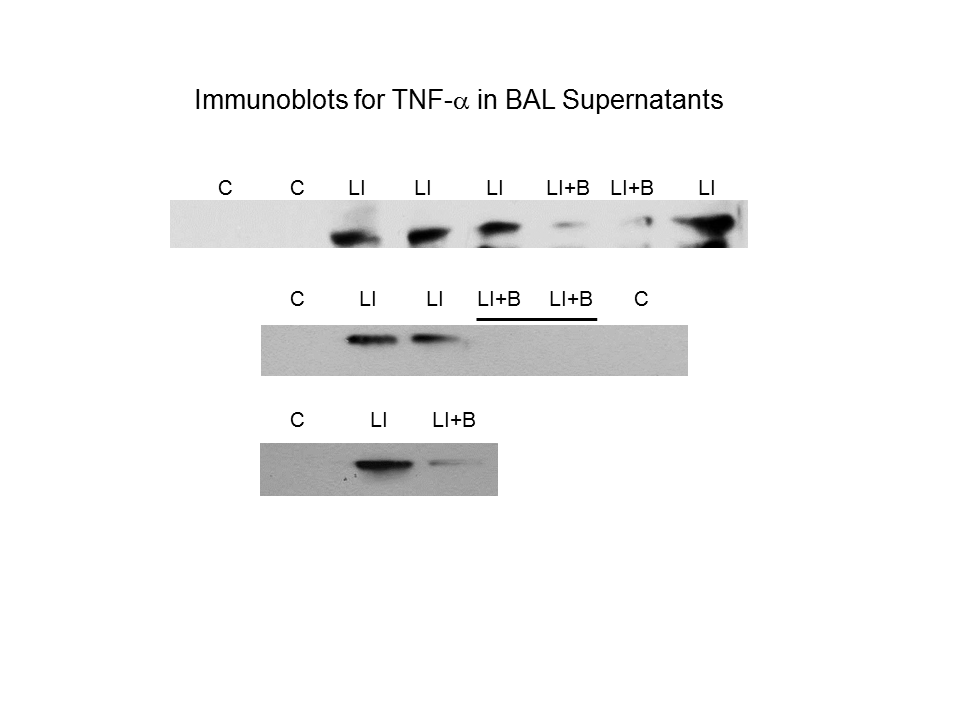

Supplement: S3 Fig — (TIF) [file pone.0121637.s003.tif]

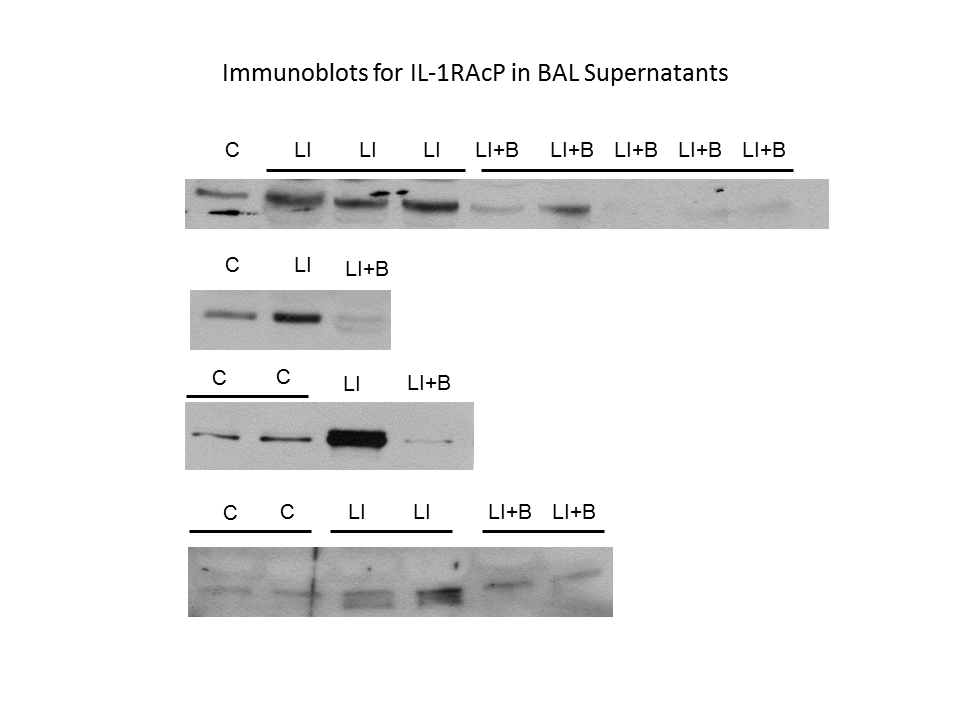

Supplement: S4 Fig — (TIF) [file pone.0121637.s004.tif]

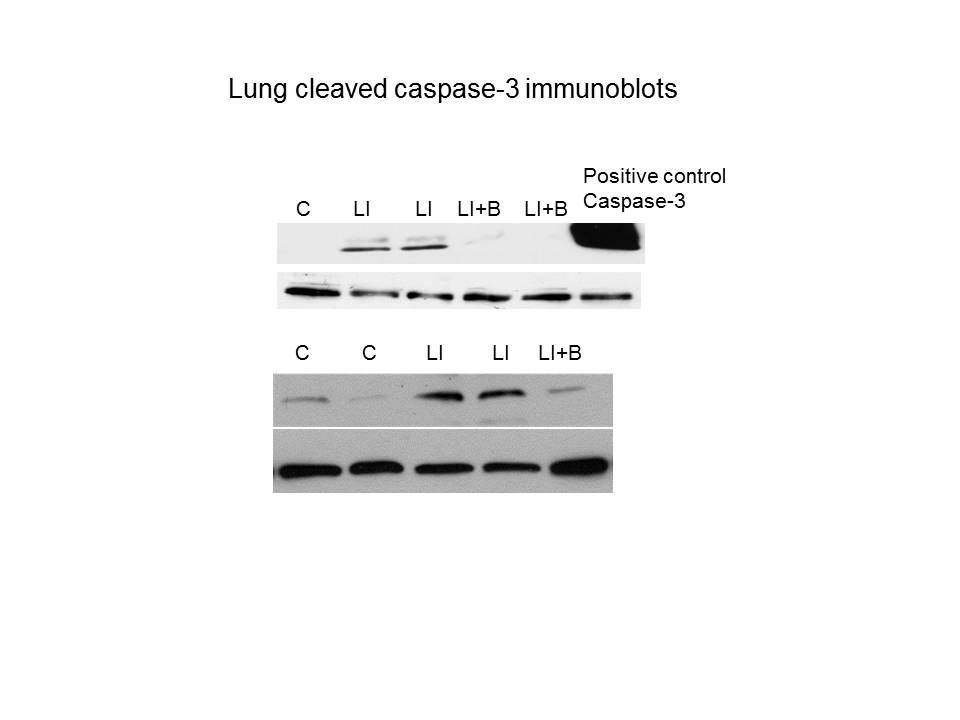

Supplement: S5 Fig — (TIF) [file pone.0121637.s005.tif]

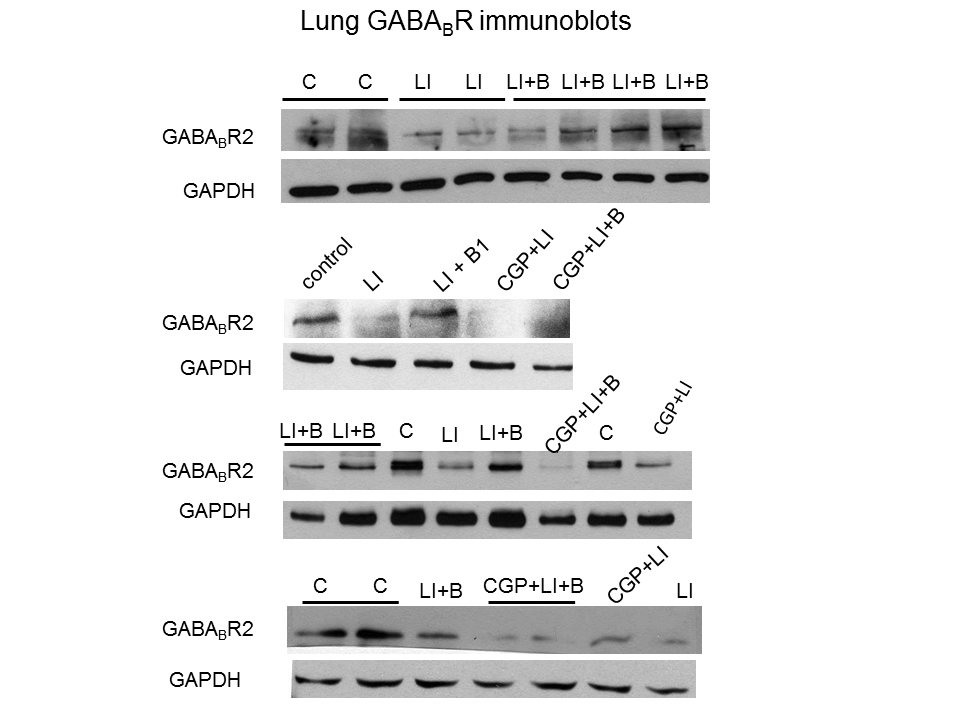

Supplement: S6 Fig — (TIF) [file pone.0121637.s006.tif]

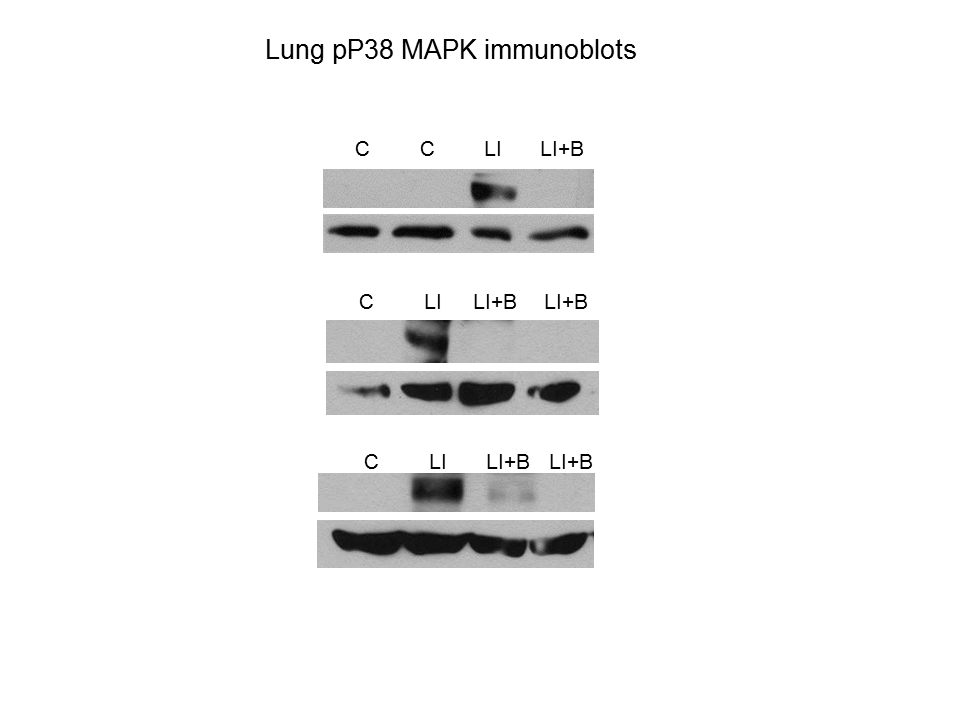

Supplement: S7 Fig — (TIF) [file pone.0121637.s007.tif]

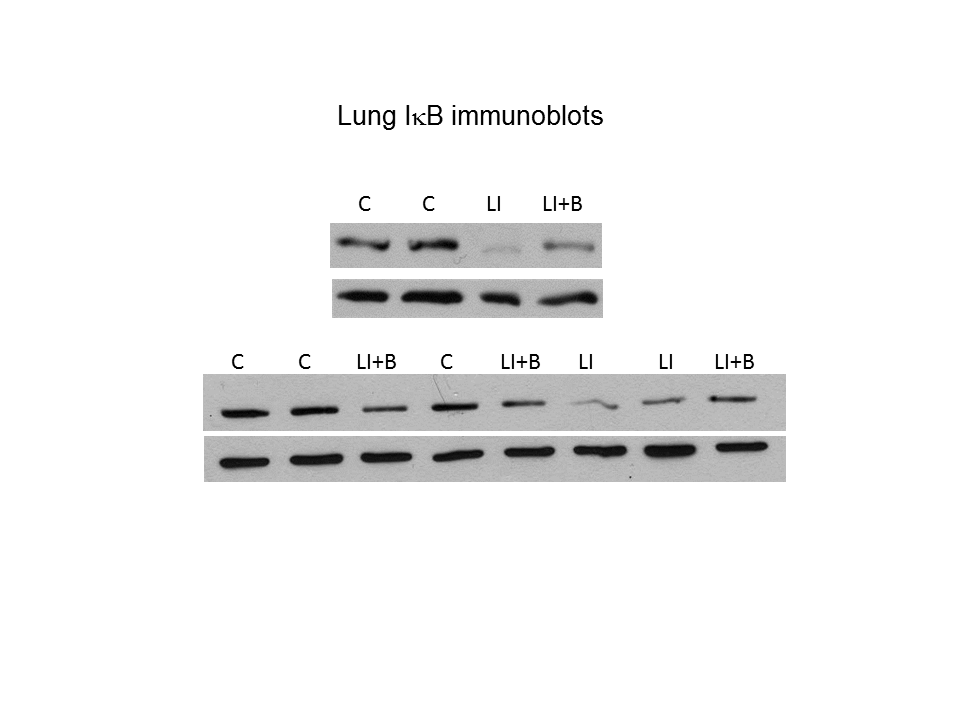

Supplement: S8 Fig — (TIF) [file pone.0121637.s008.tif]

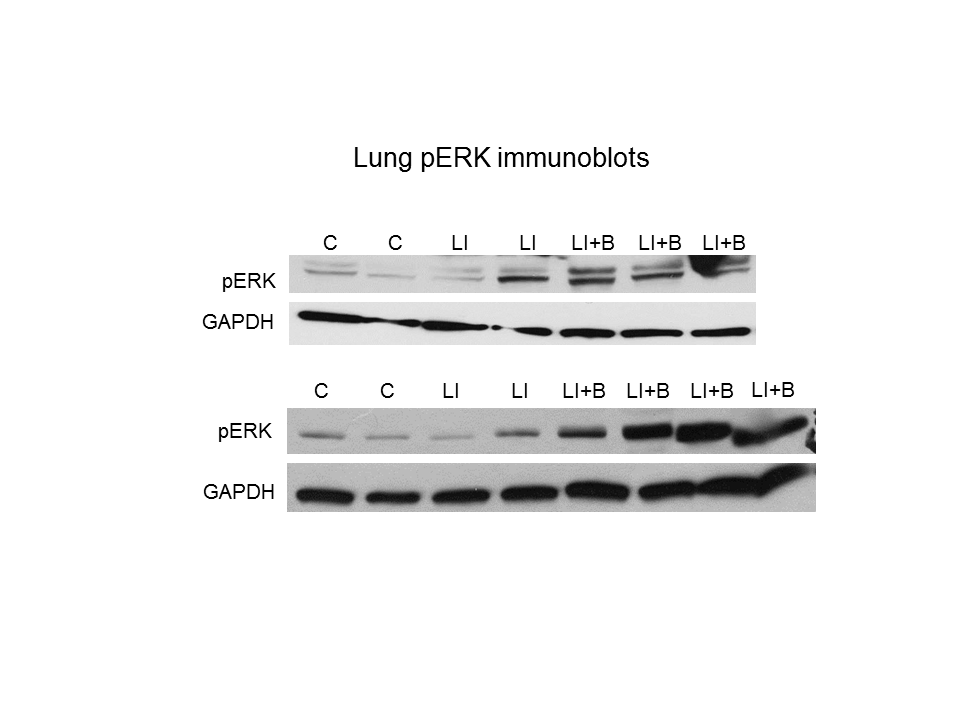

Supplement: S9 Fig — (TIF) [file pone.0121637.s009.tif]
