# Supplementary material for: Baclofen, a GABABR Agonist, Ameliorates Immune-Complex Mediated Acute Lung Injury by Modulating Pro-Inflammatory Mediators
Source: PLoS One. 2015 Apr 7;10(4):e0121637. doi: 10.1371/journal.pone.0121637 (PMC4388838; doi:10.1371/journal.pone.0121637)

1

Slide # 1

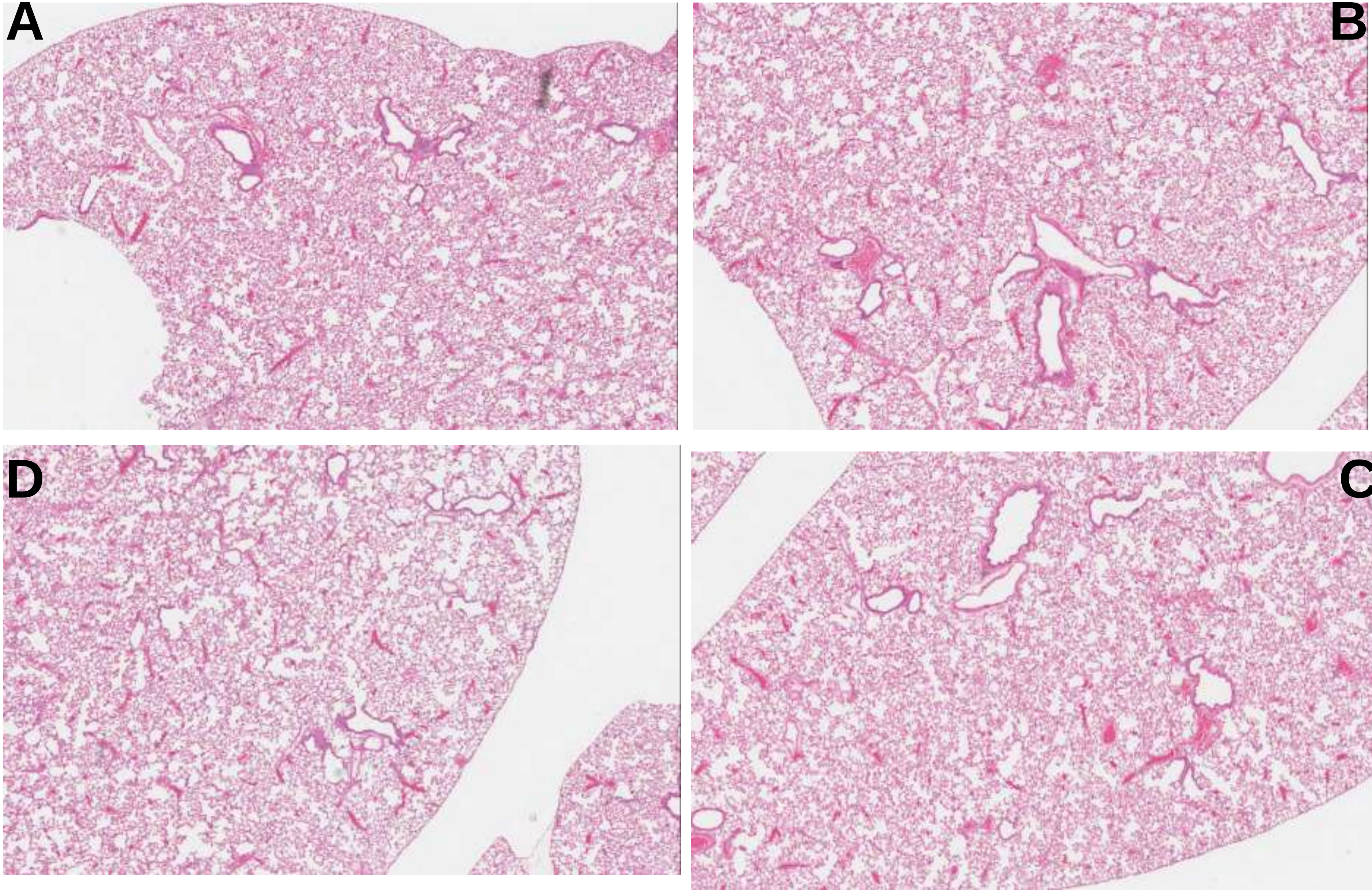

Slide # 1

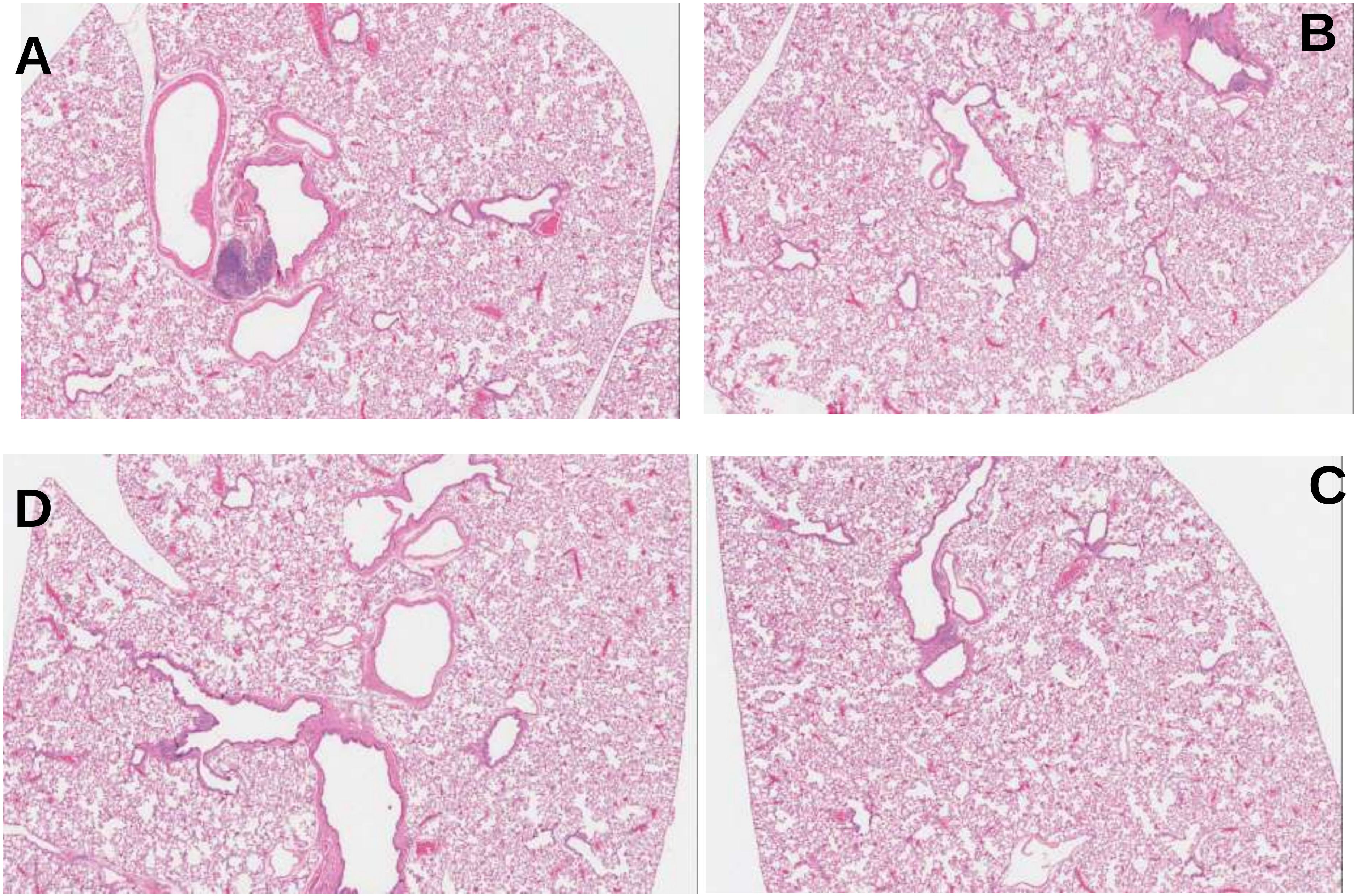

Slide # 2

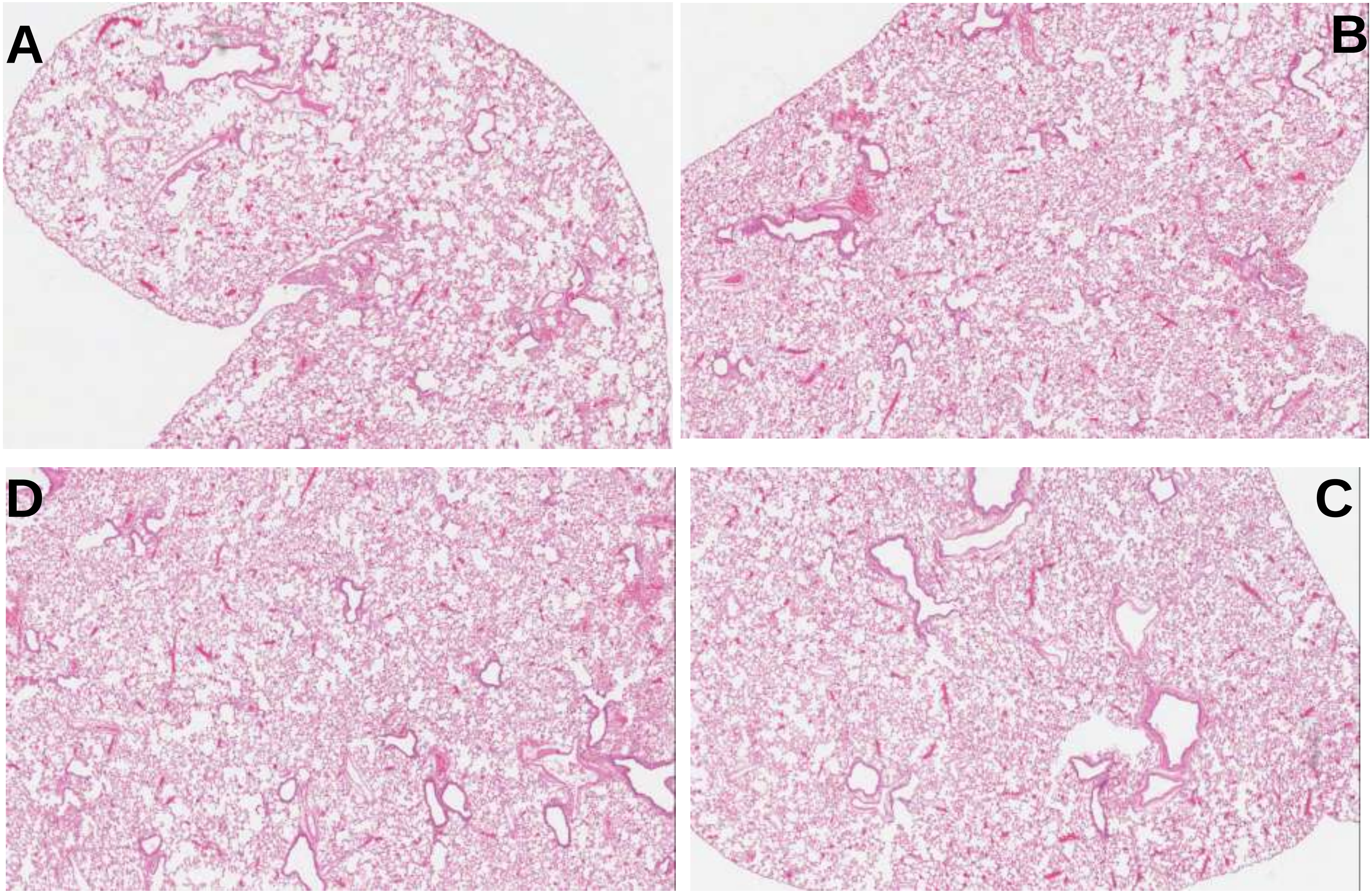

Slide # 2

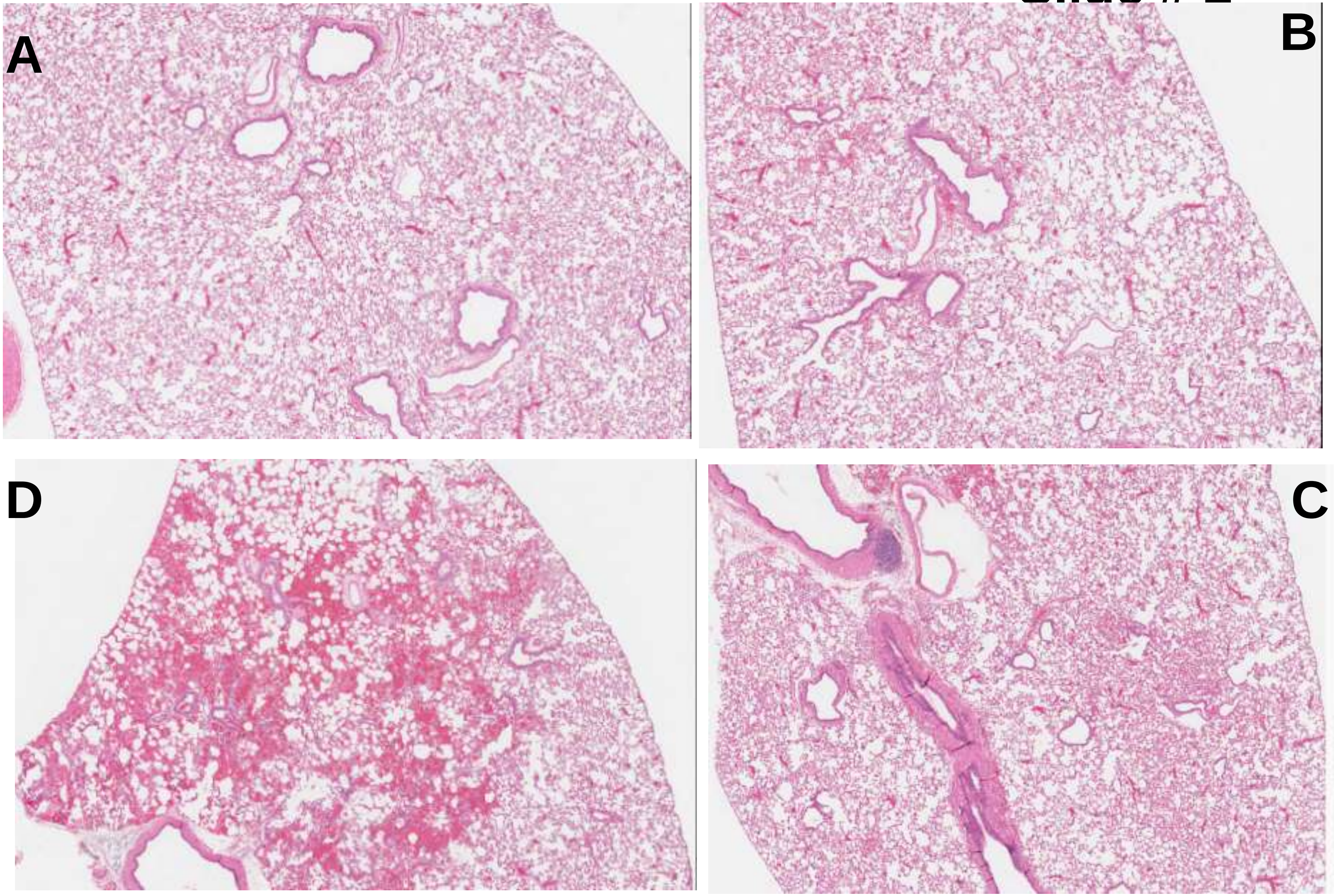

Slide # 2

A

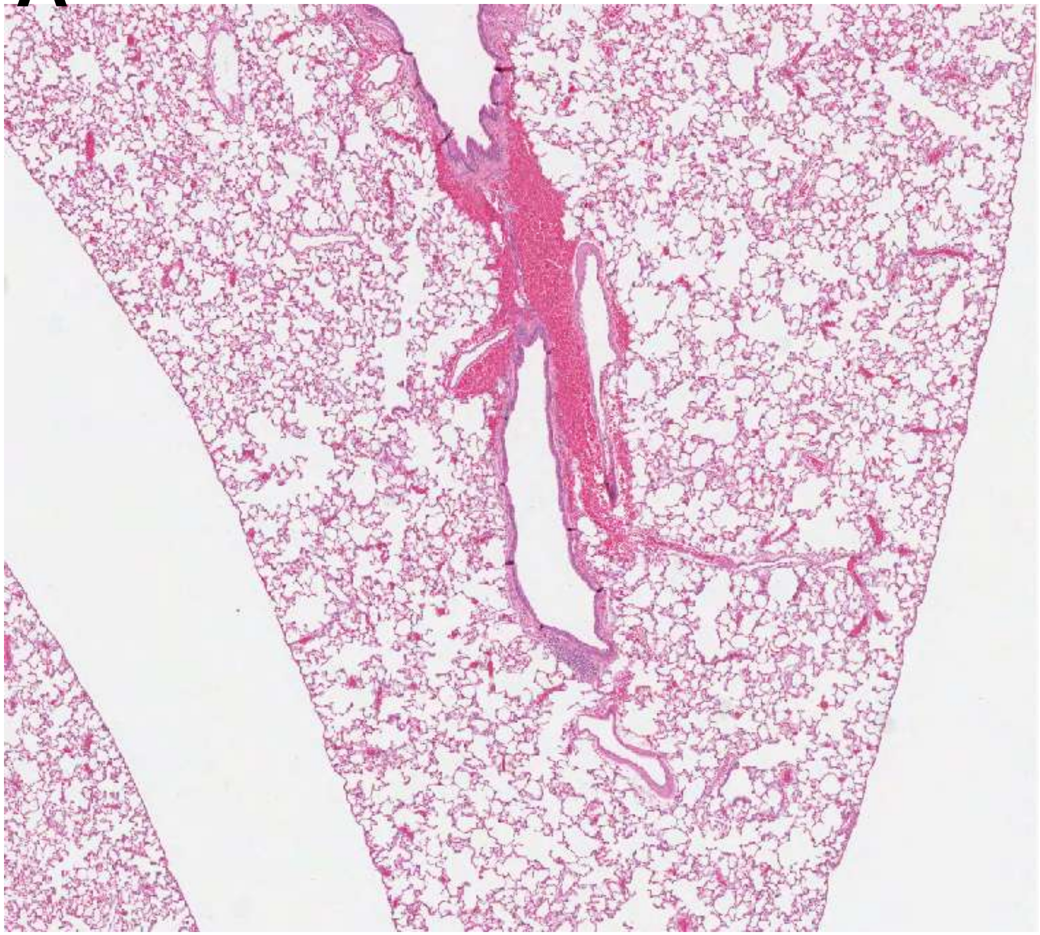

B

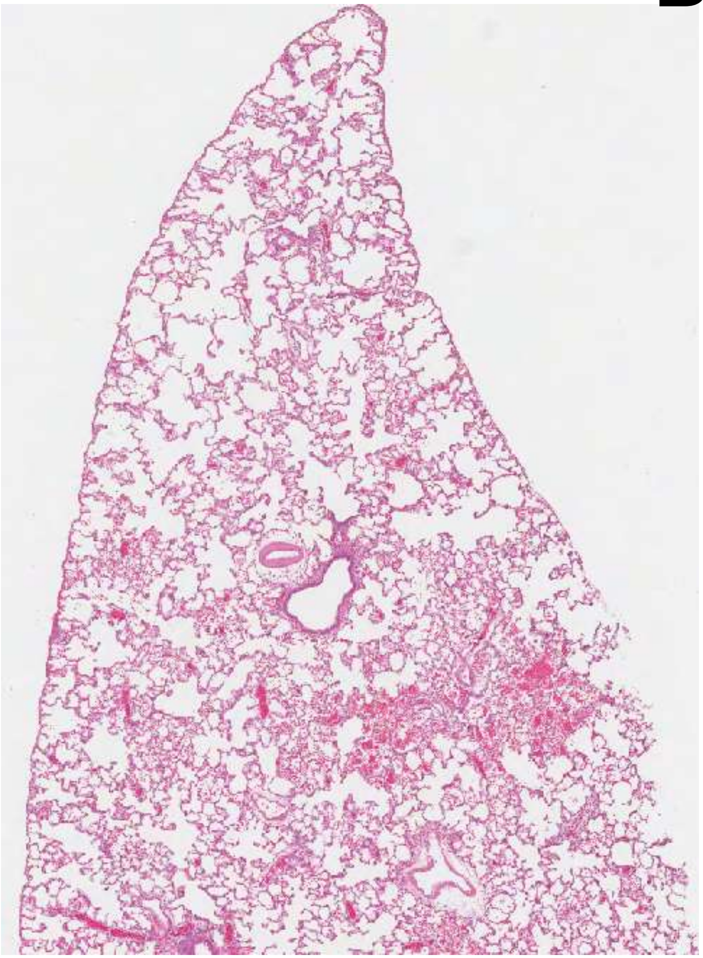

Slide # 3

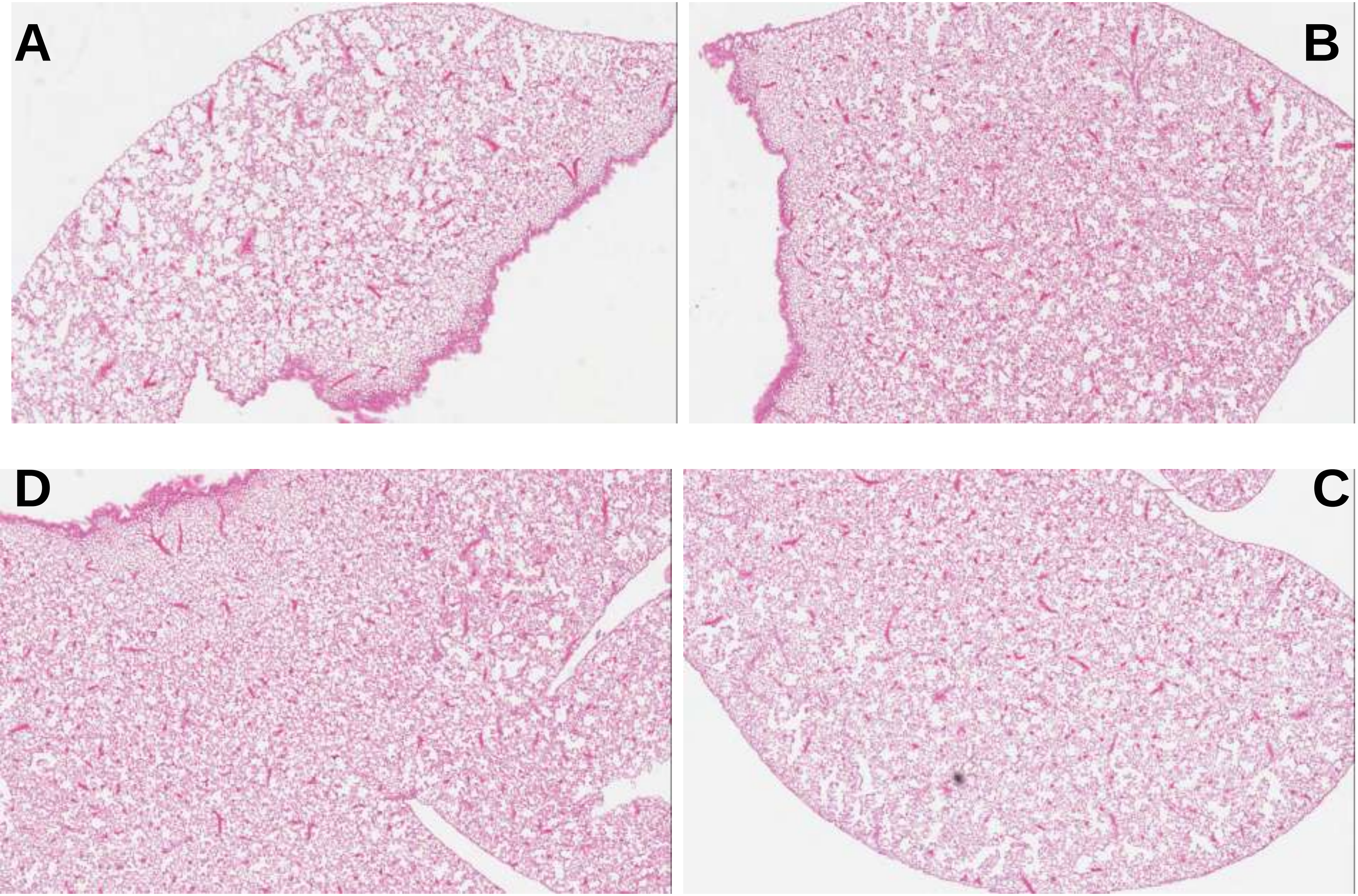

Slide # 3

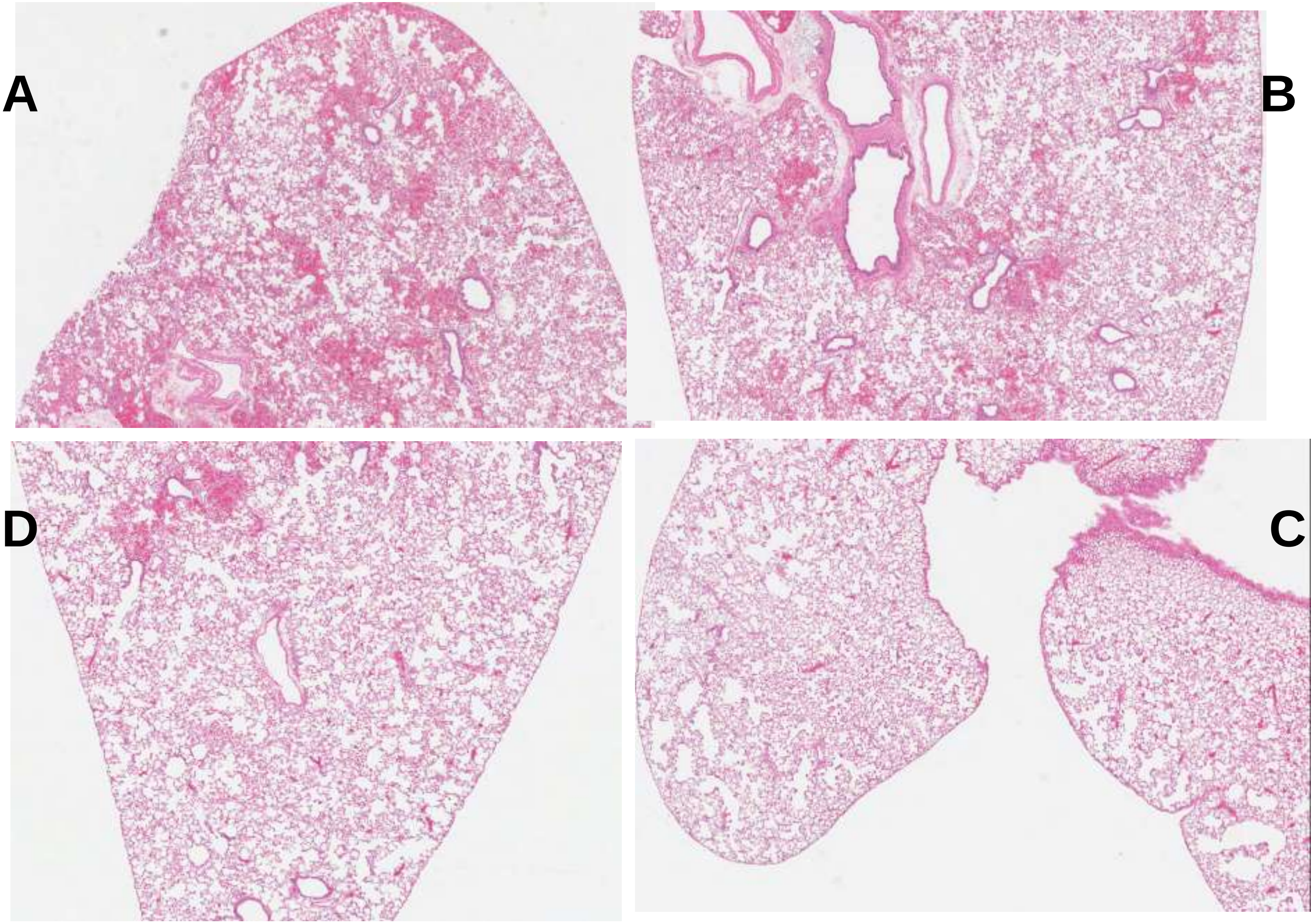

Slide # 4

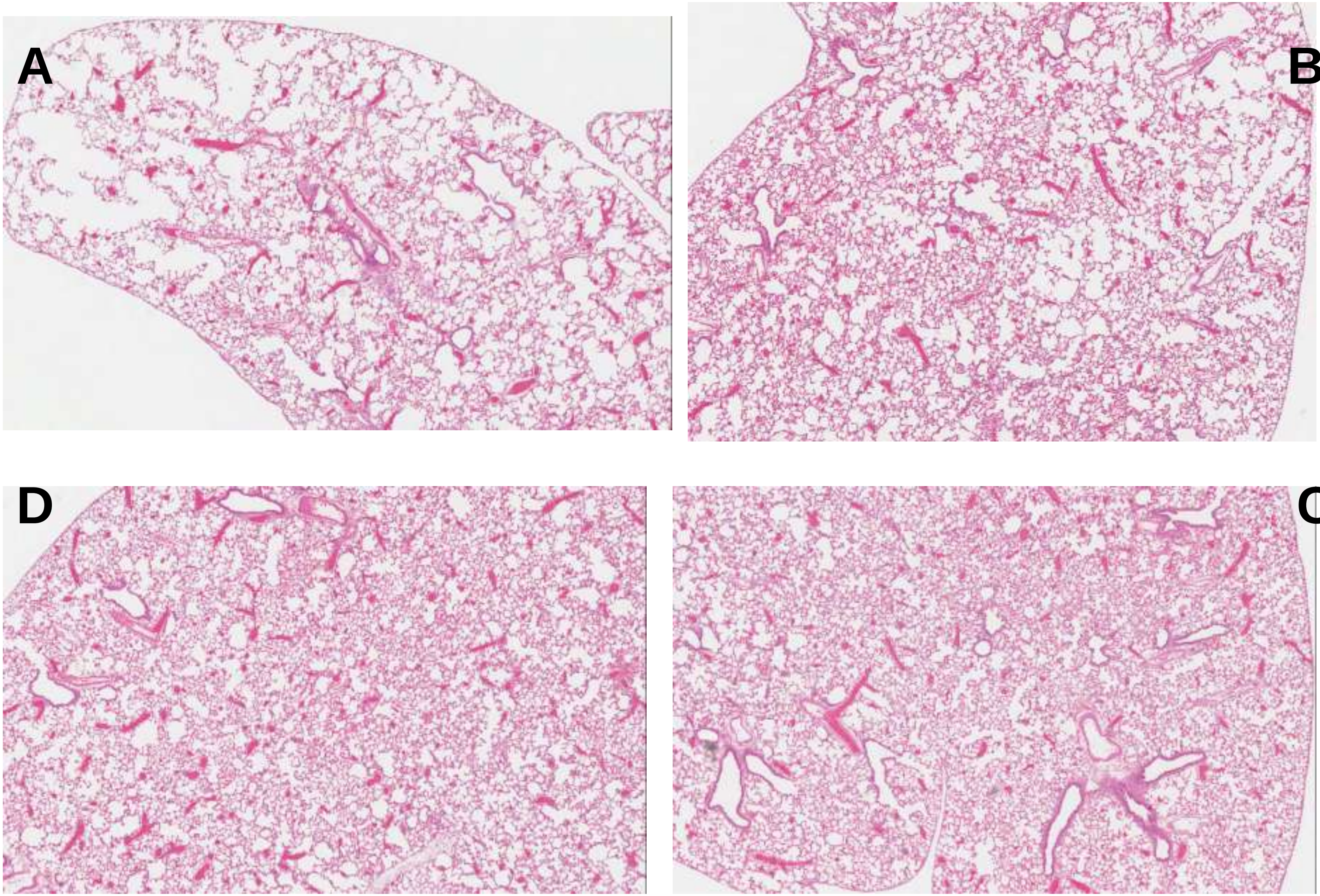

Slide # 4

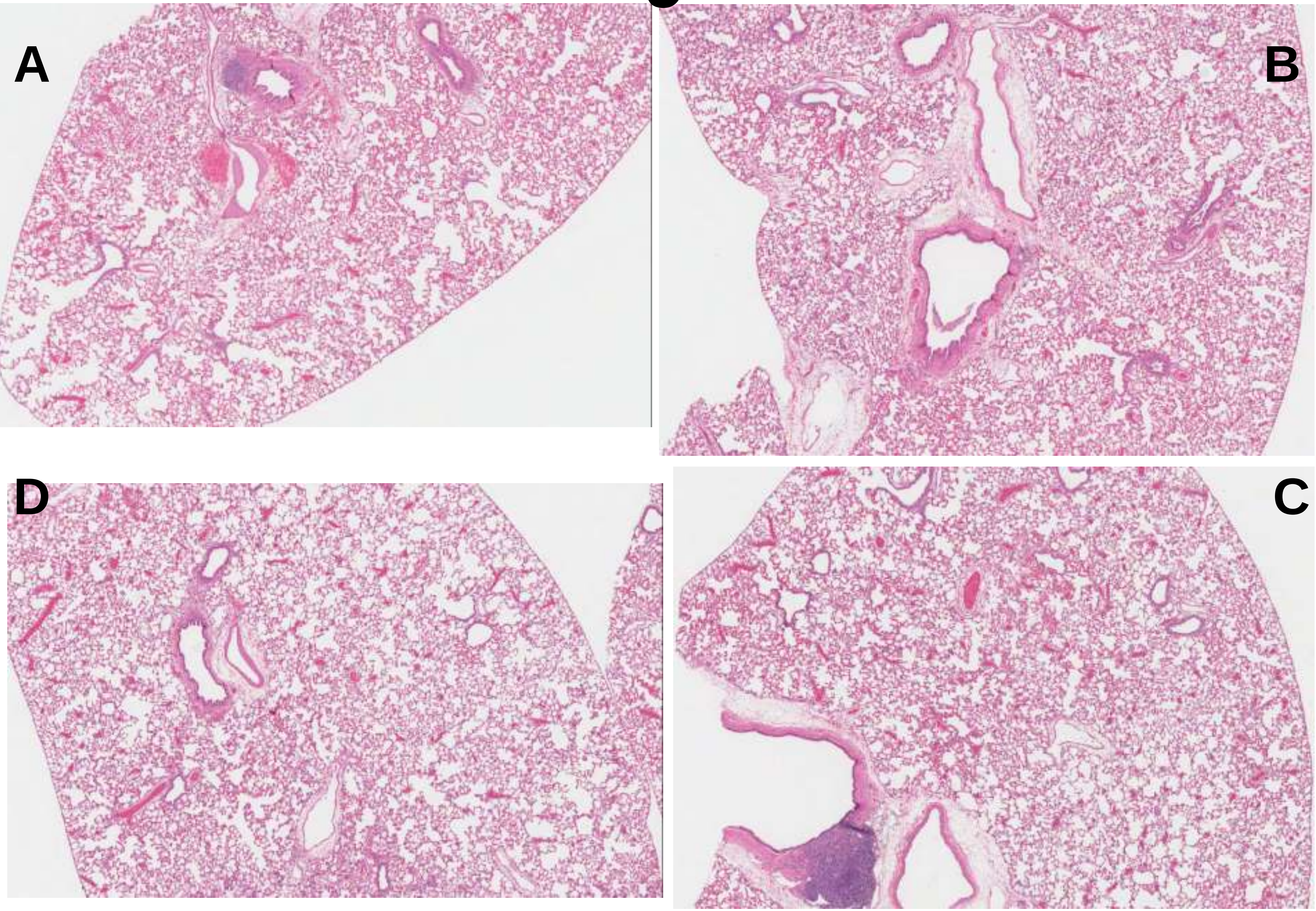

Slide # 5

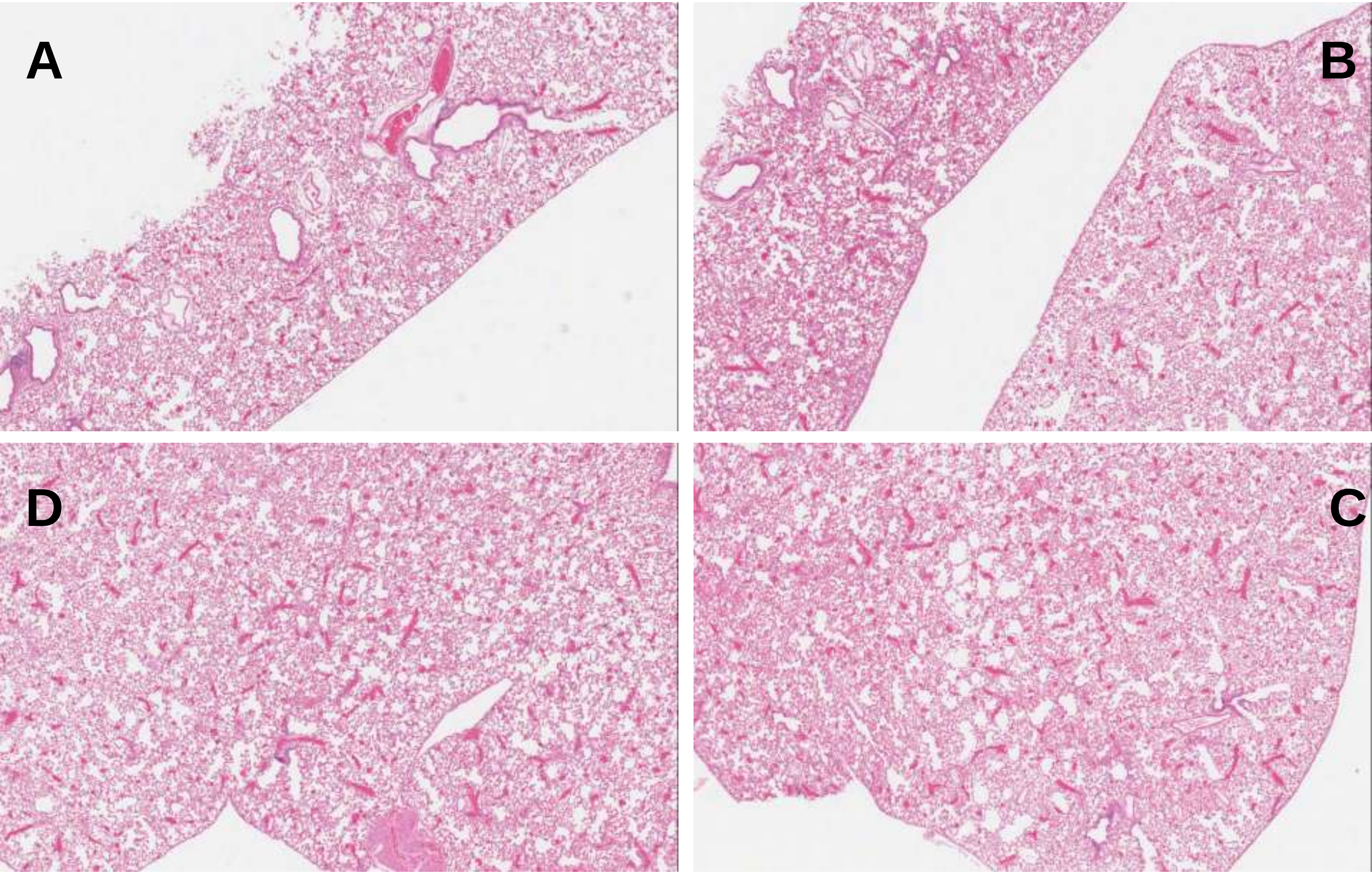

Slide # 5

A

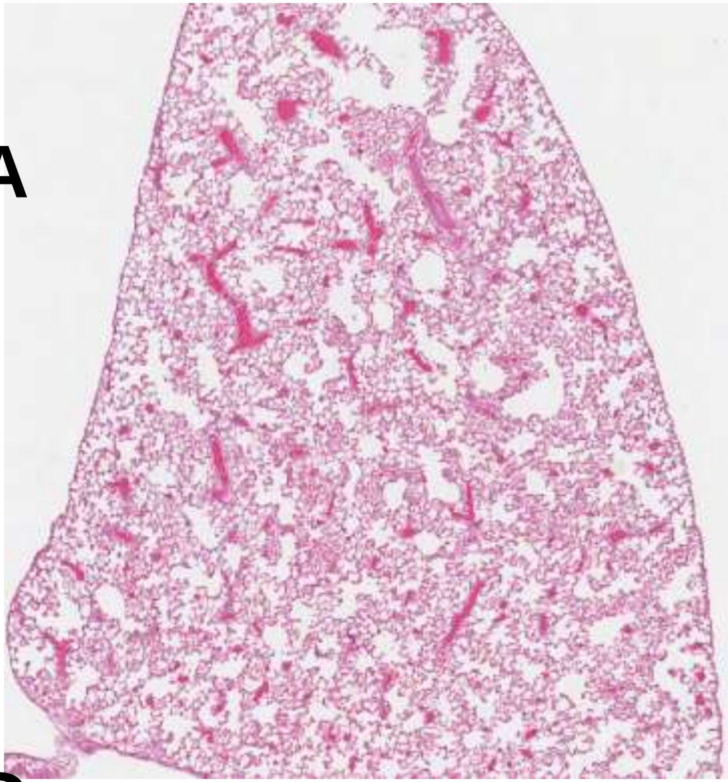

B

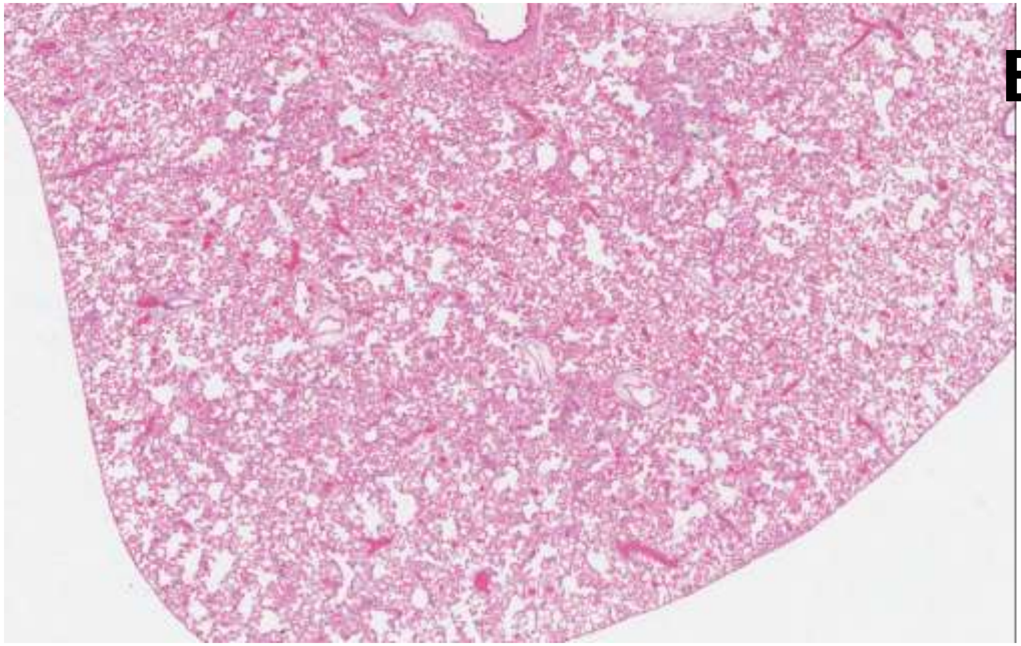

D

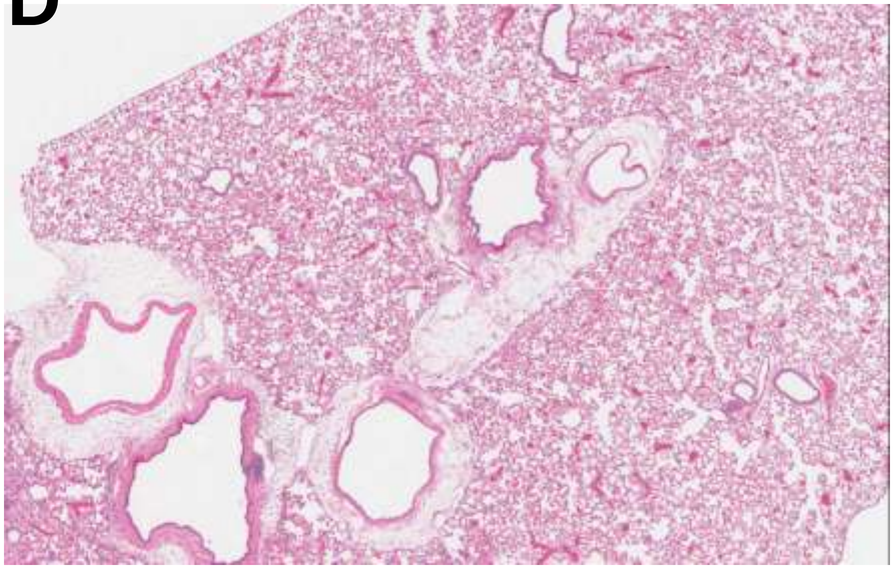

C

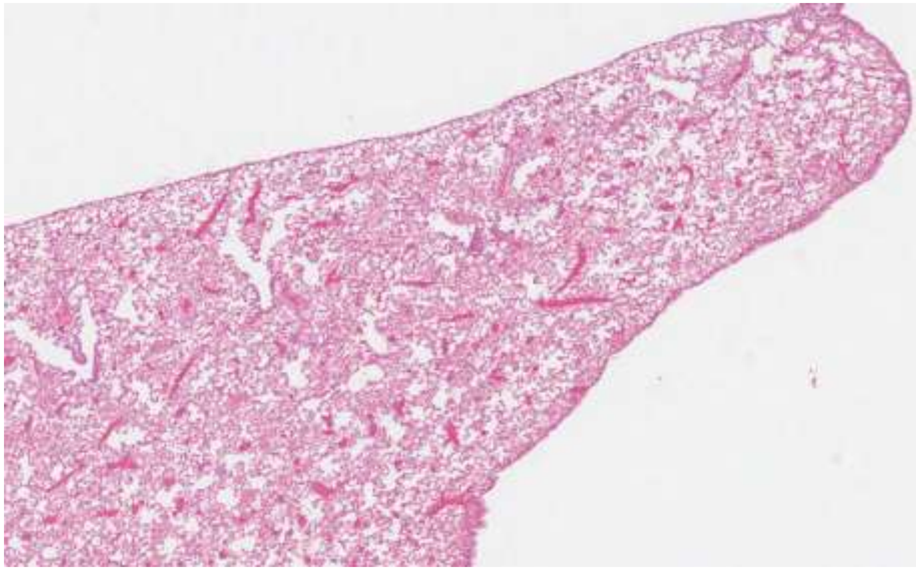

Slide # 7

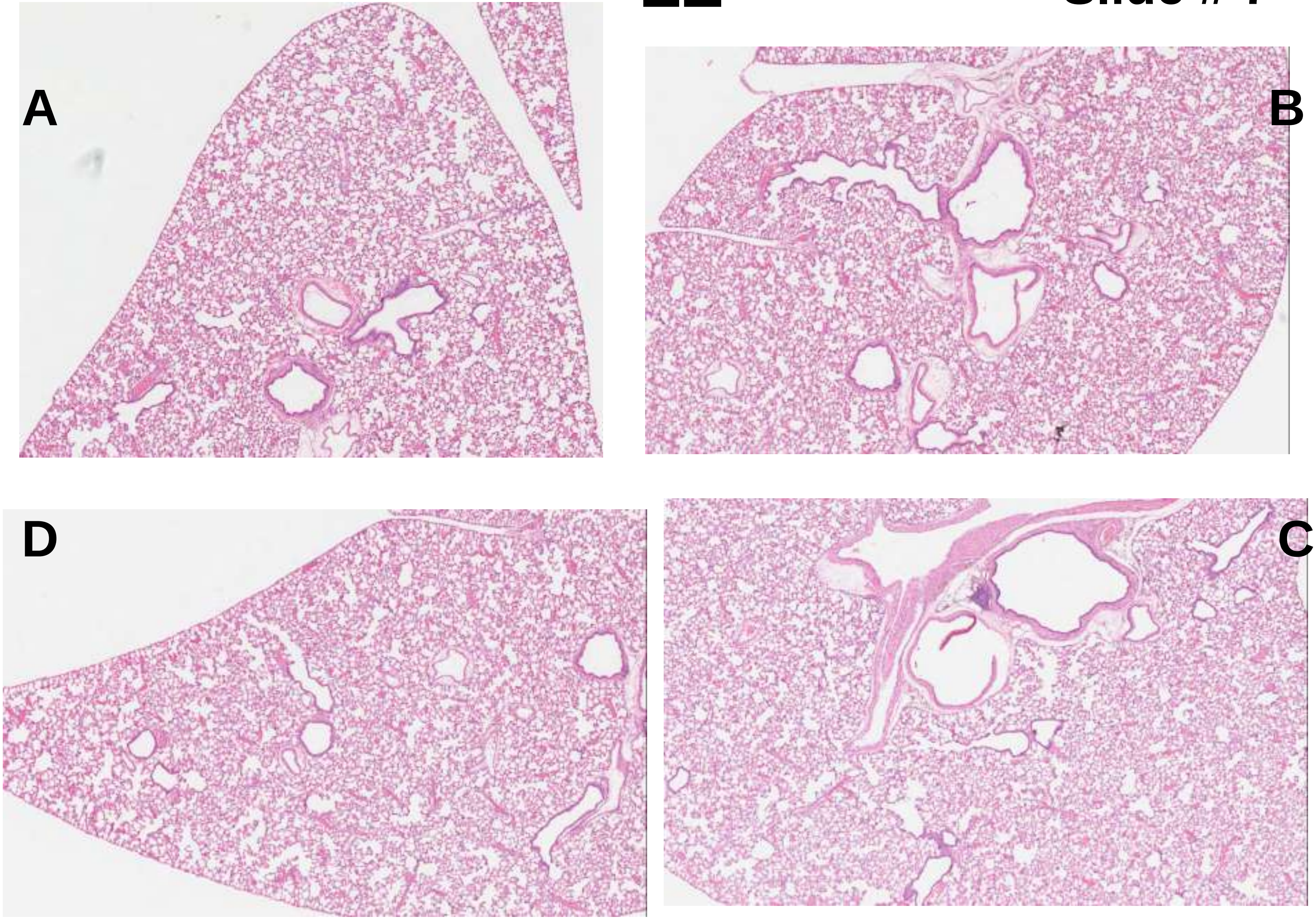

Slide # 7

A

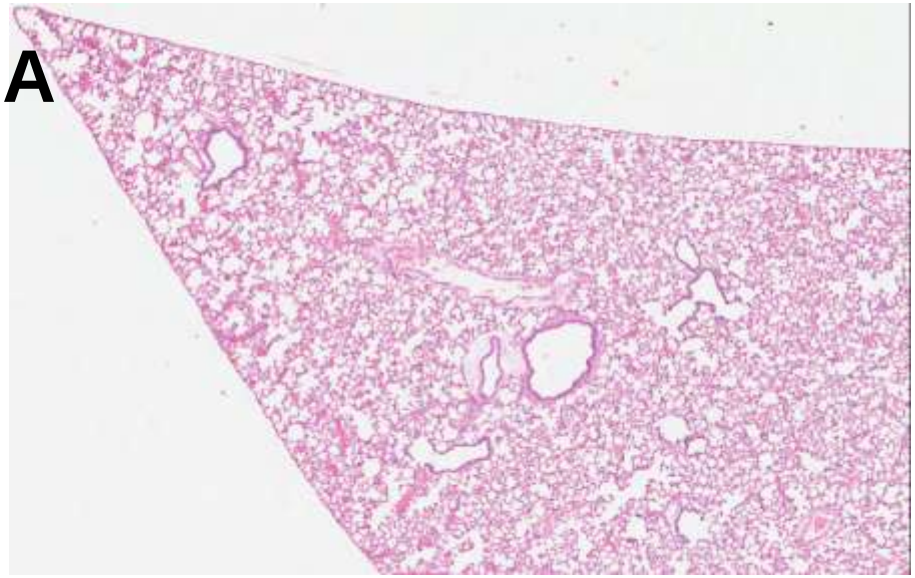

B

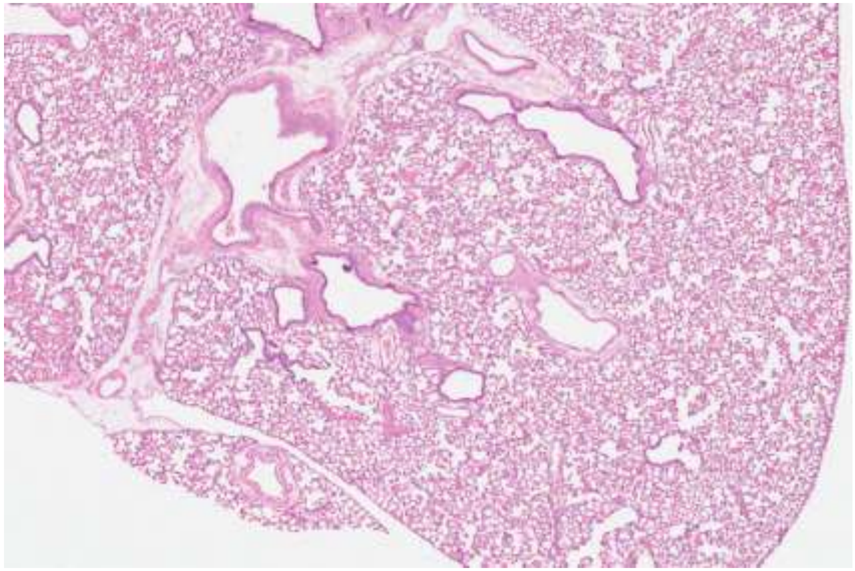

D

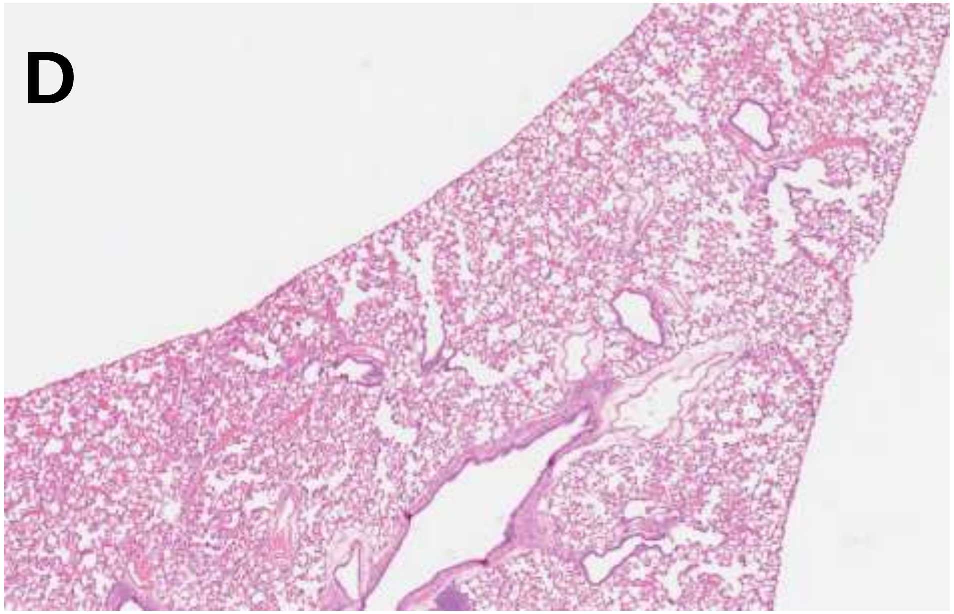

C

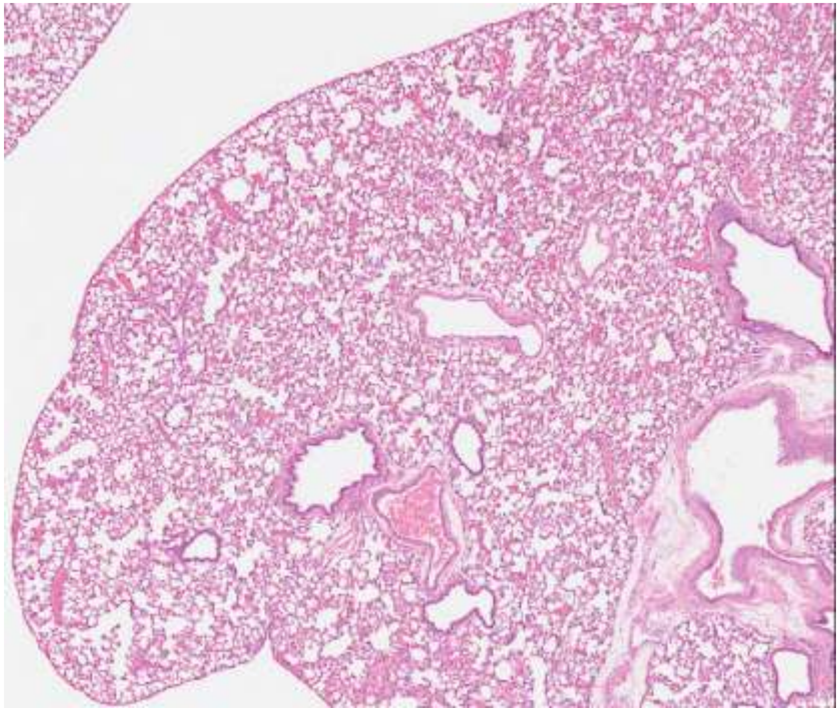

Slide # 9

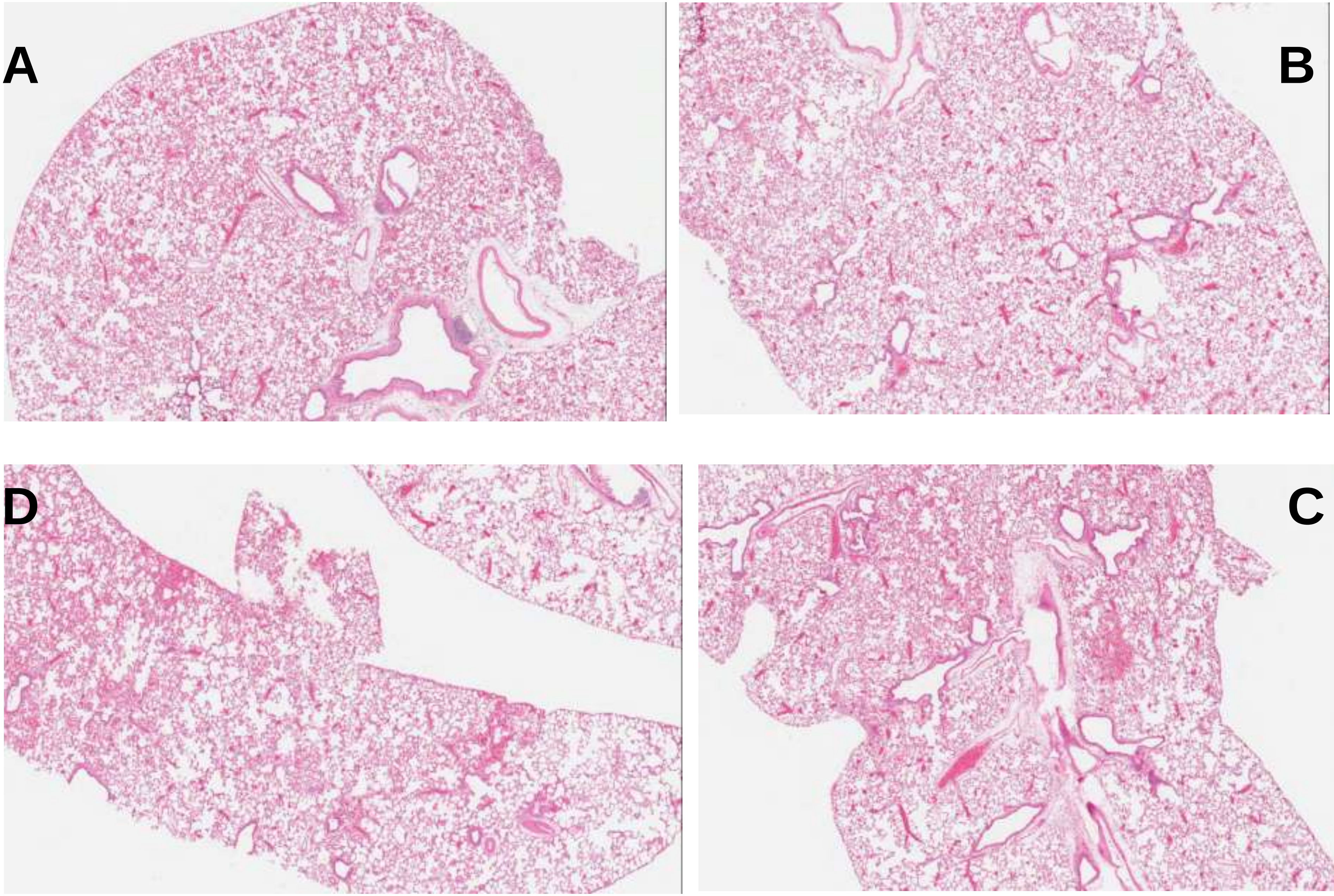

Slide # 9

15

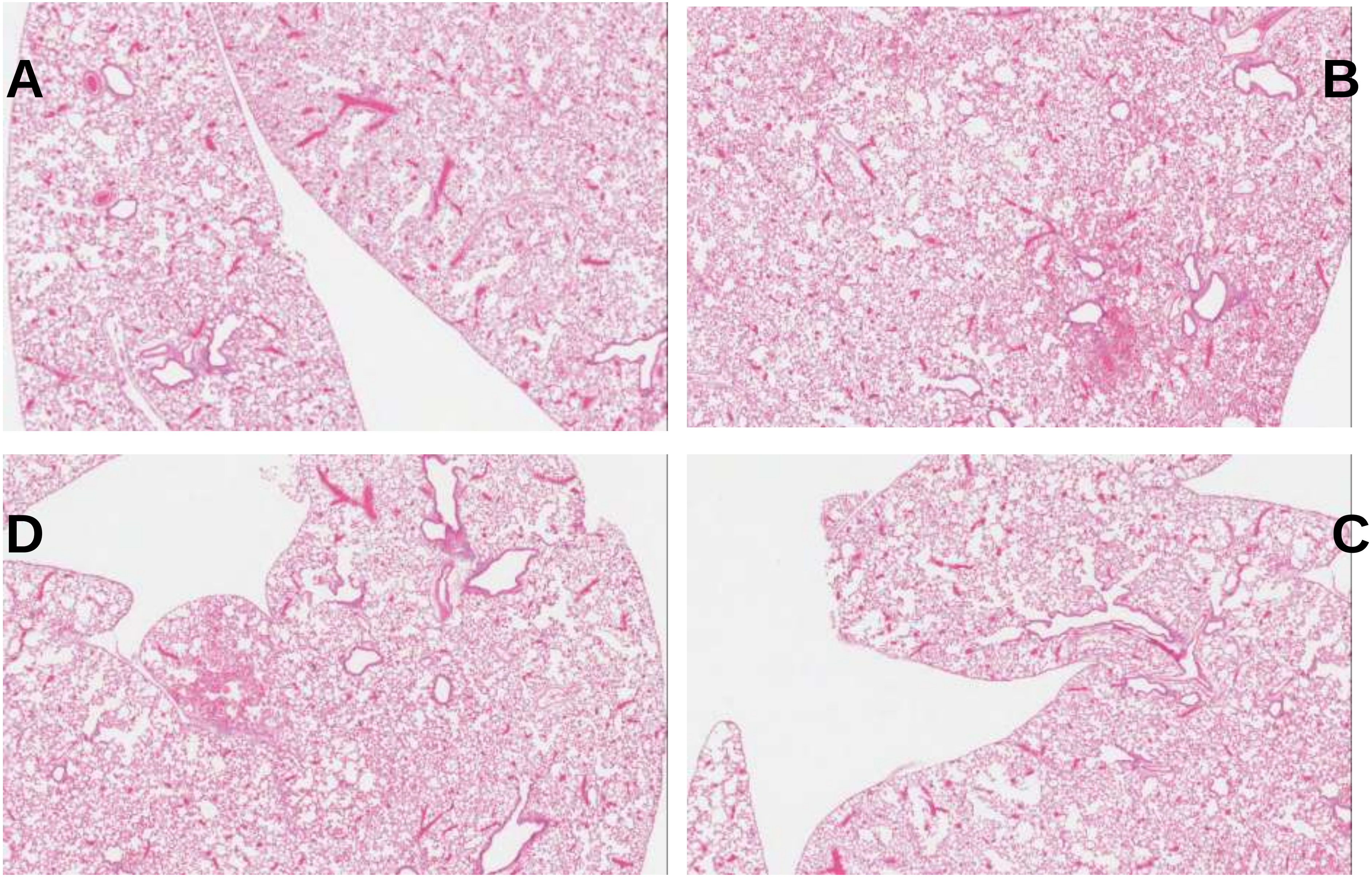

Slide # 11

16

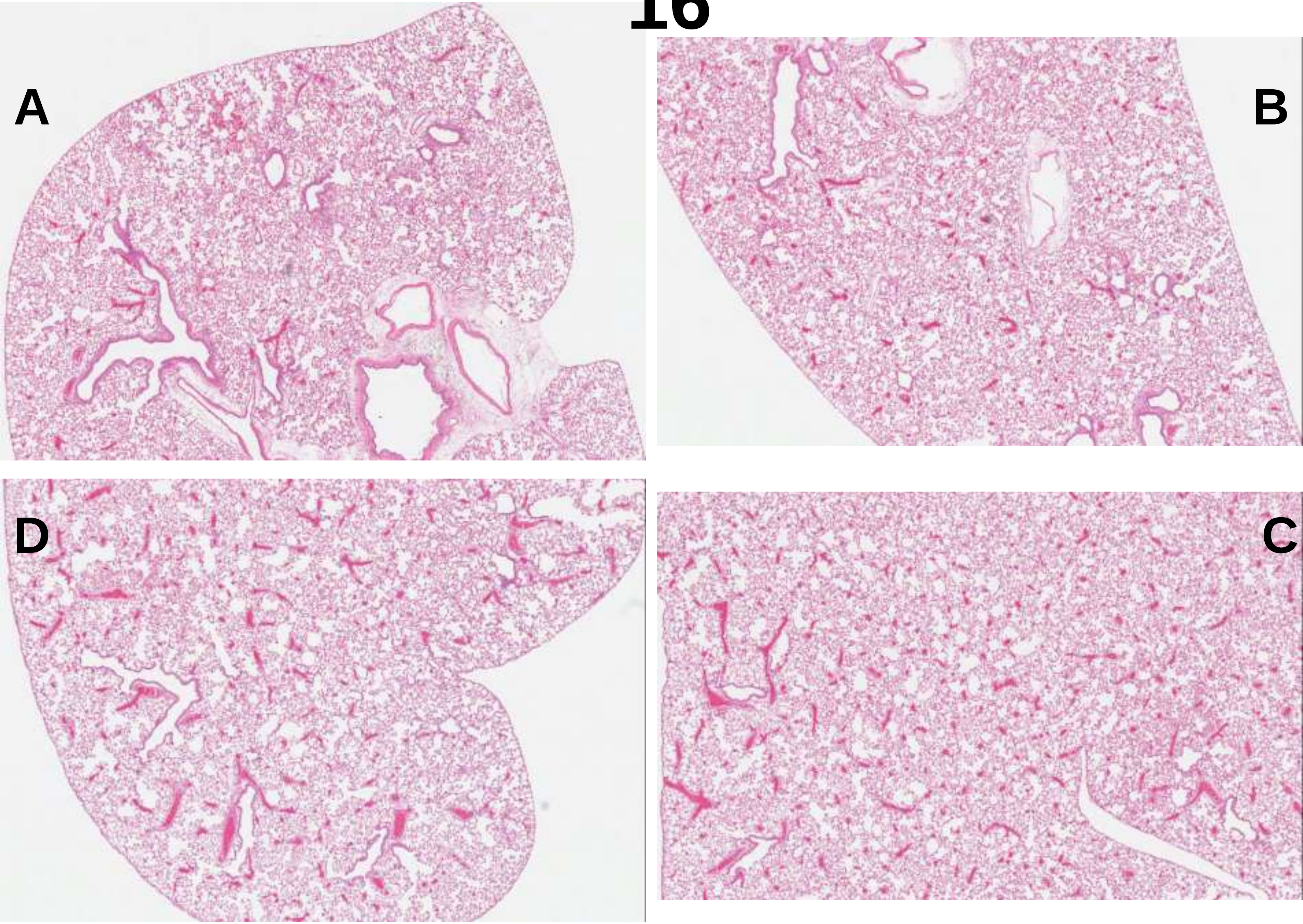

Slide # 11

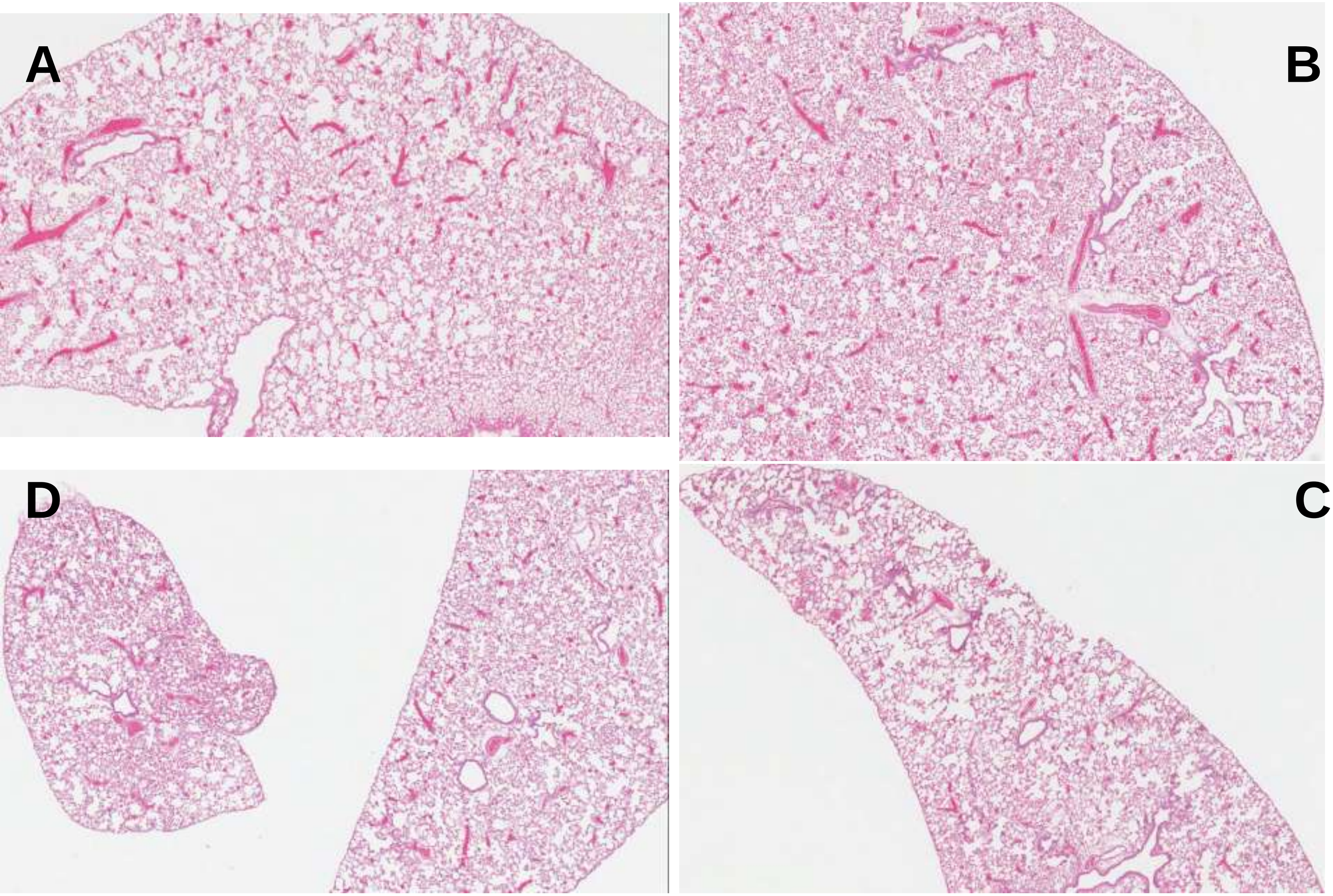

Slide # 11

A

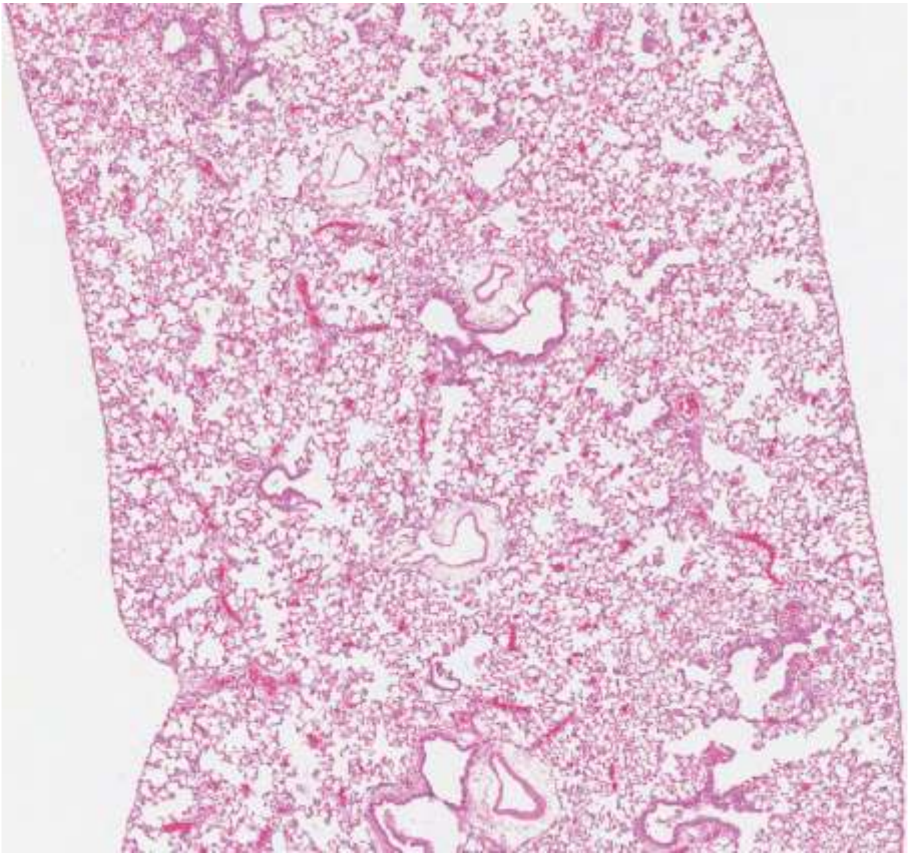

B

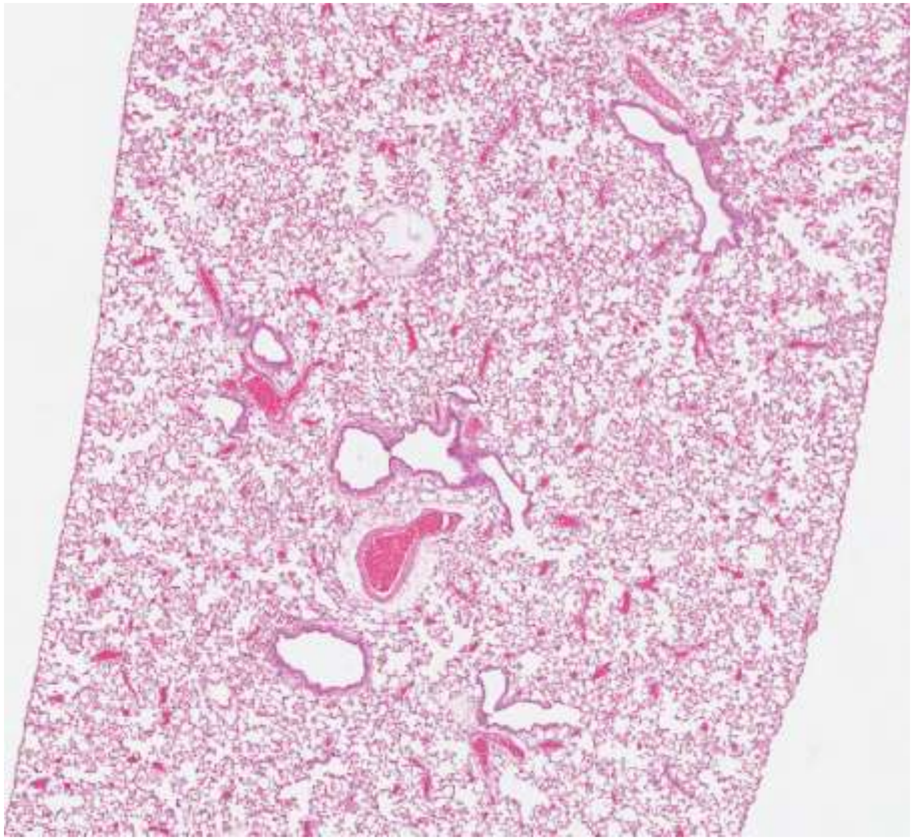

Slide # 12

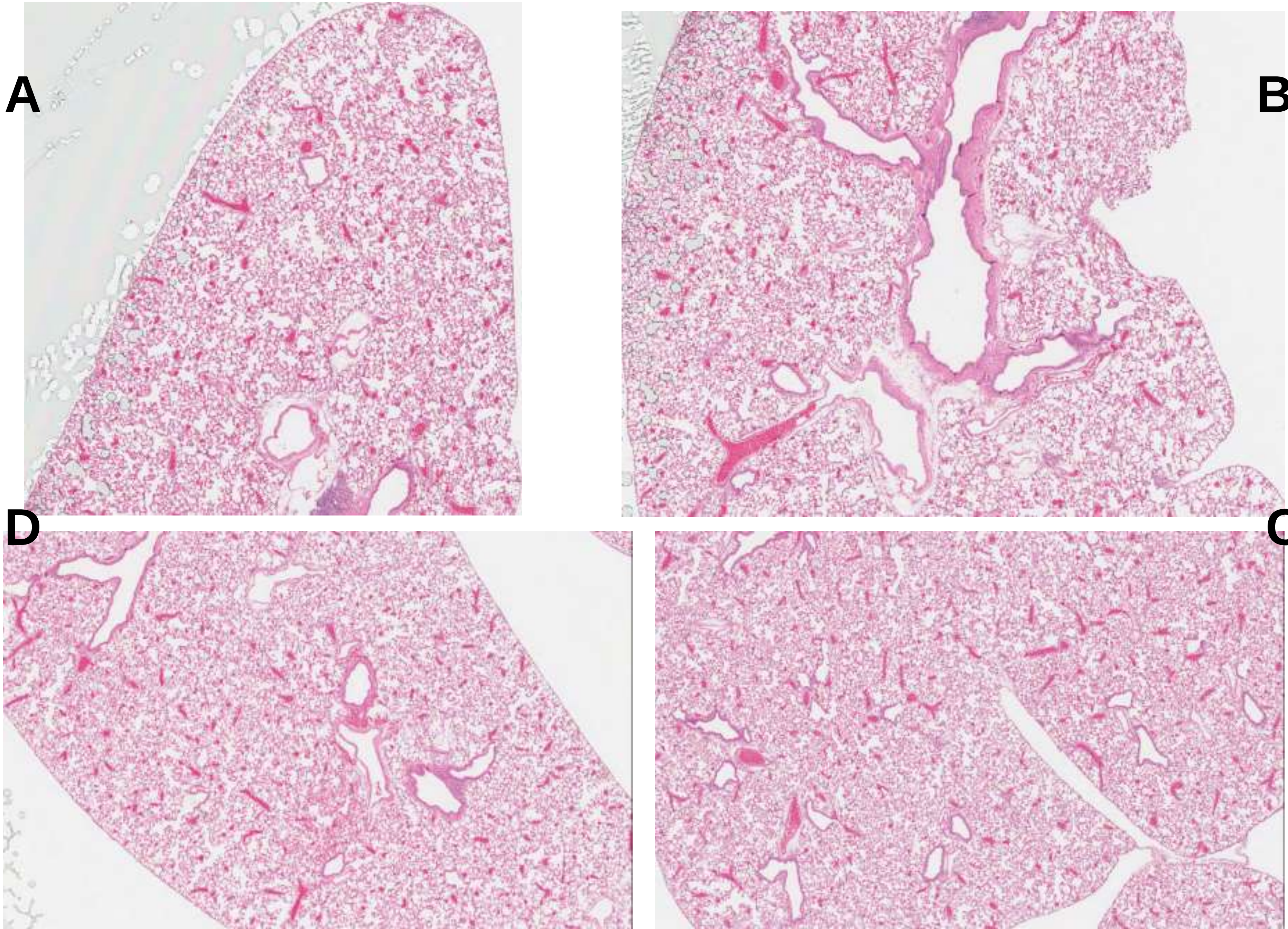

A B D C

Slide # 12

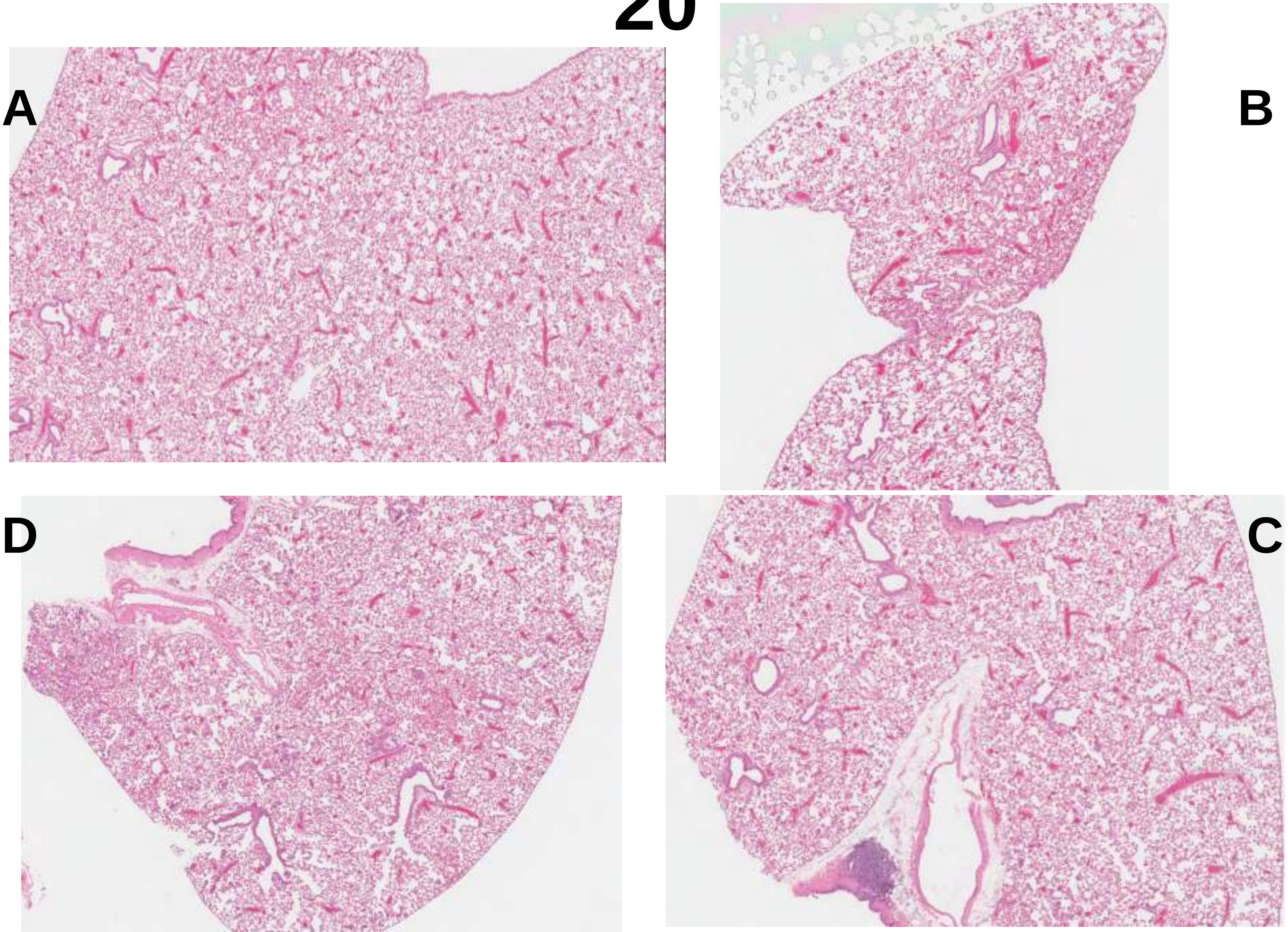

Slide # 12

A

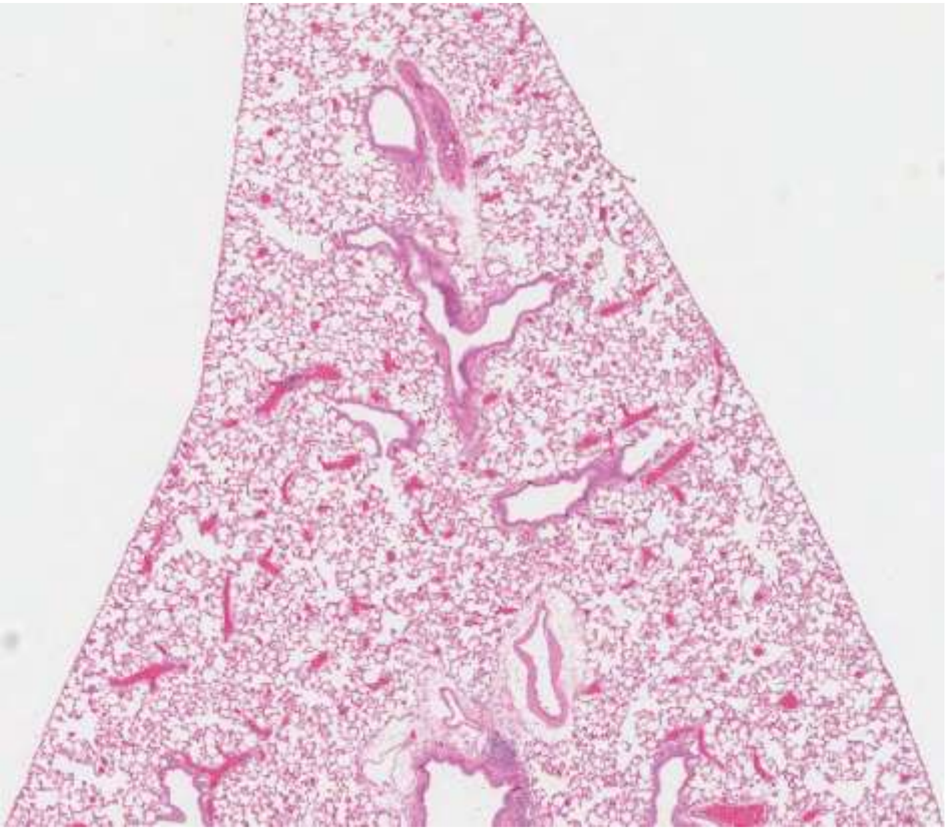

B

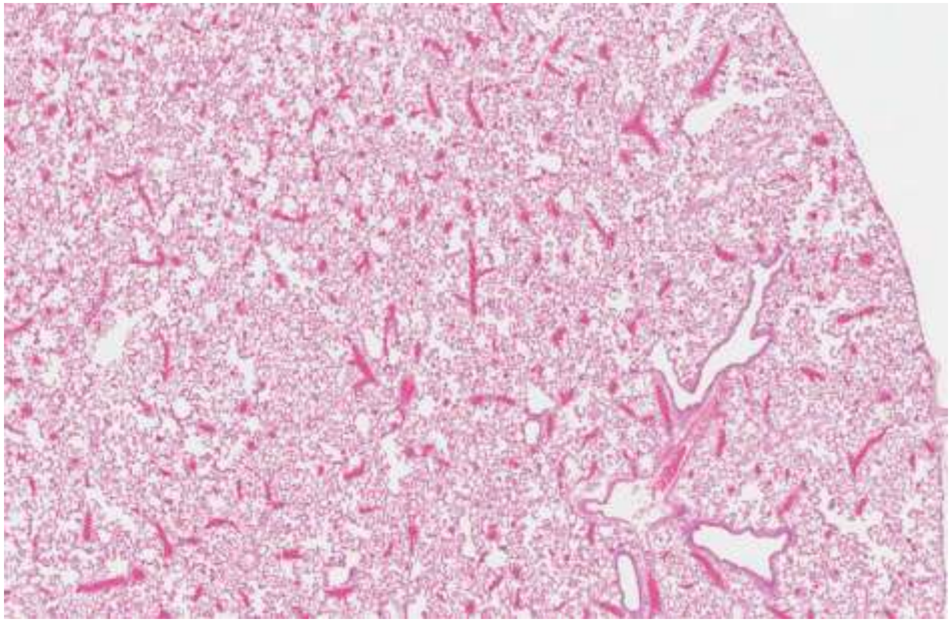

Slide # 14

A

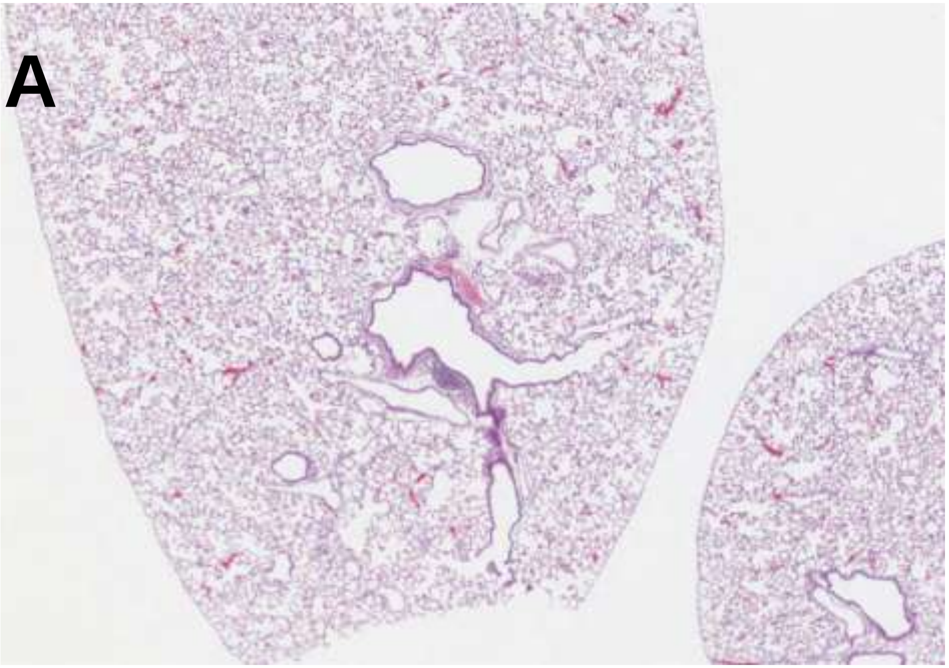

B

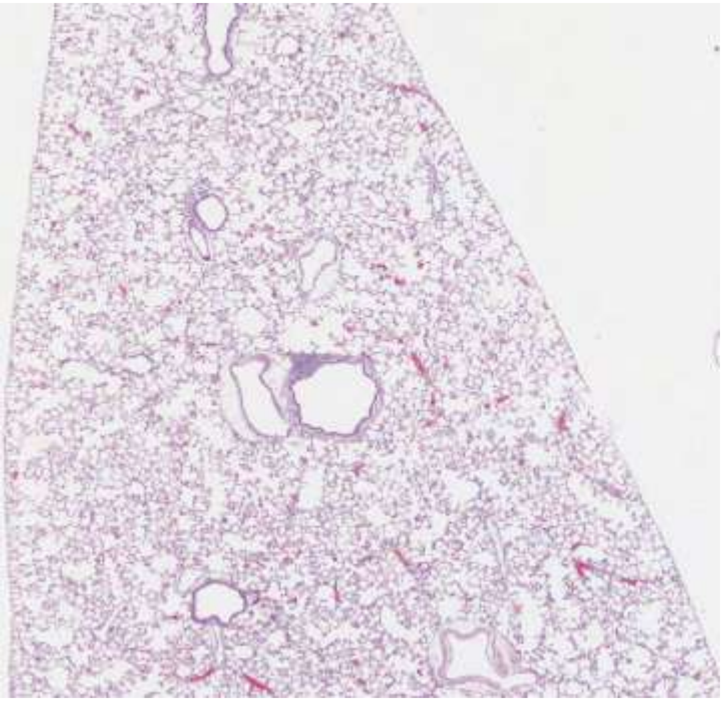

D

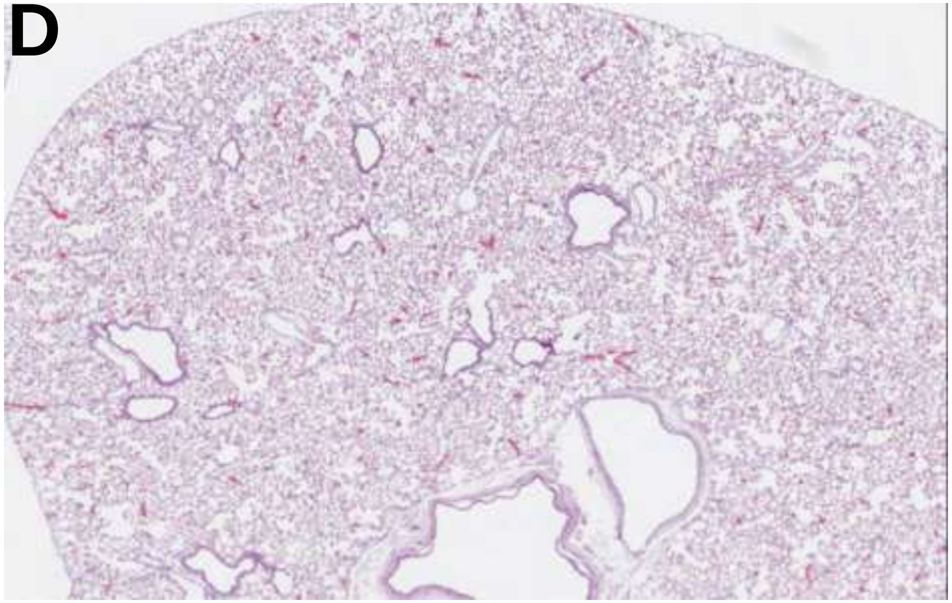

C

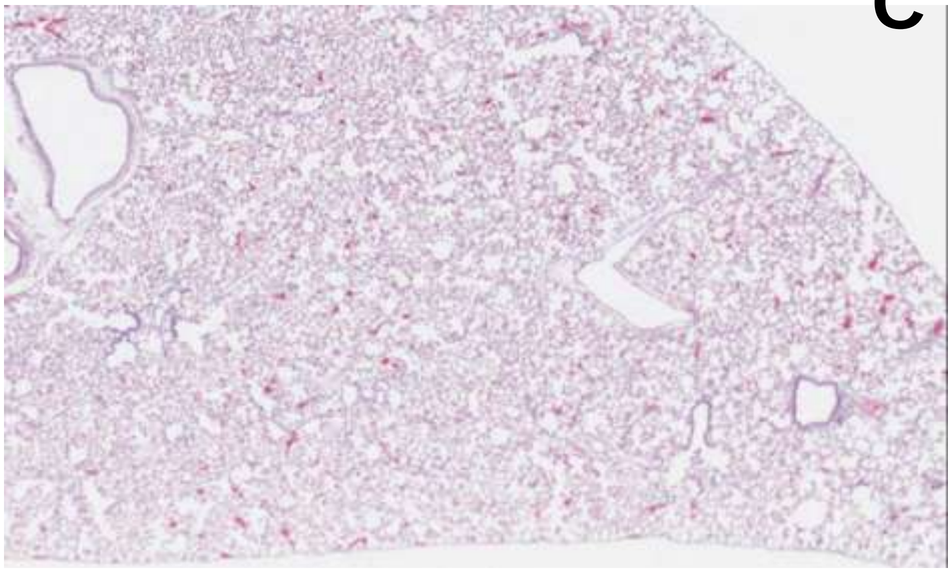

Slide # 14

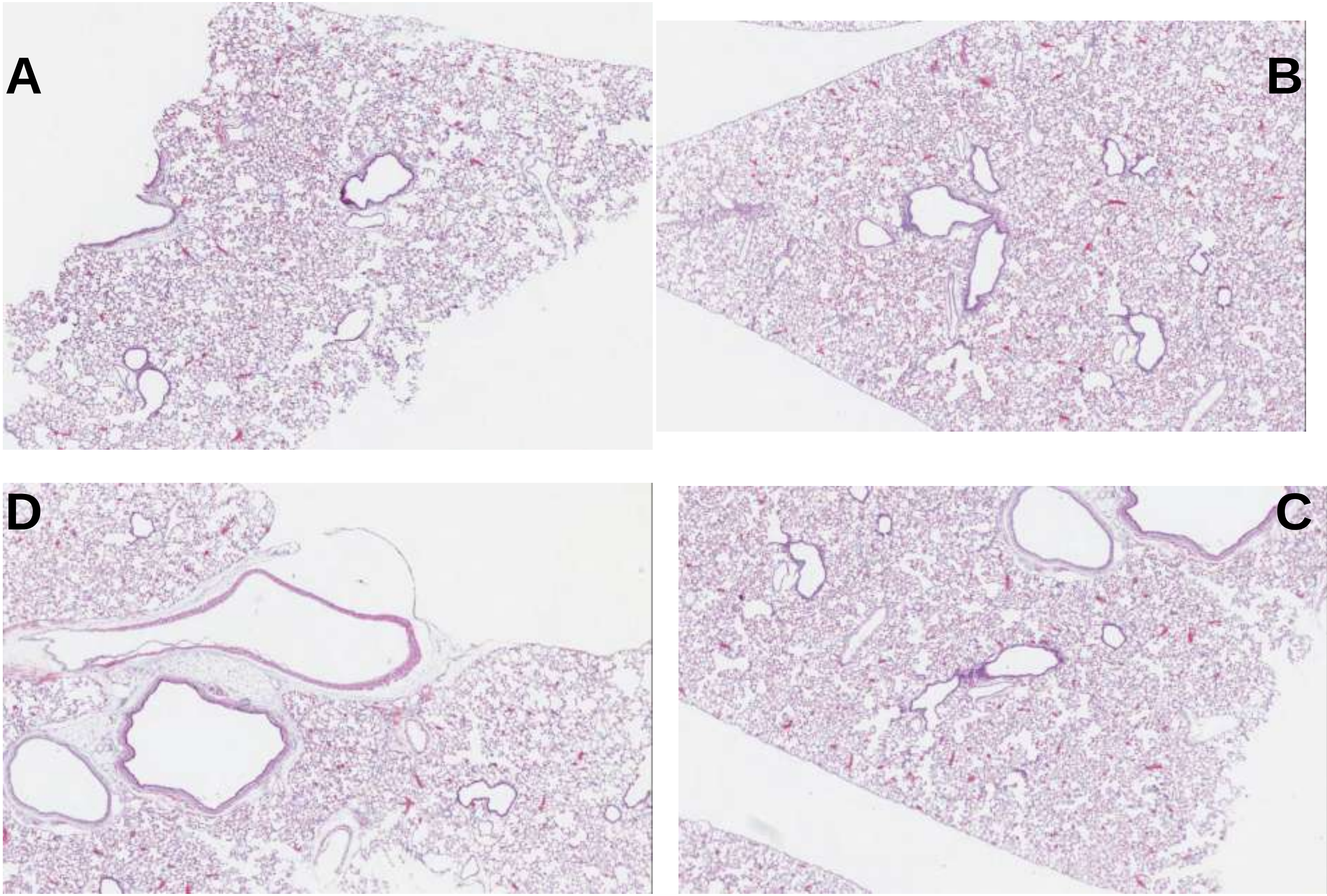

Slide # 15

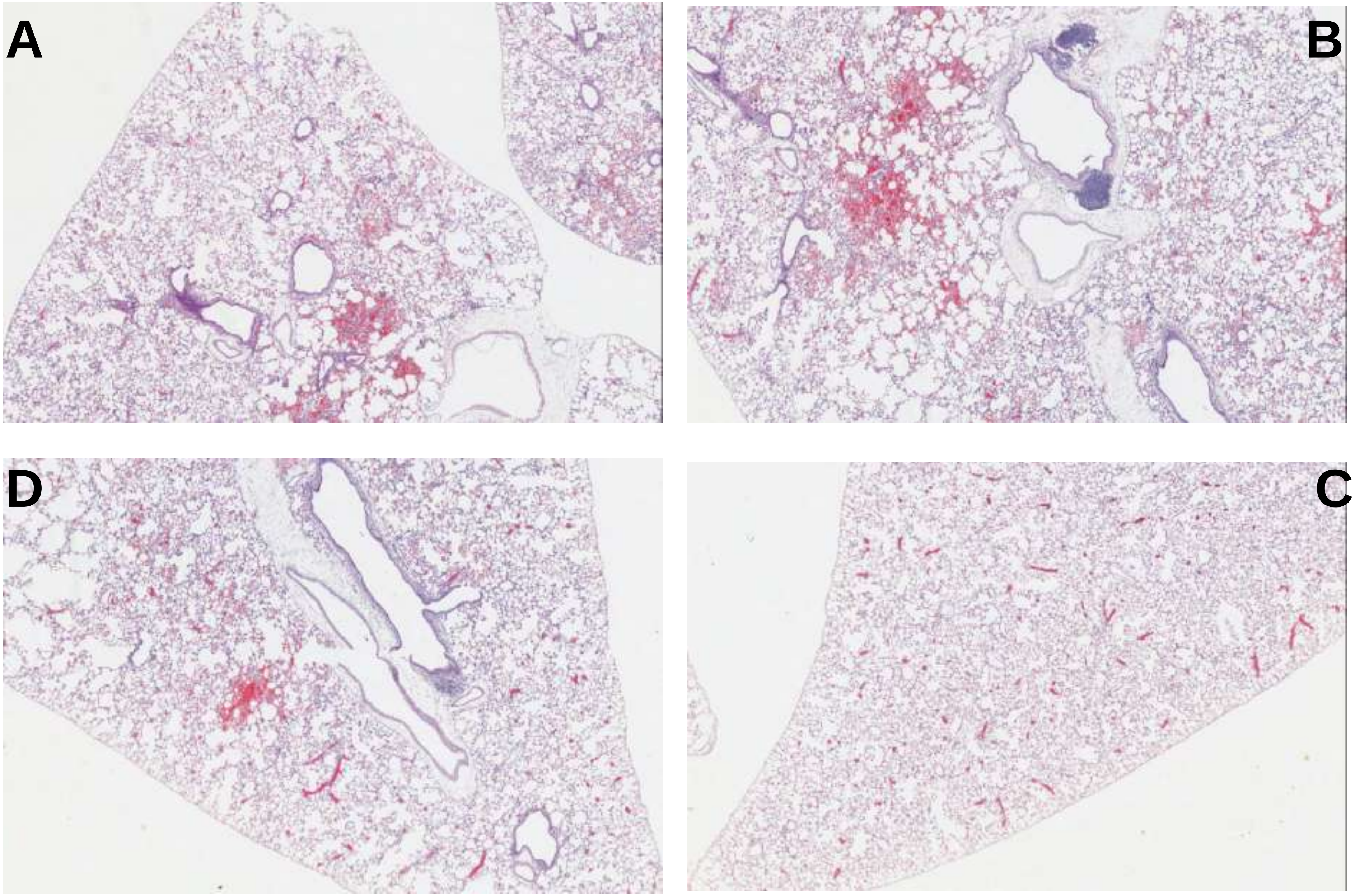

Slide # 15

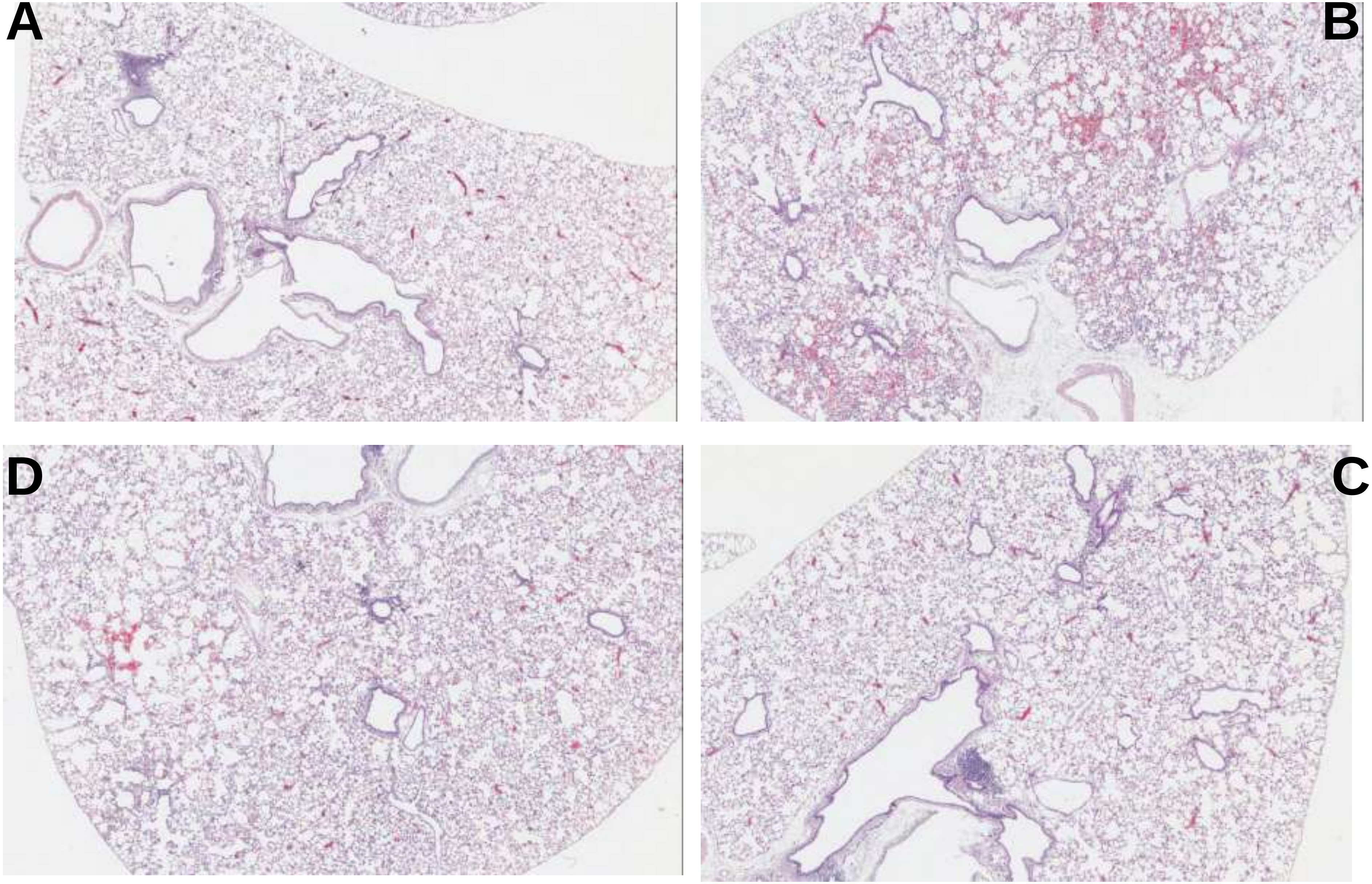

Slide # 16

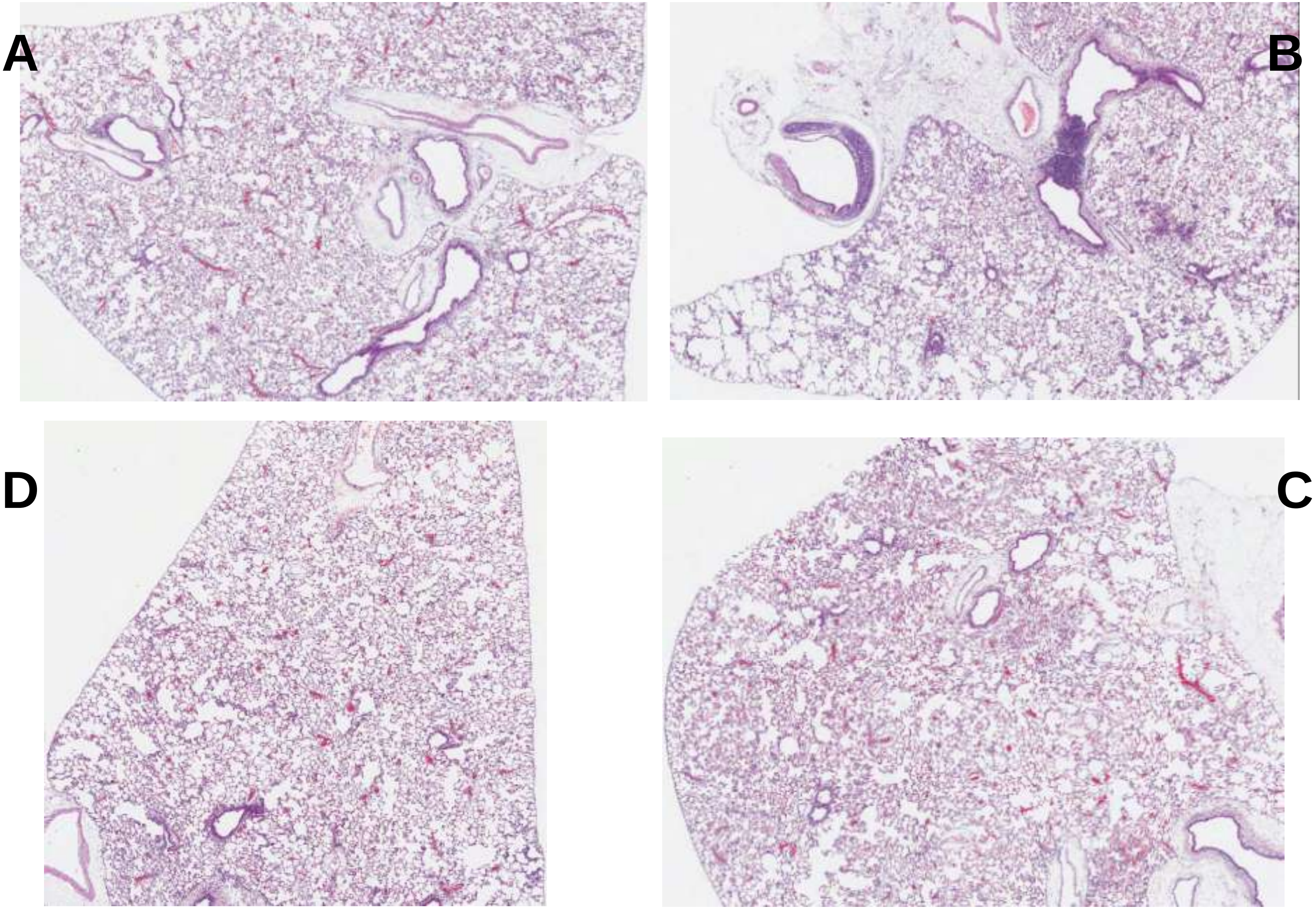

Slide # 16

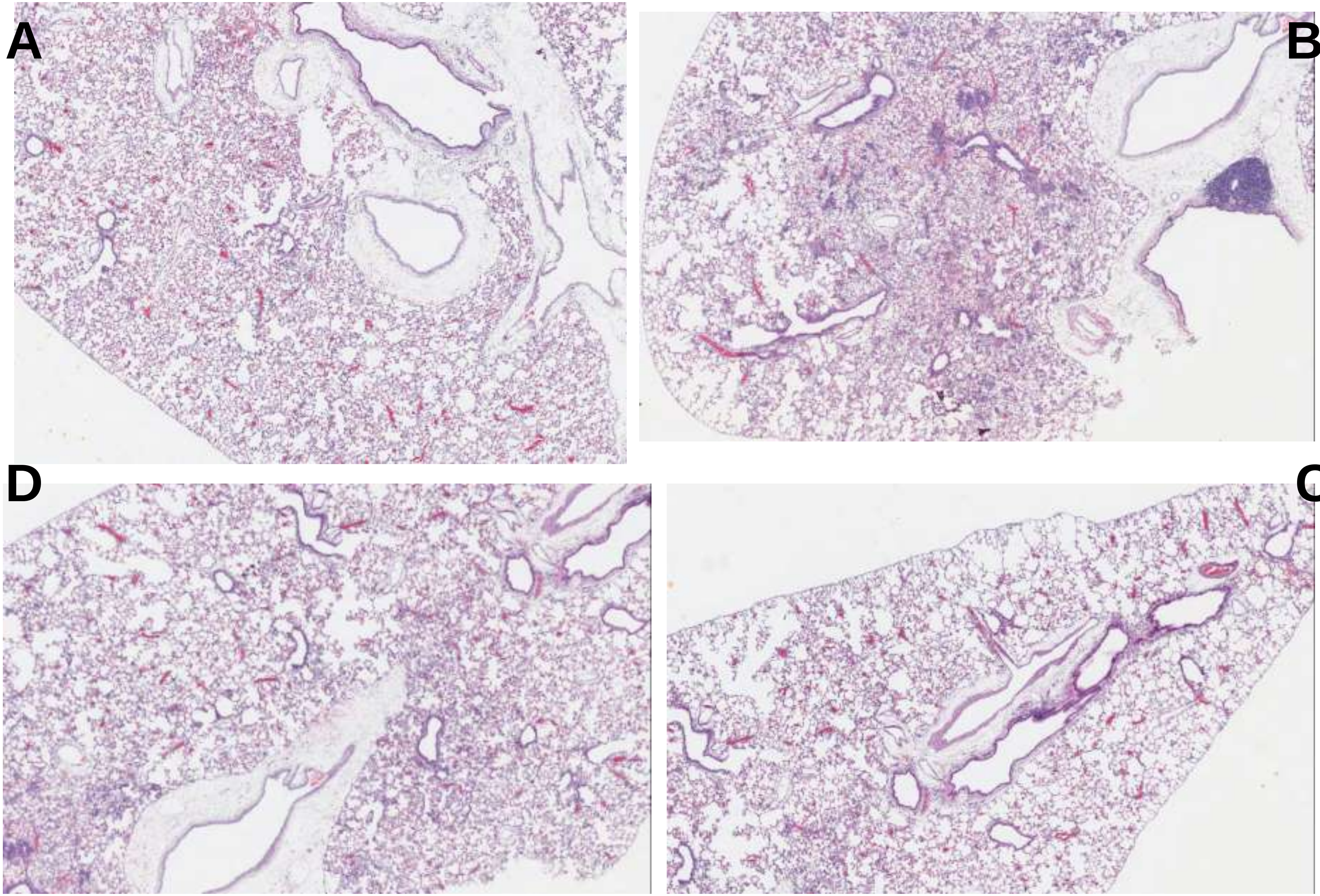

28

Slide # 17

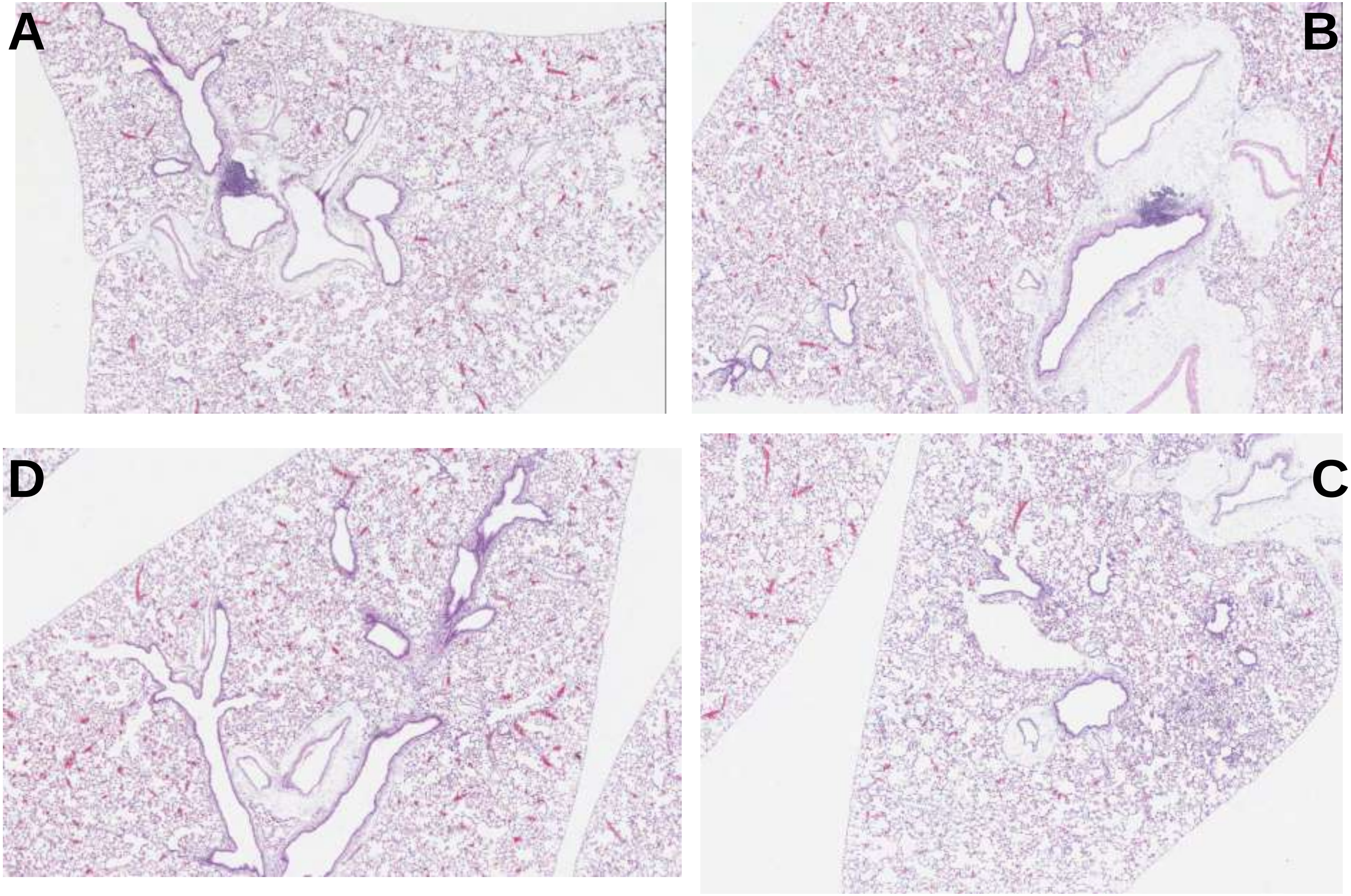

Slide # 17

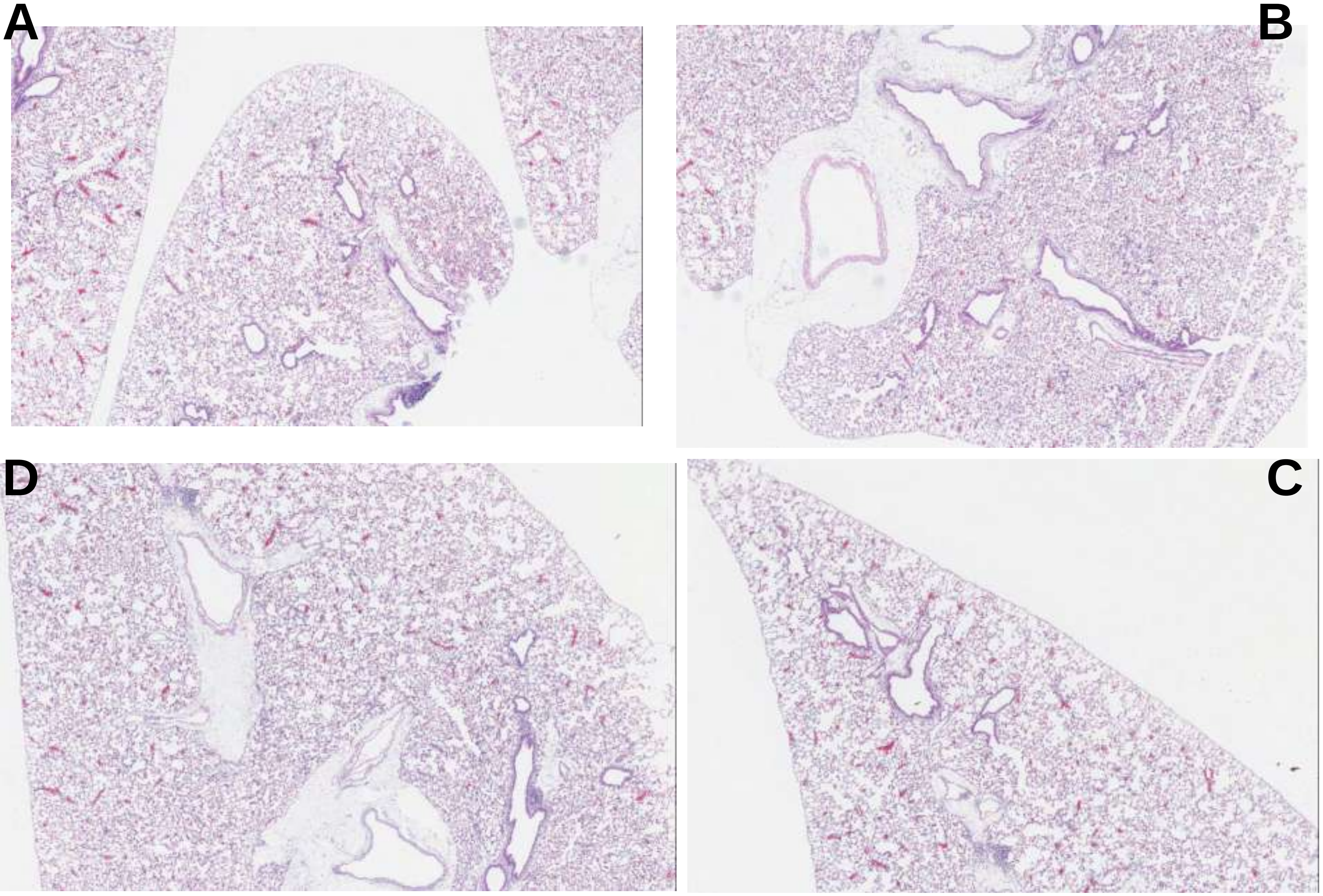

Slide # 18

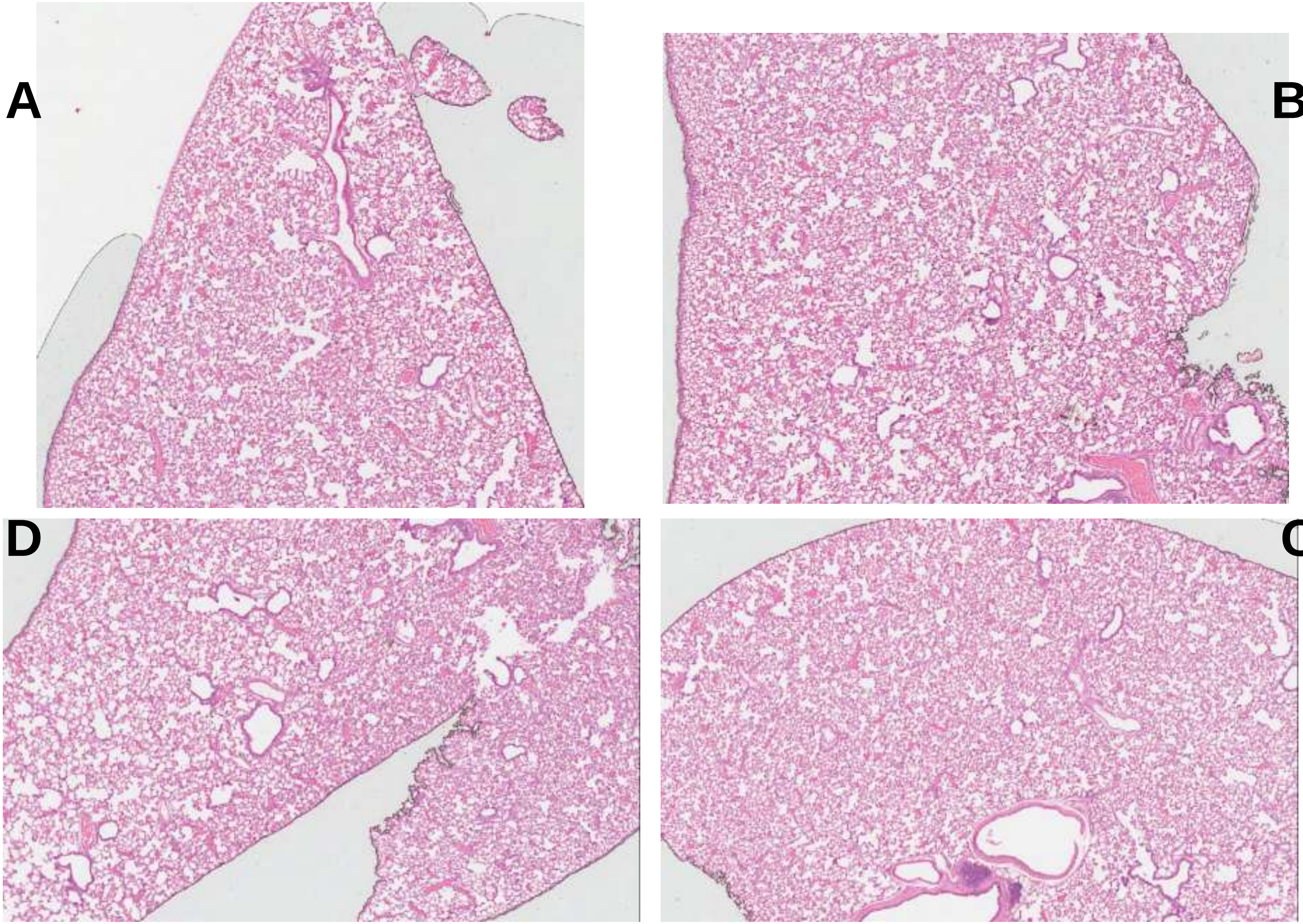

Slide # 18

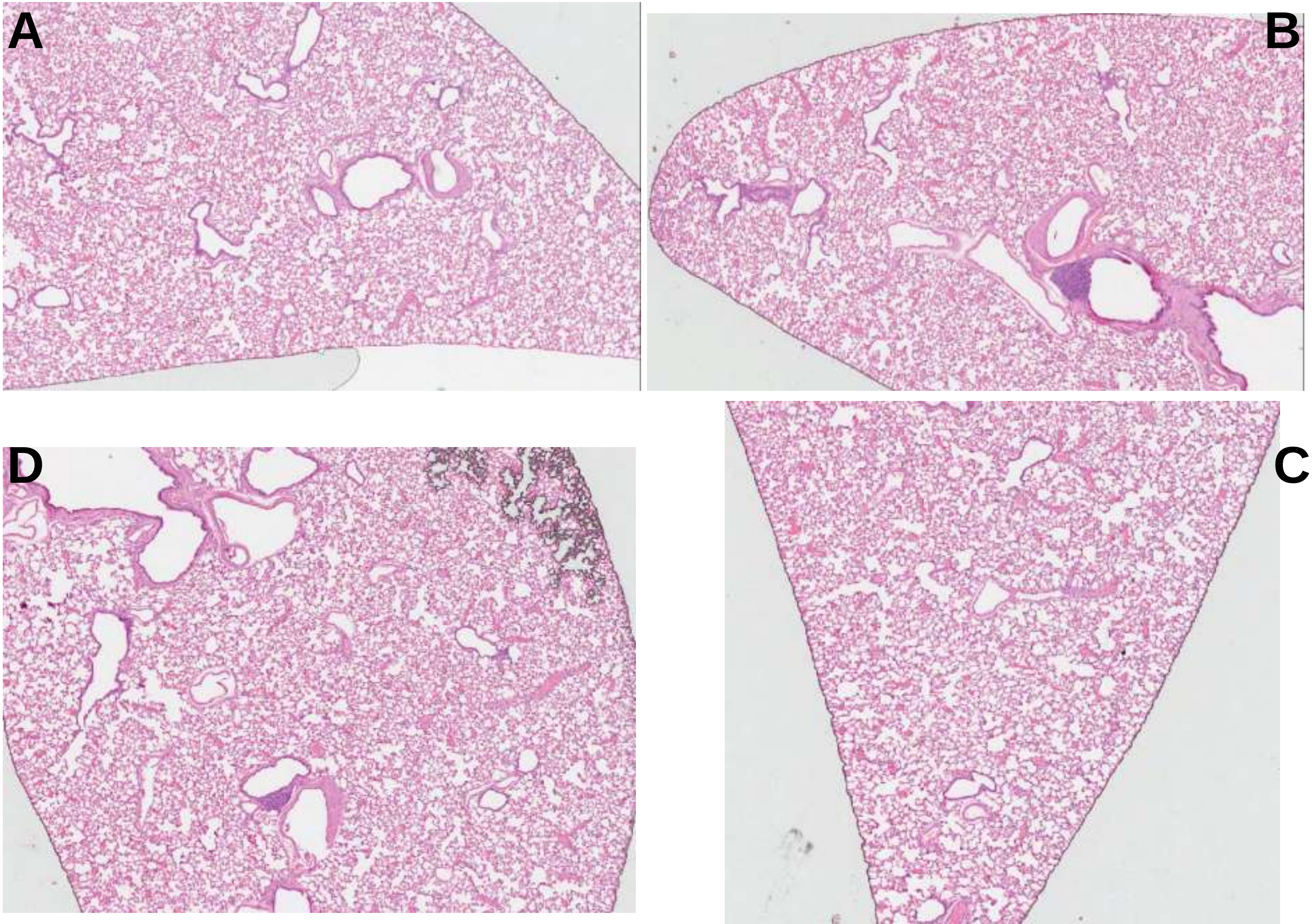

32

Slide # 19

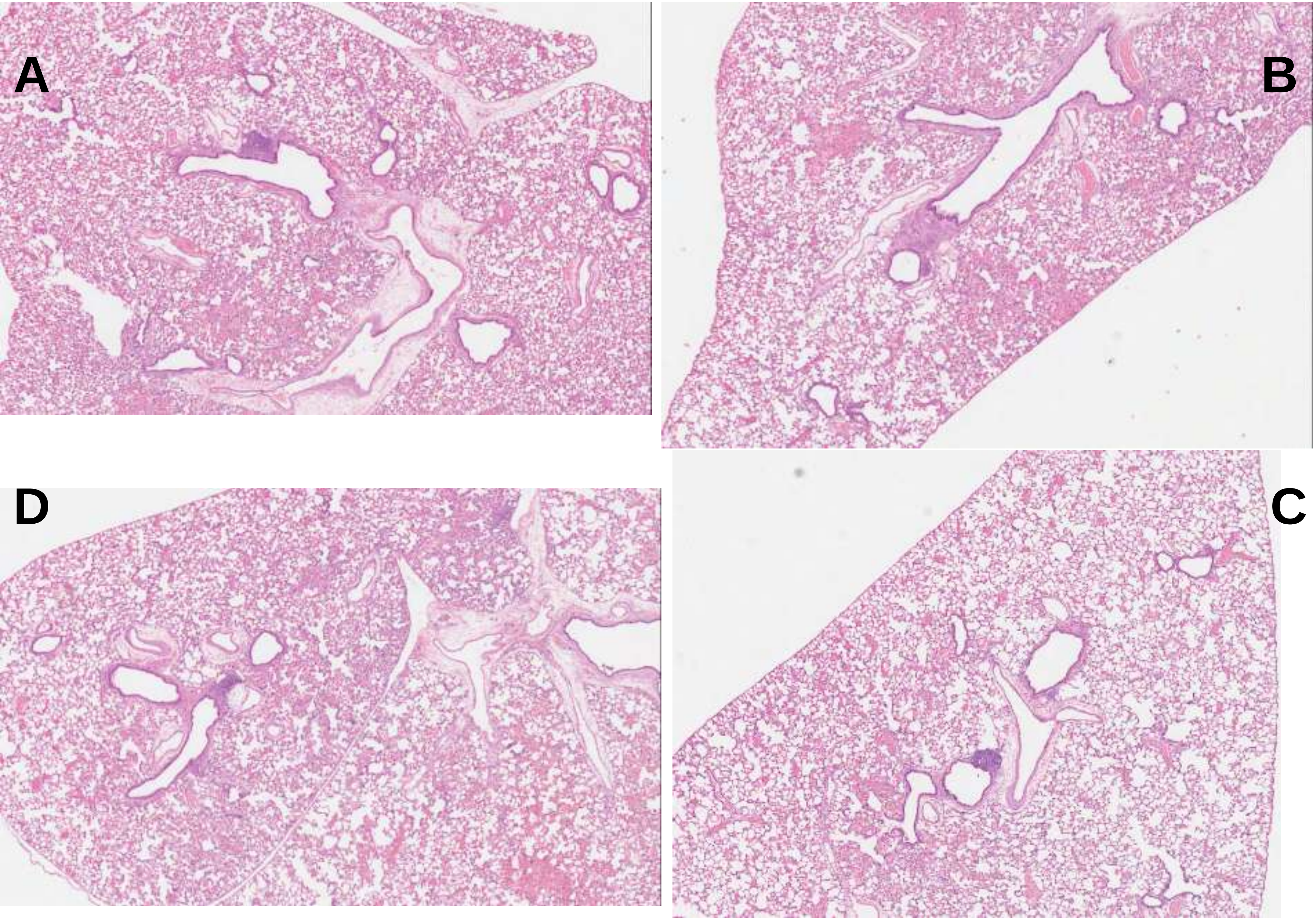

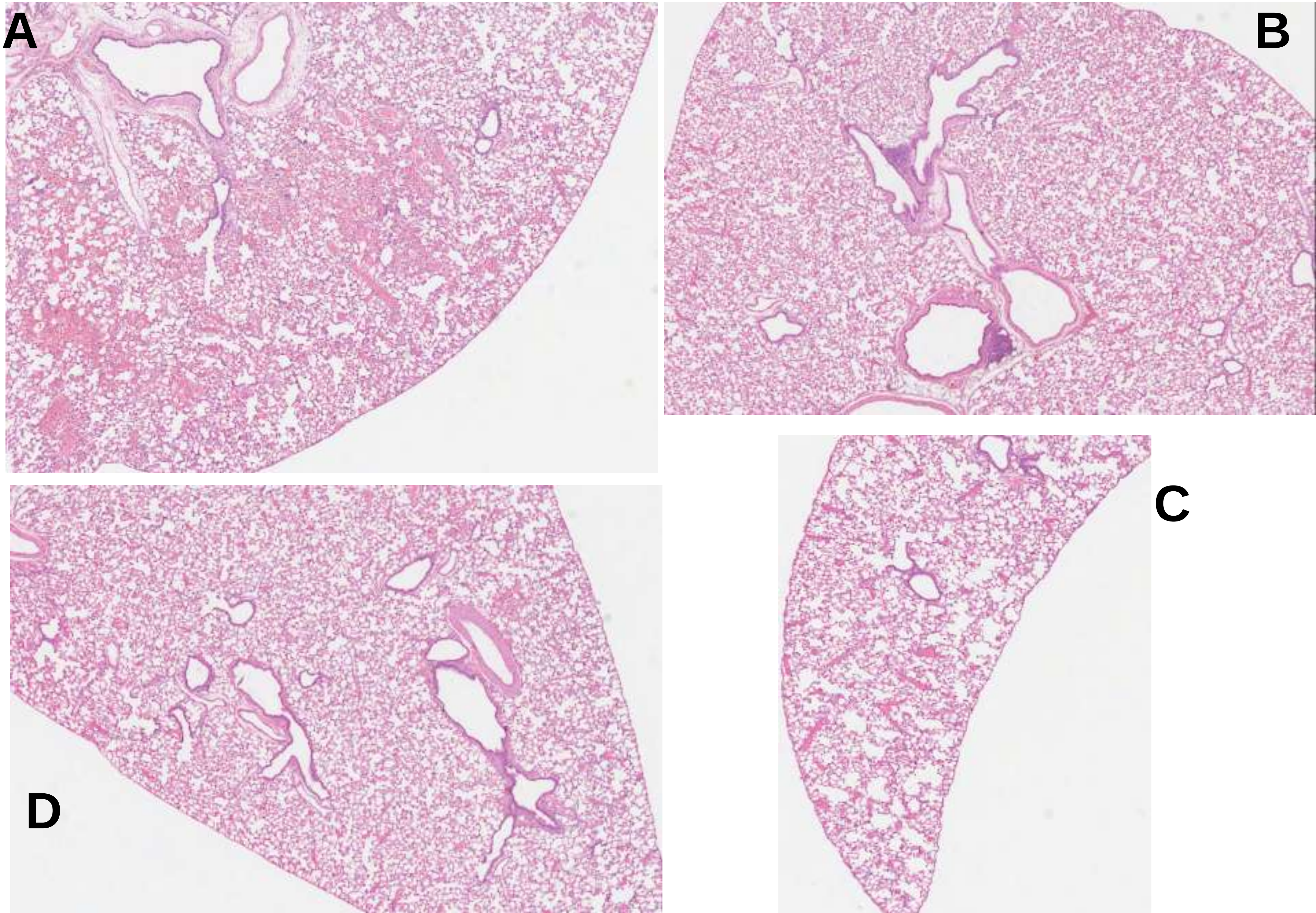
A
B
C
D

Slide # 20

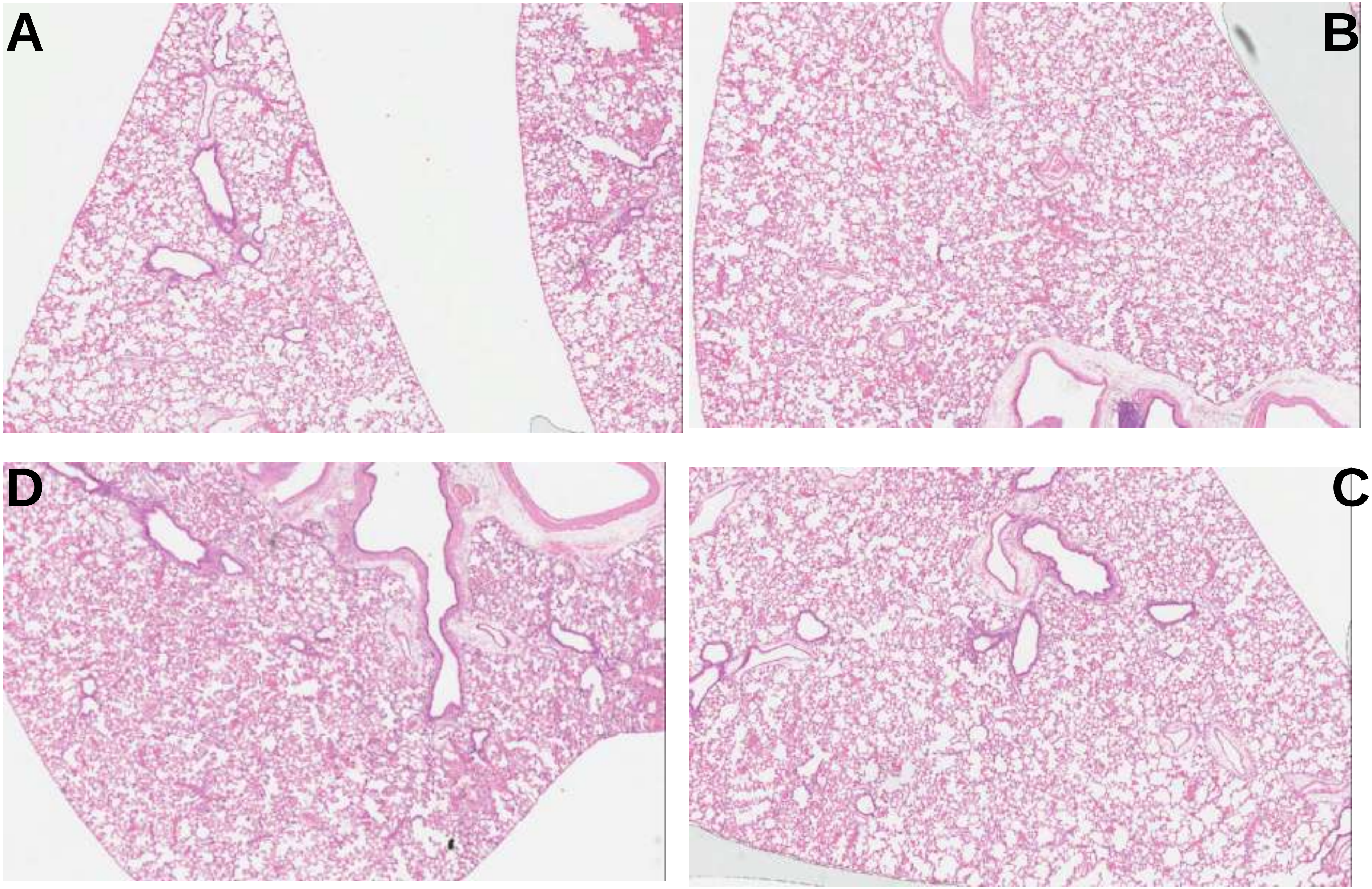

Slide # 20

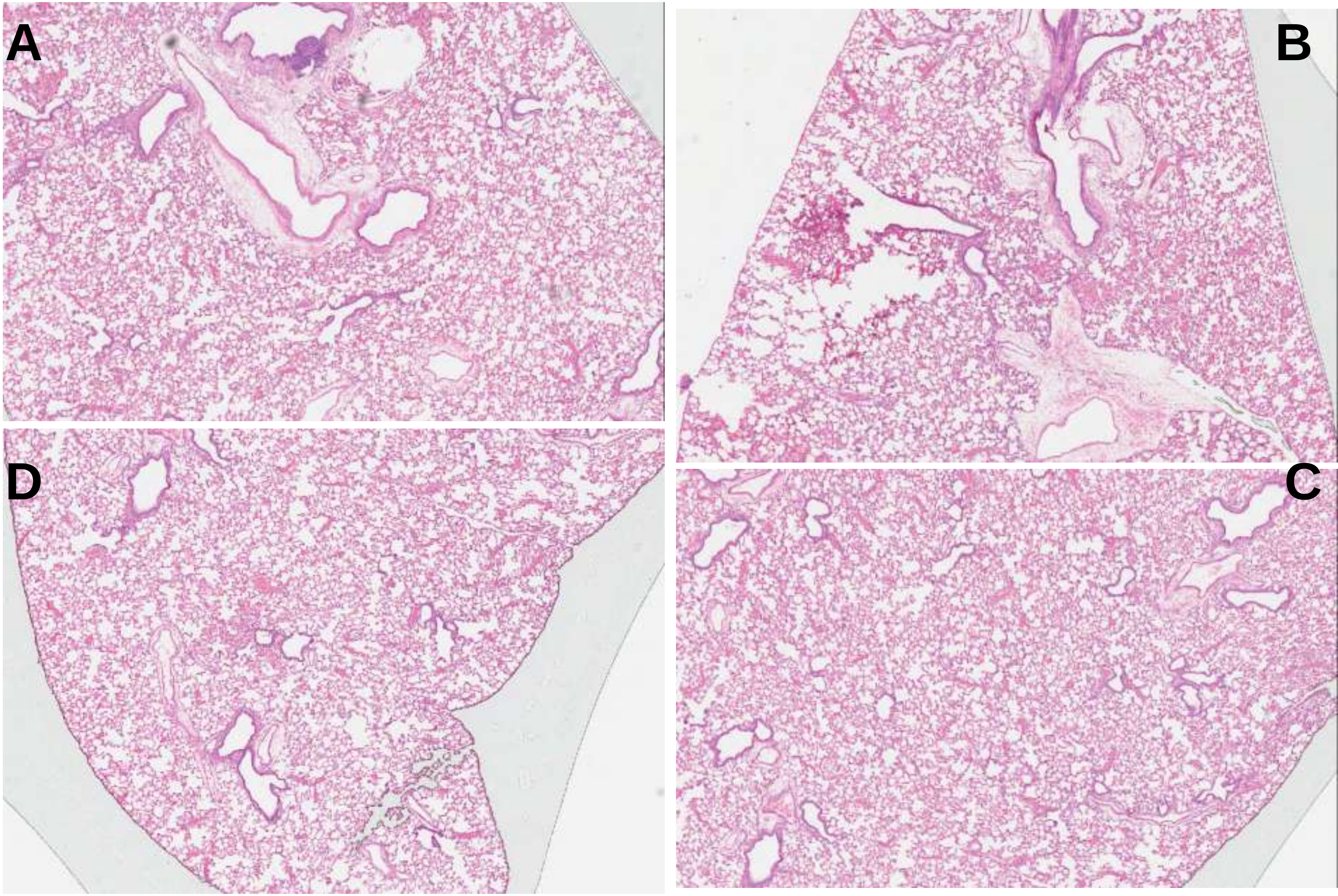

Slide # 20

A

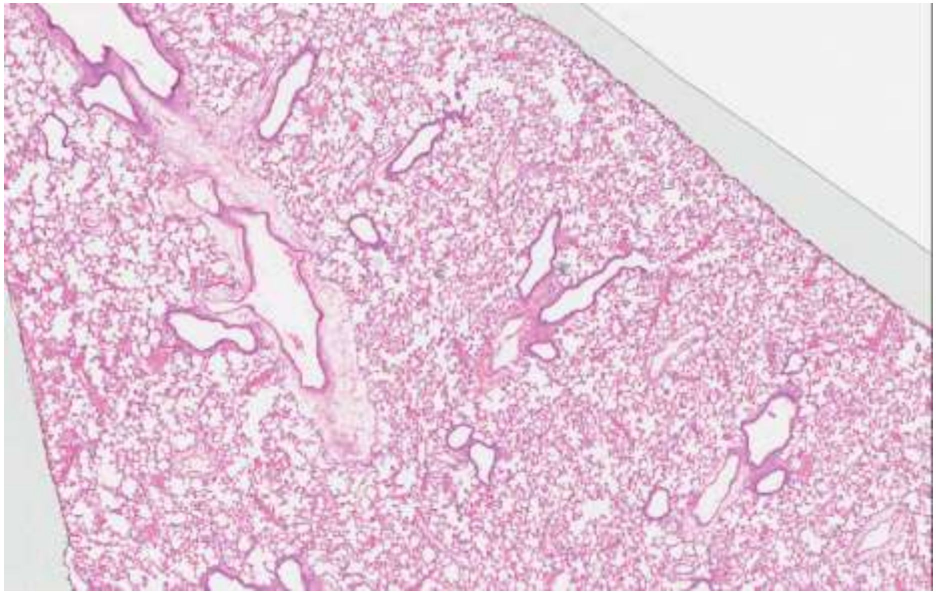

B

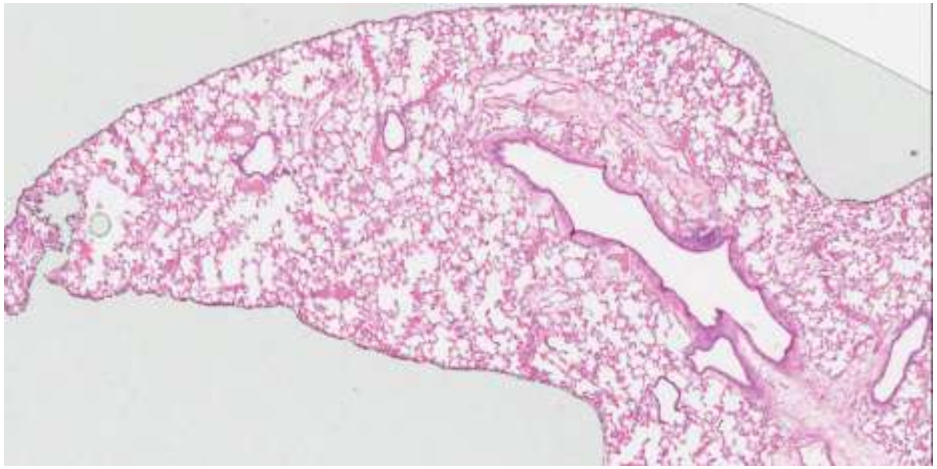

Slide # 21

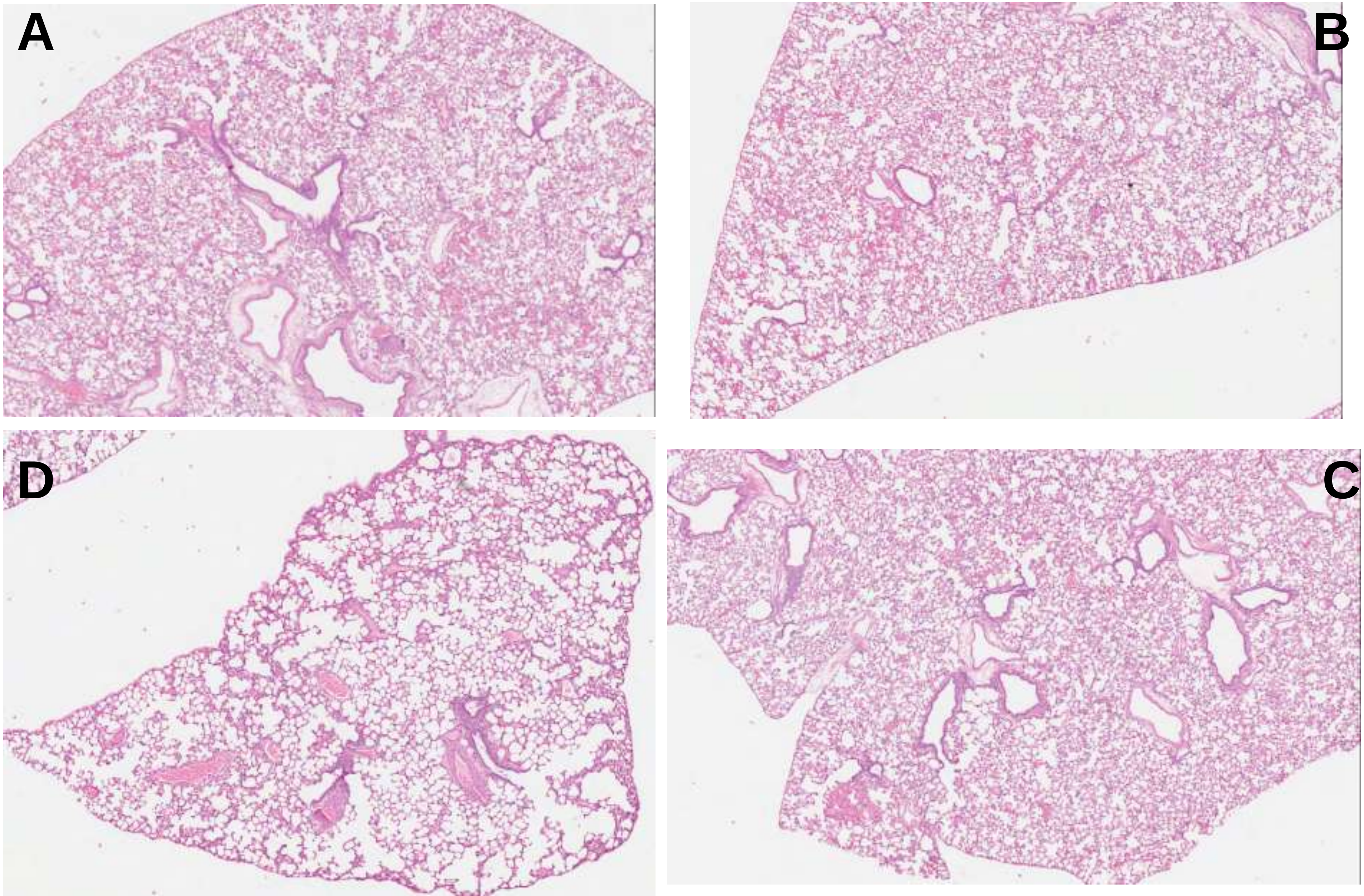

Slide # 21

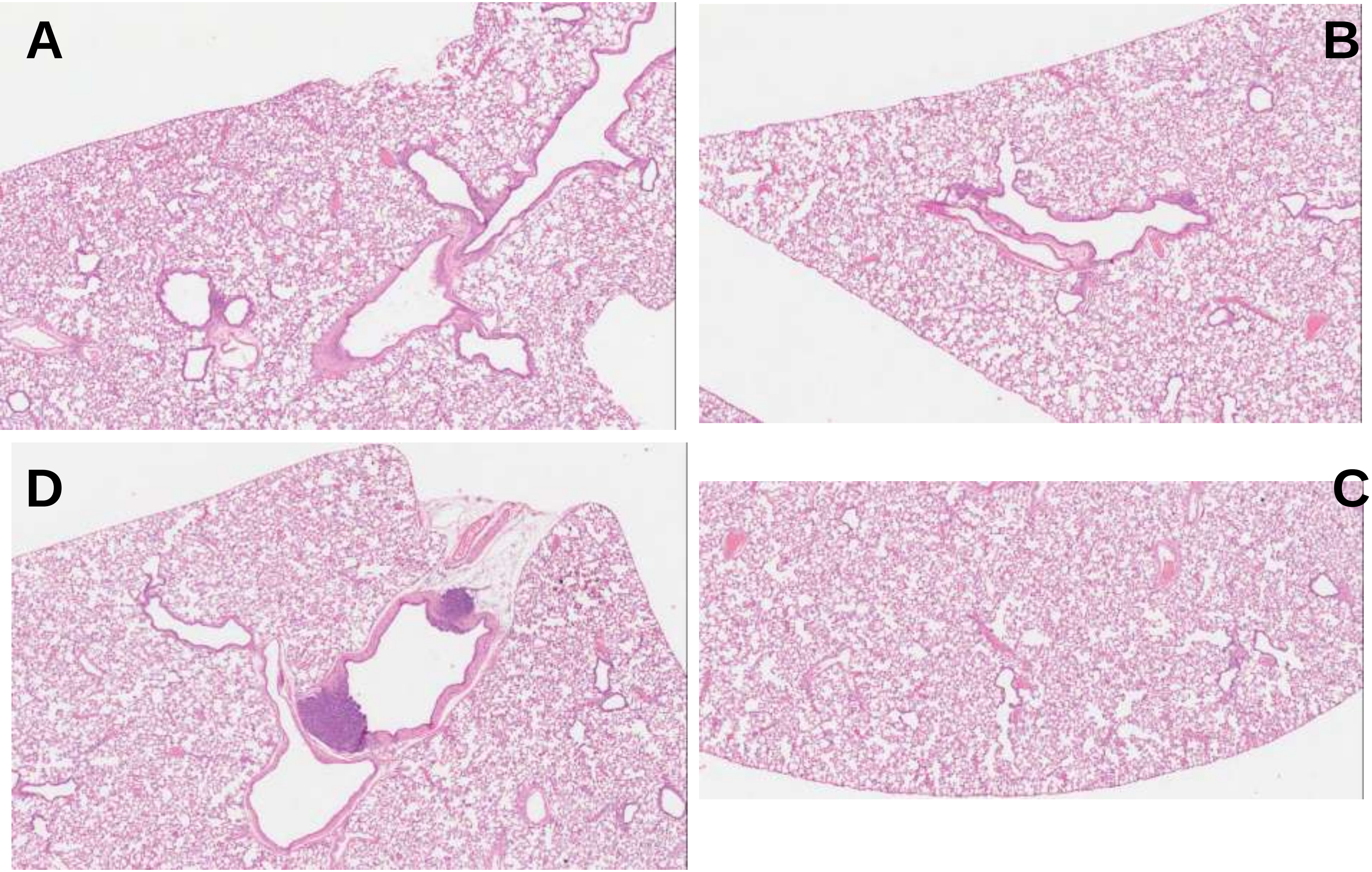

Slide # 22

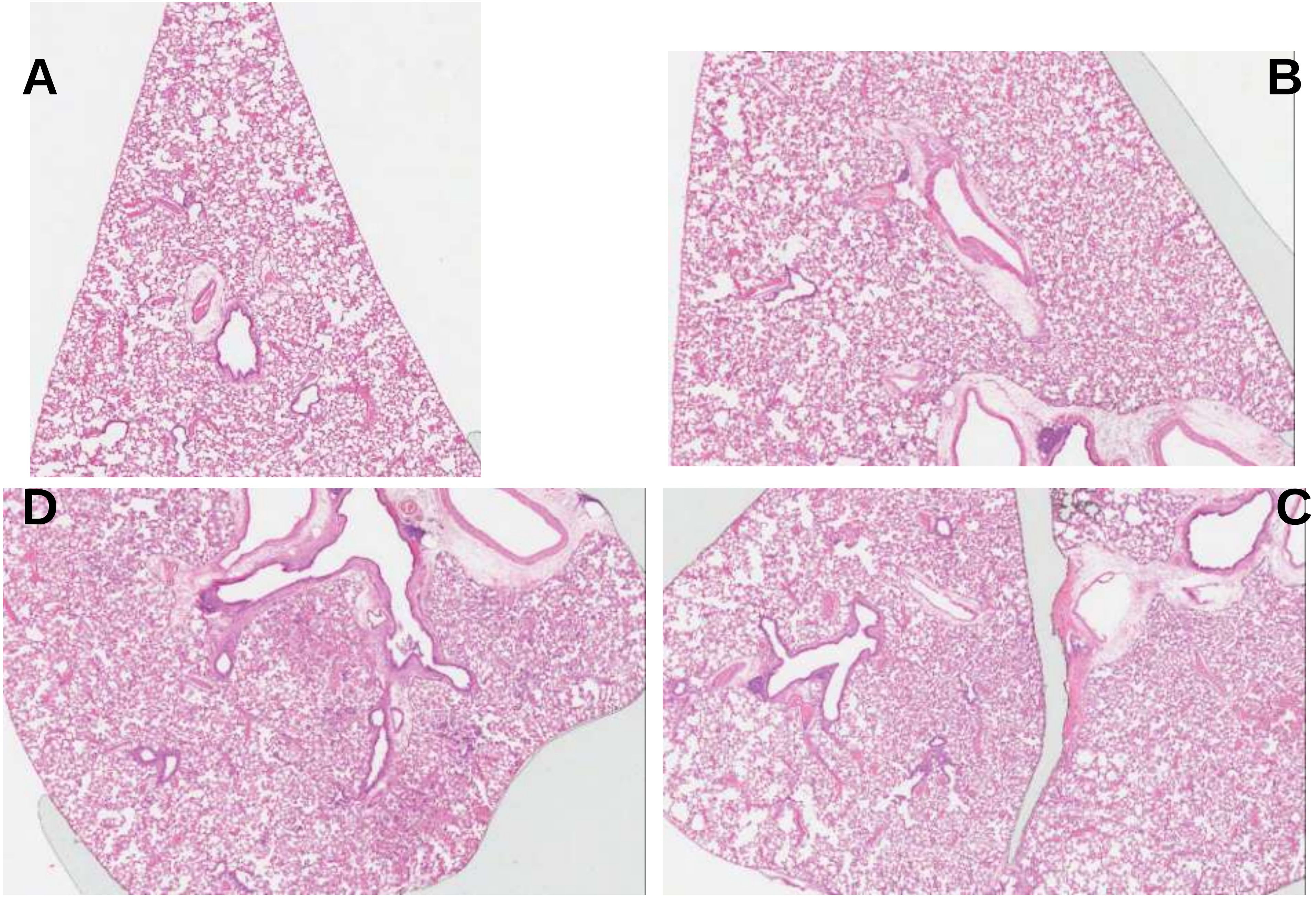

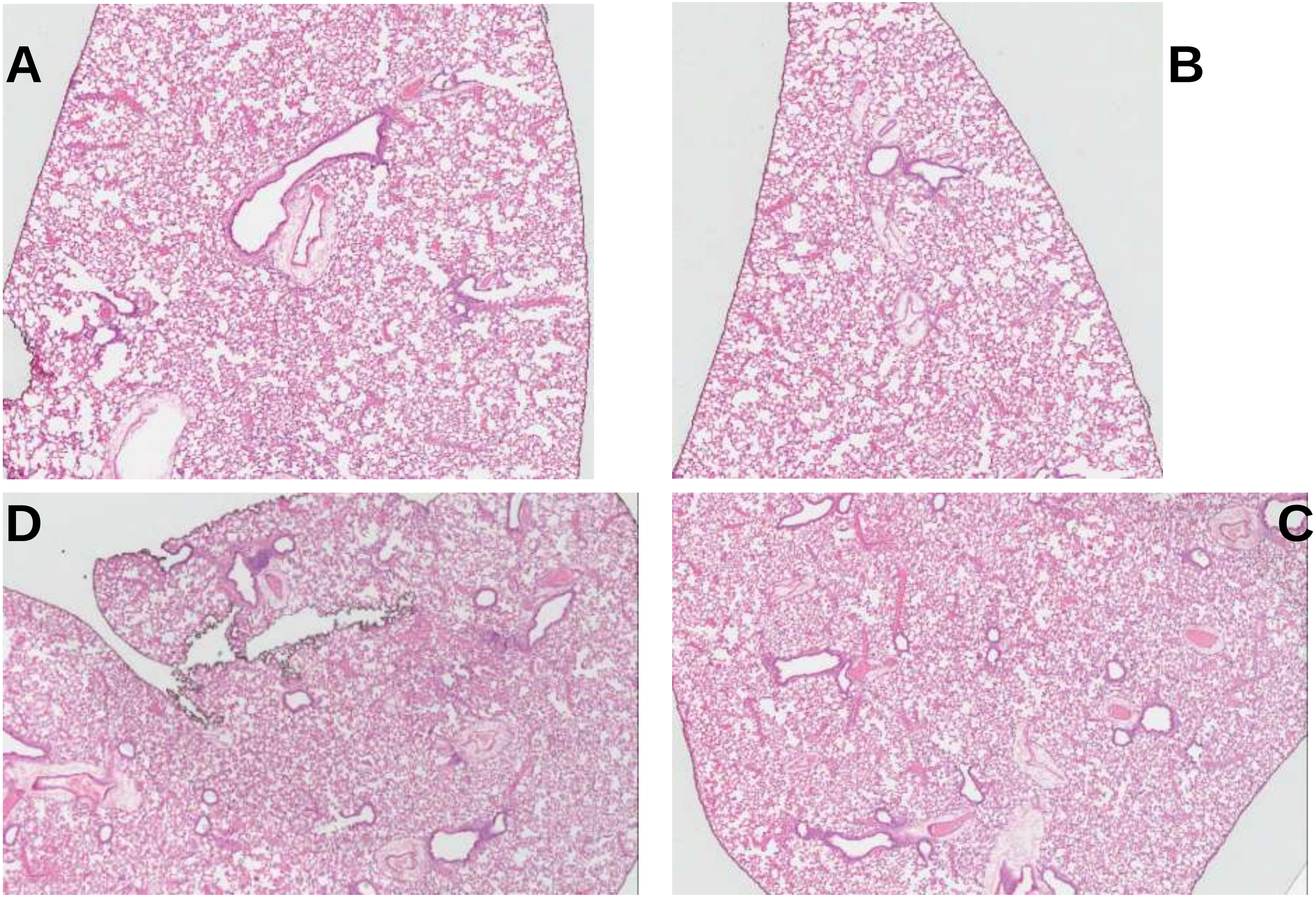
A
B
D
C

Slide # 23

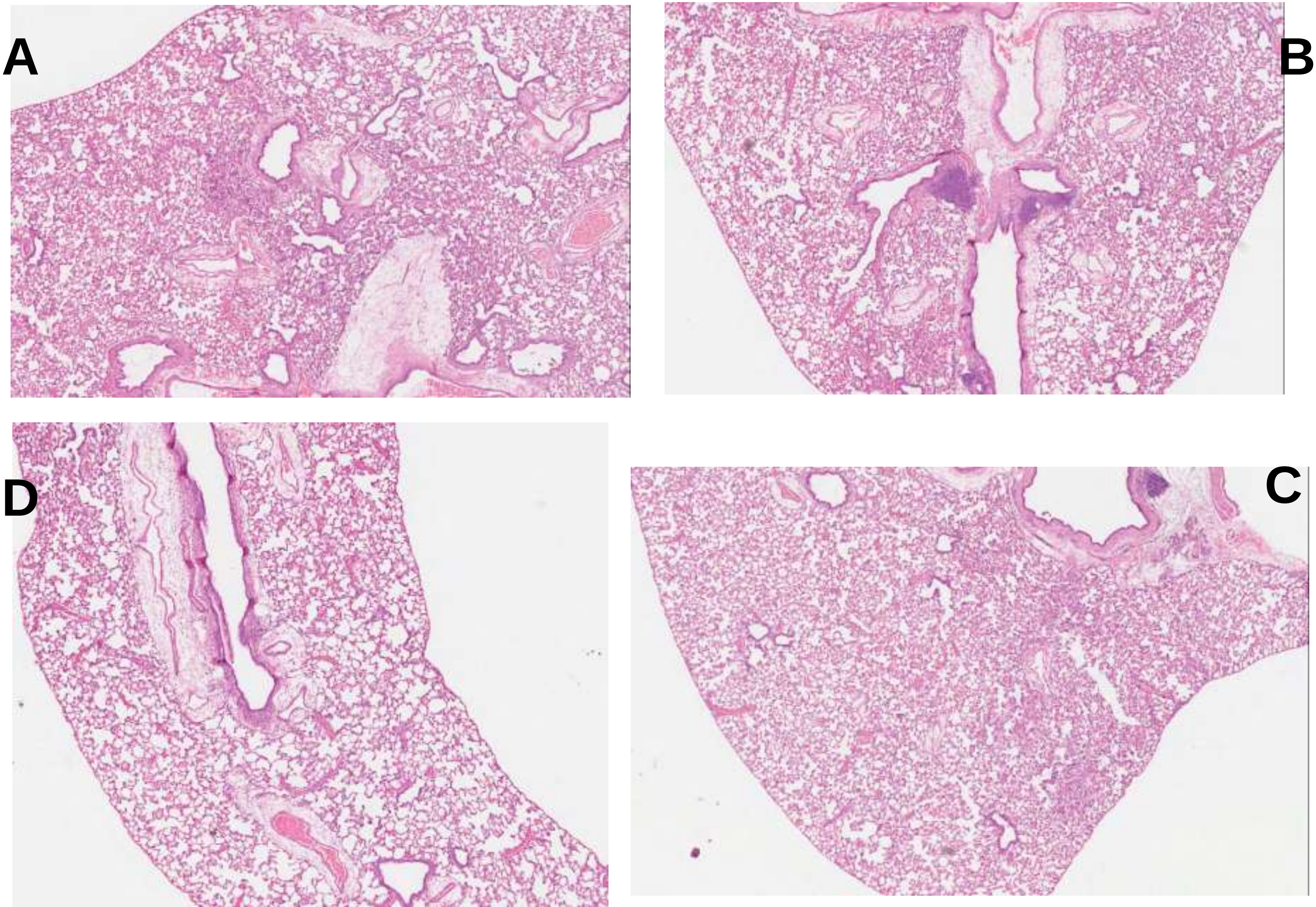

Slide # 23

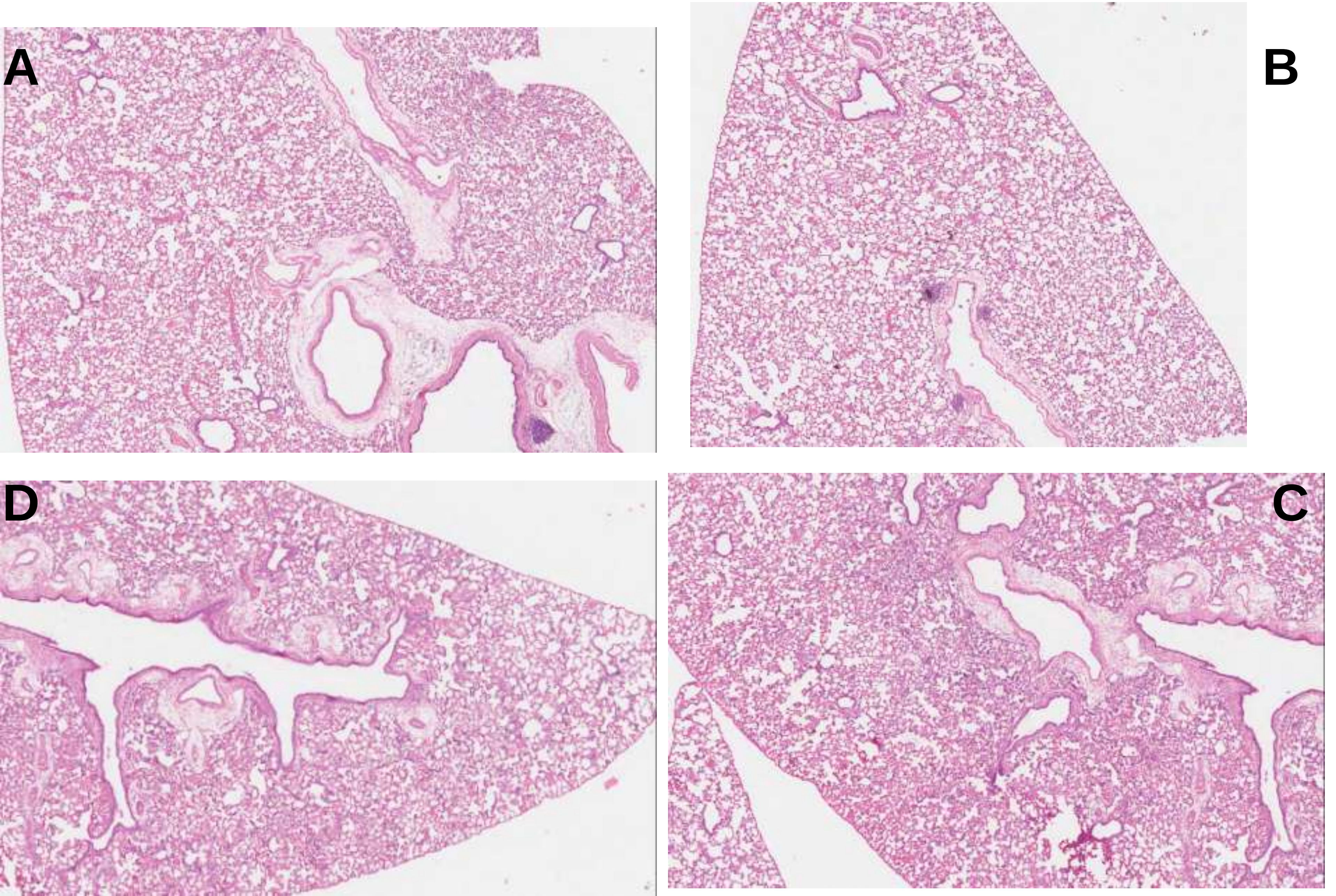

43

Slide # 23

A

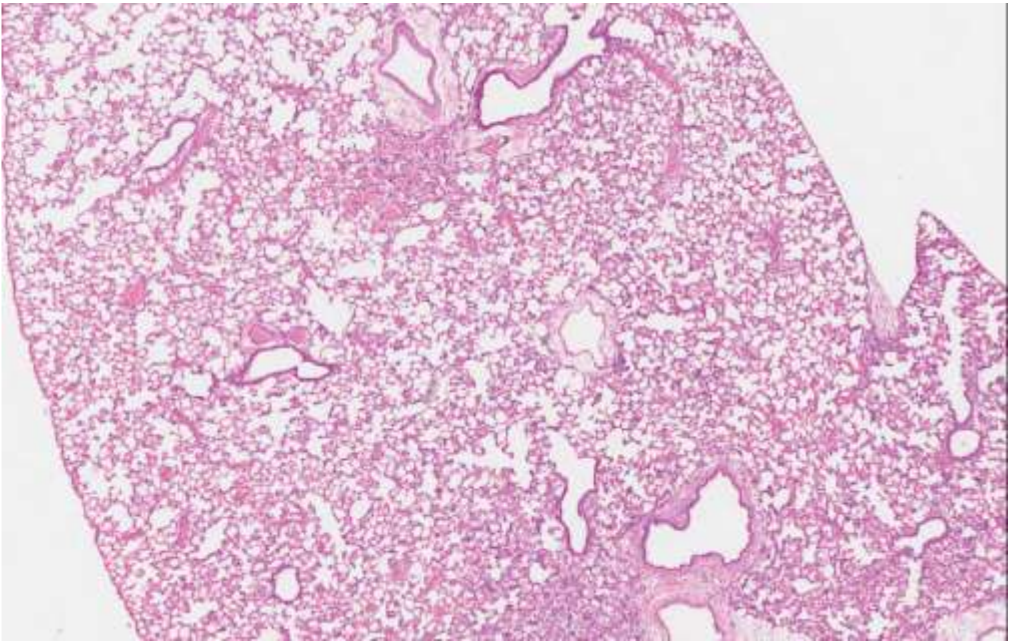

B

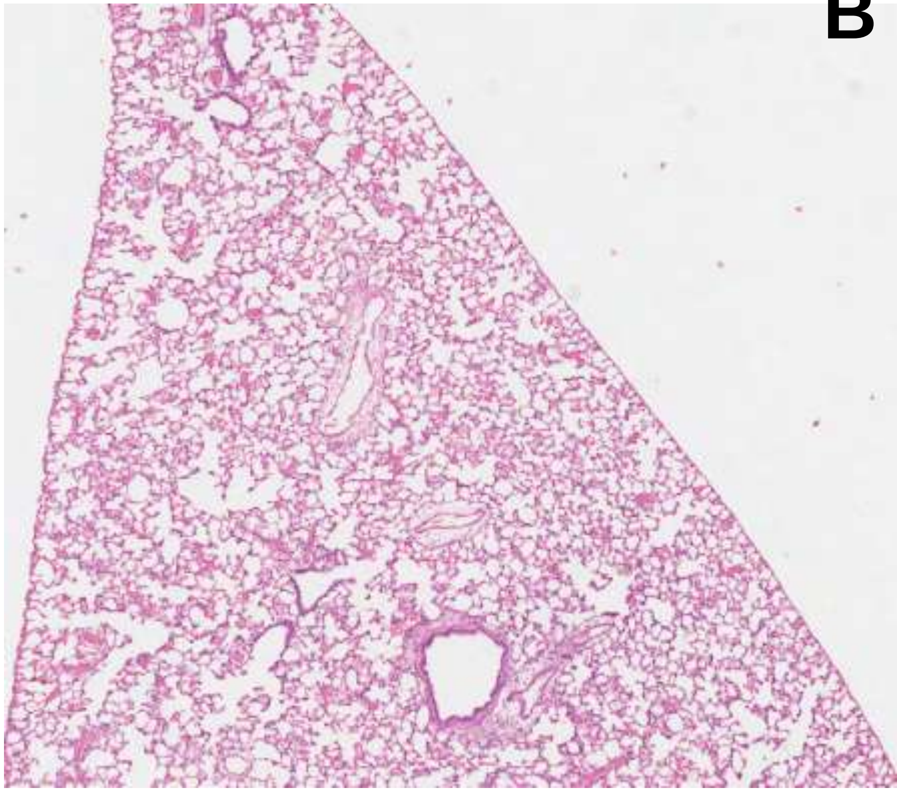

Slide # 24

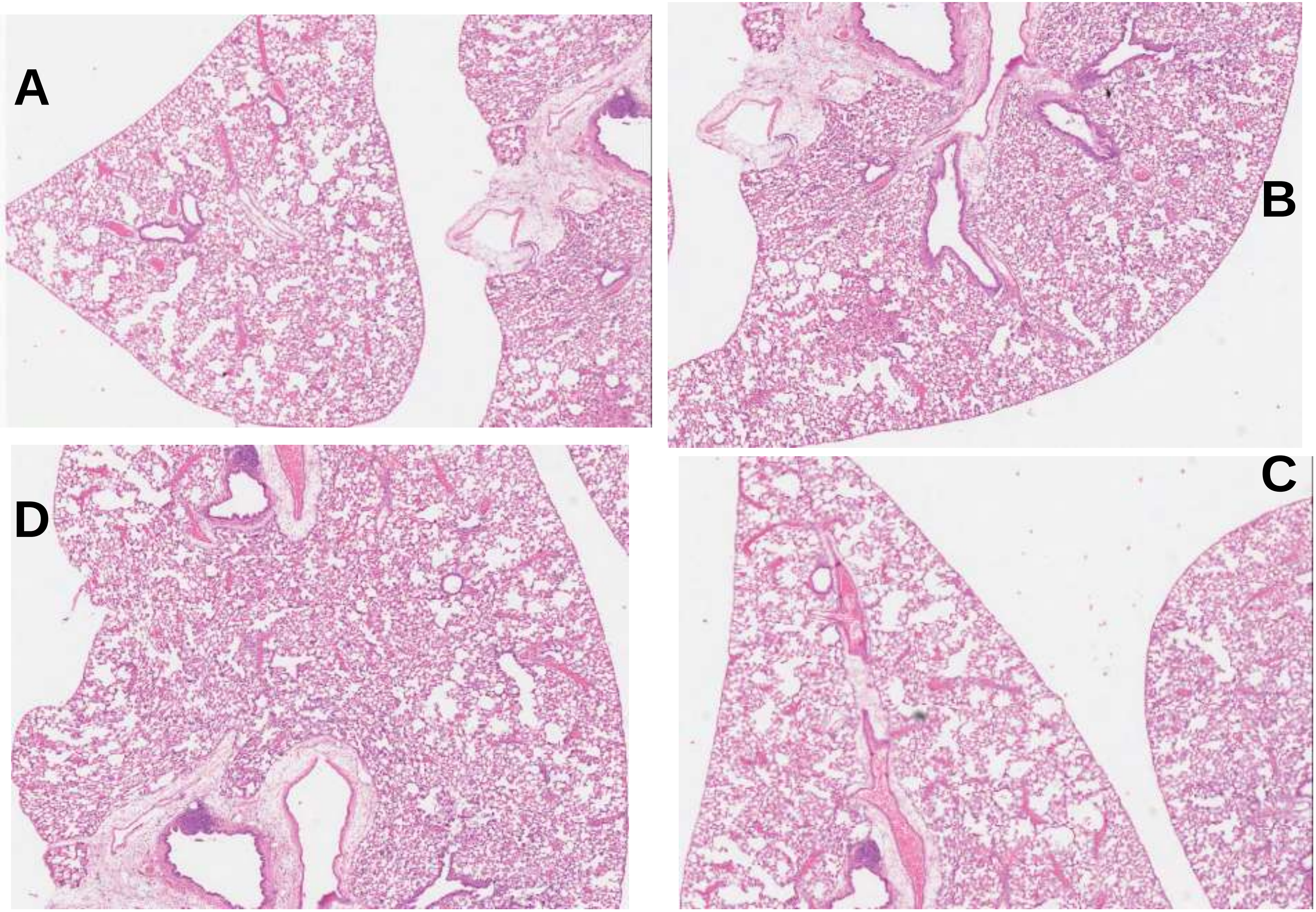

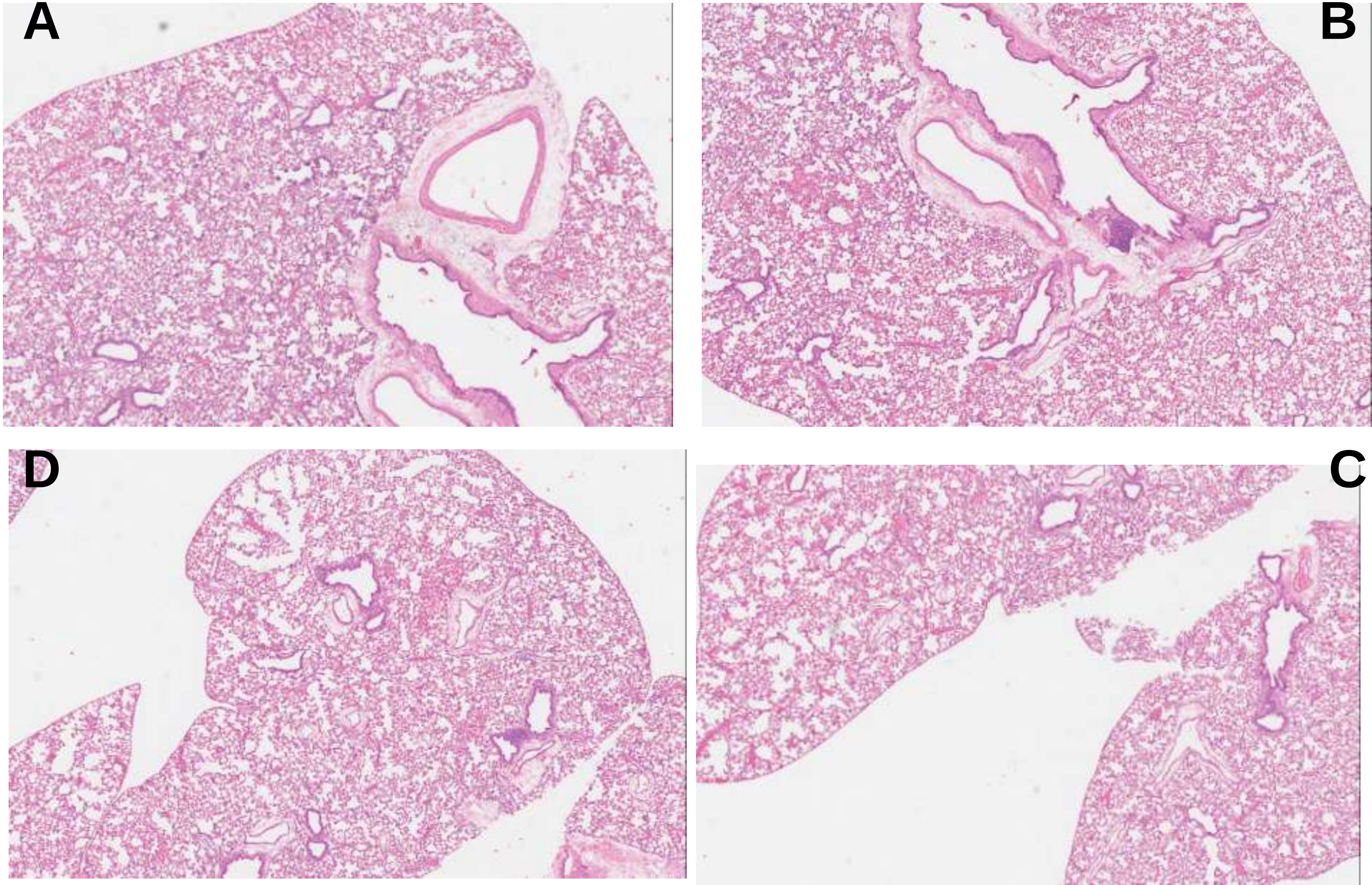
A
B
D
C

Slide # 24

A

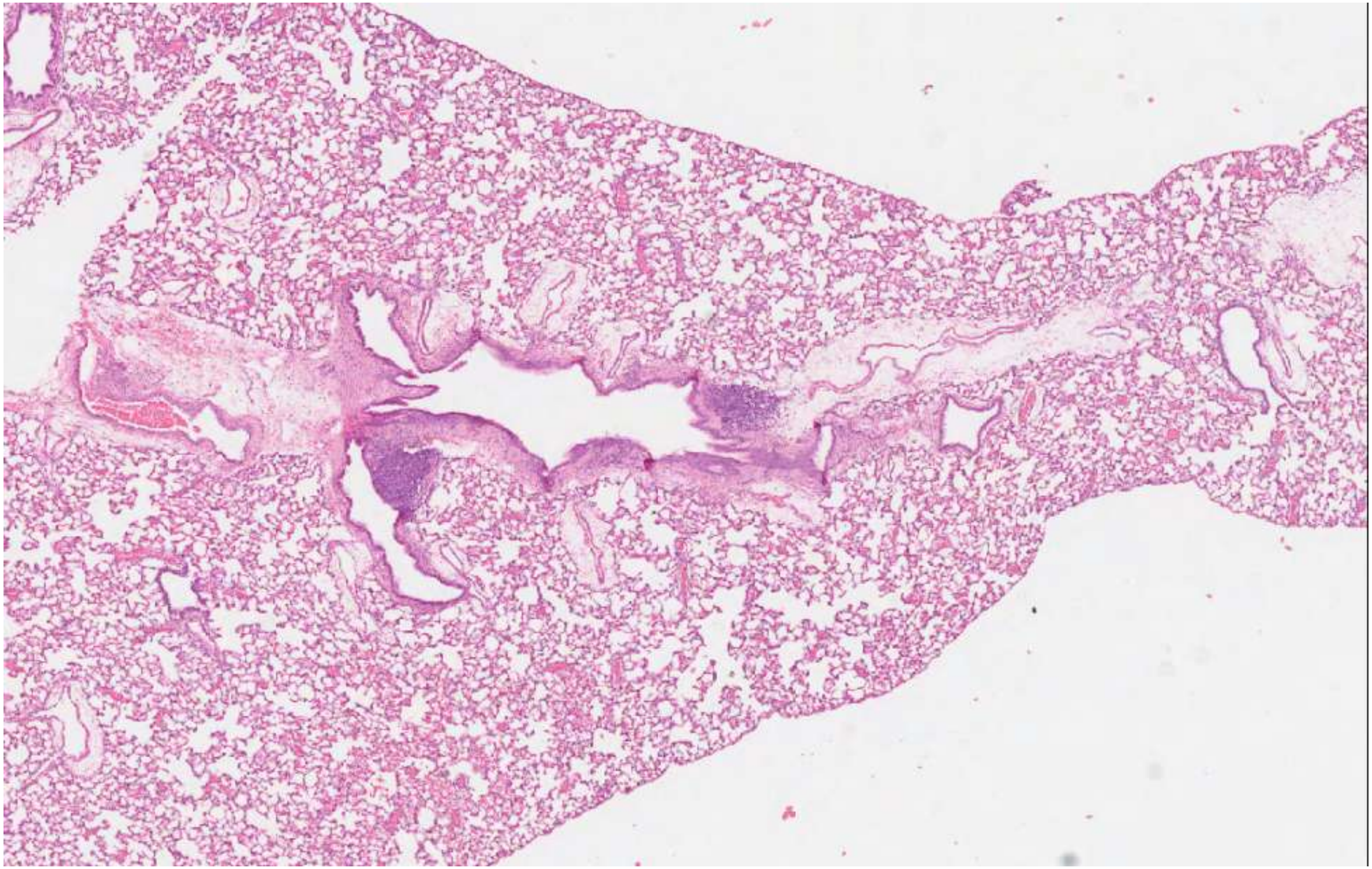

Supplement: S13 Dataset — (PDF) [file pone.0121637.s022.PDF]
