# Supplementary material for: Baclofen, a GABABR Agonist, Ameliorates Immune-Complex Mediated Acute Lung Injury by Modulating Pro-Inflammatory Mediators
Source: PLoS One. 2015 Apr 7;10(4):e0121637. doi: 10.1371/journal.pone.0121637 (PMC4388838; doi:10.1371/journal.pone.0121637)

Human lung tissue sections

Control

Immunohistochemistry: anti-$GABA_BR2$

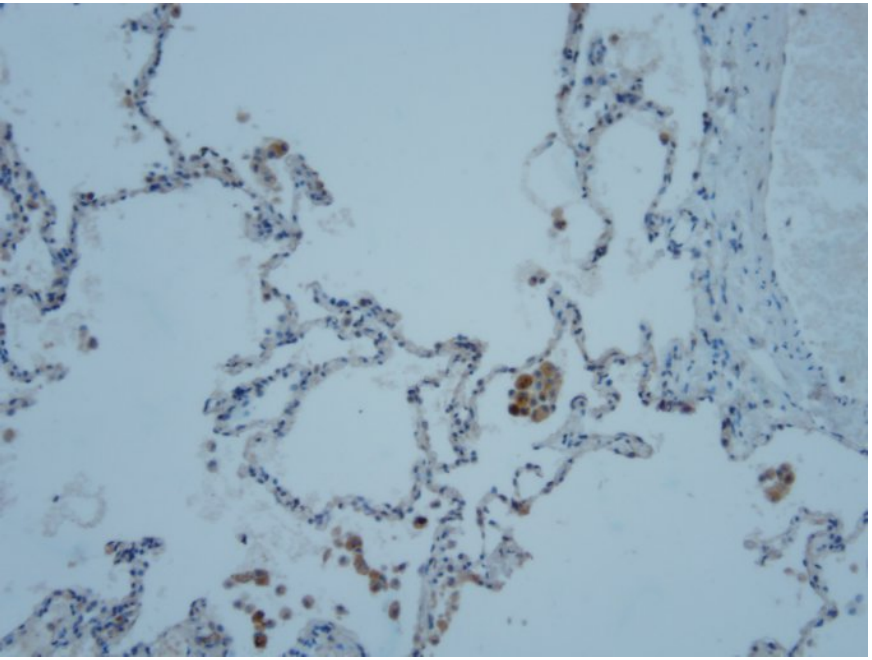

10X

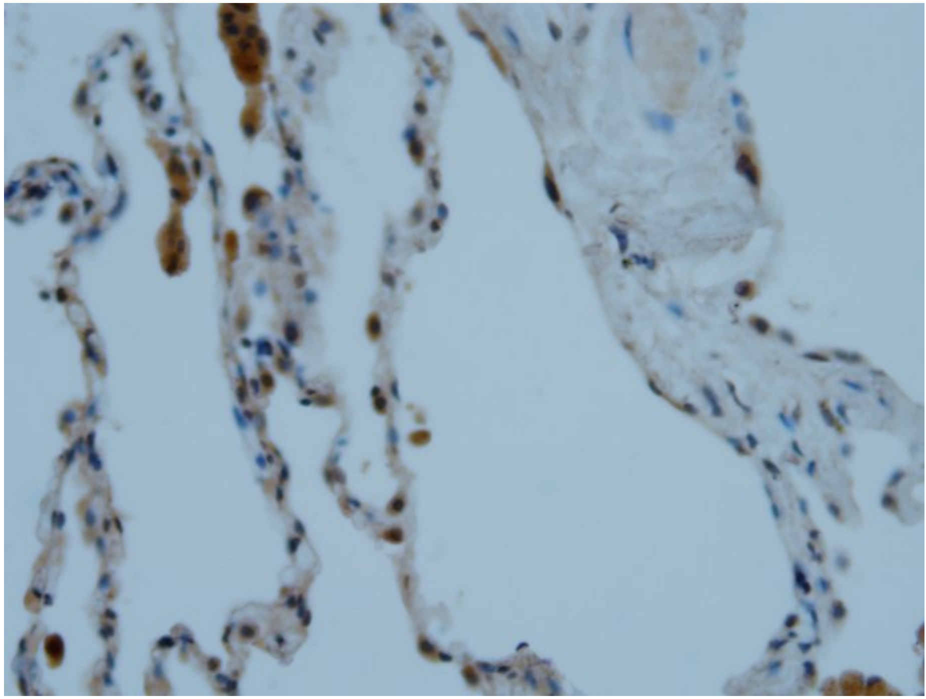

40X

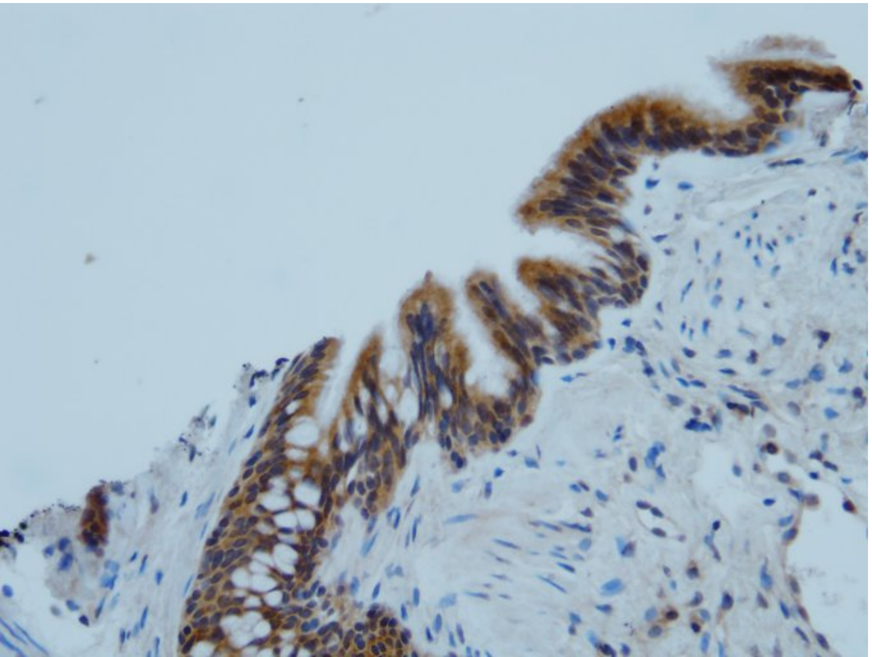

40X

20X Immunohistochemistry: anti-GABA$_B$R2 40X Patient 1

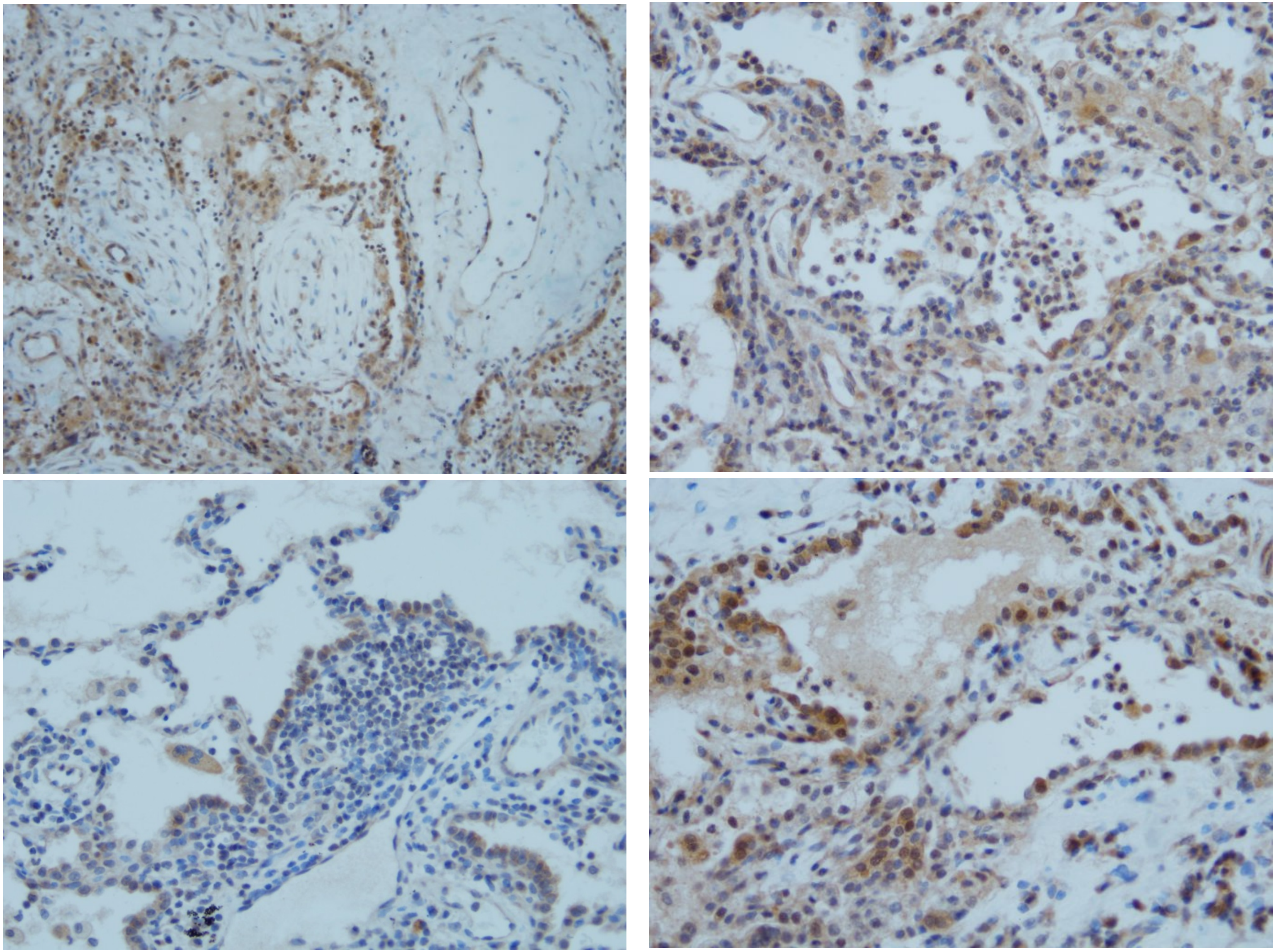

Patient 2

Immunohistochemistry: anti-$GABA_BR2$

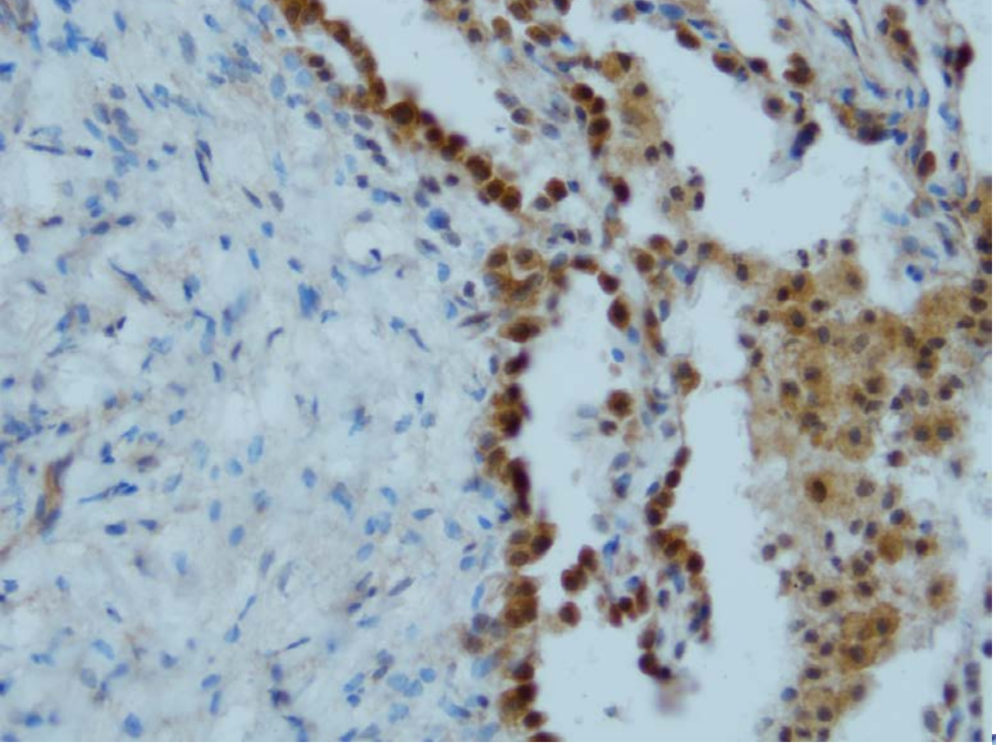

20X

20X

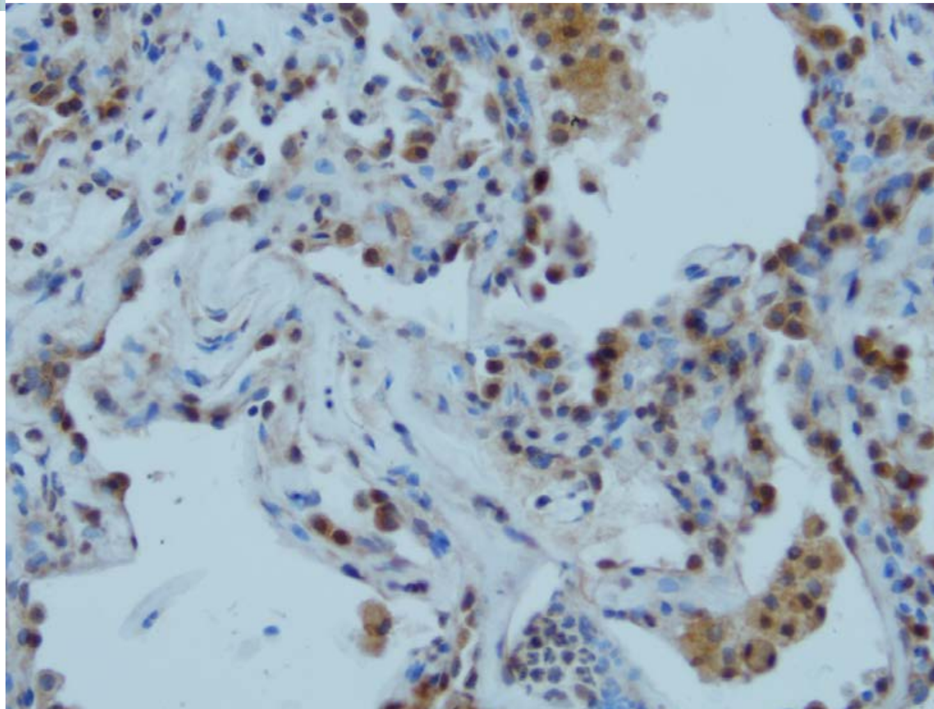

Patient 3

Immunohistochemistry: anti-$GABA_BR2$

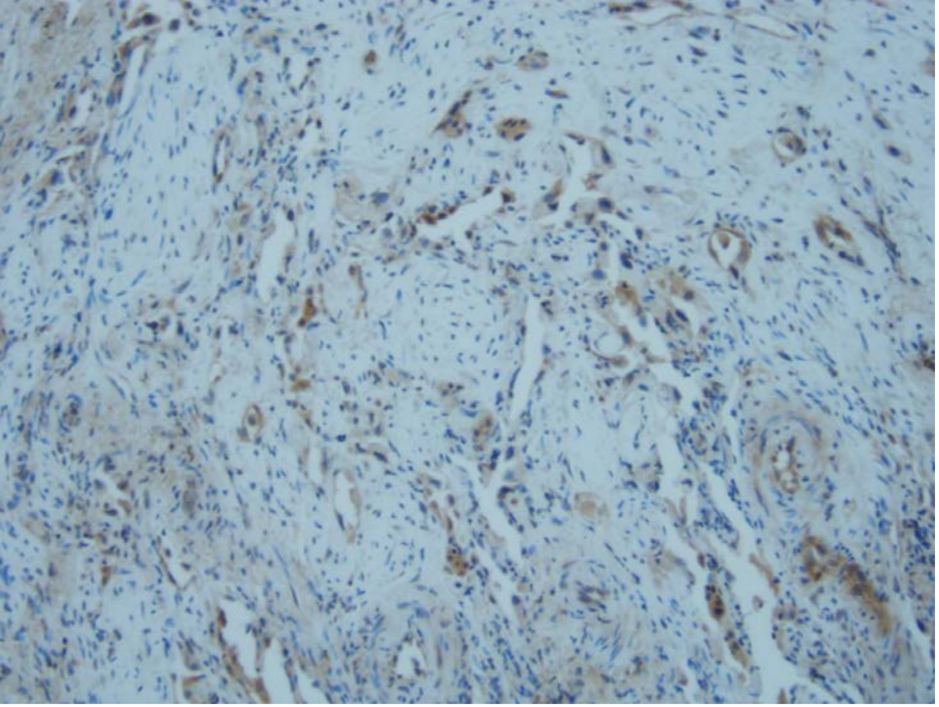

10X

20X

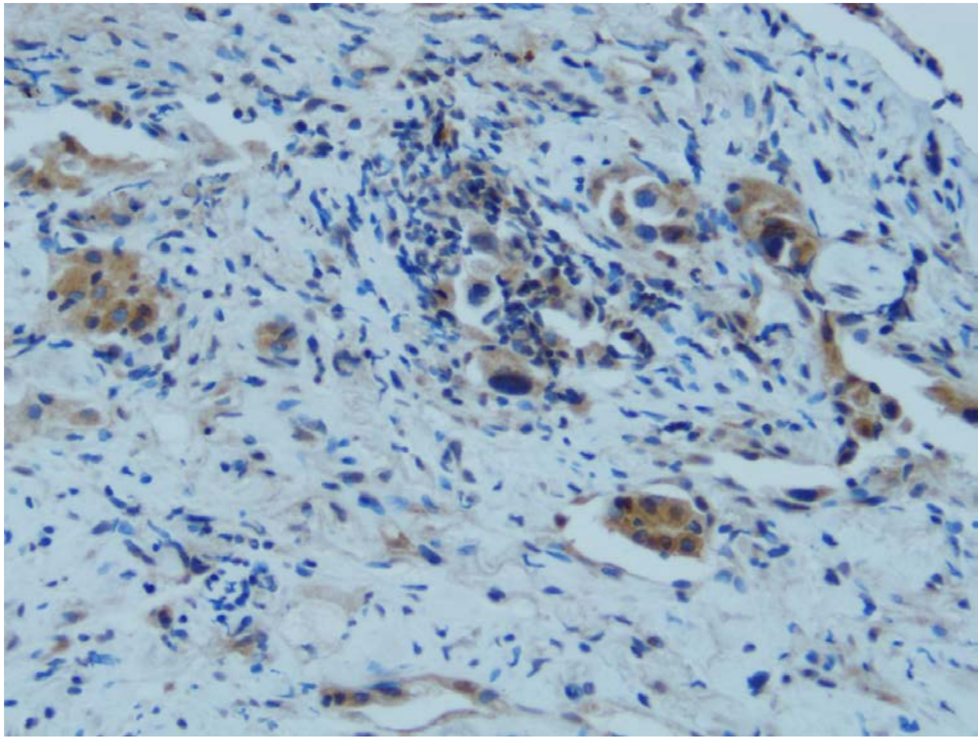

Patient 4

Immunohistochemistry: anti-GABA$_B$R2

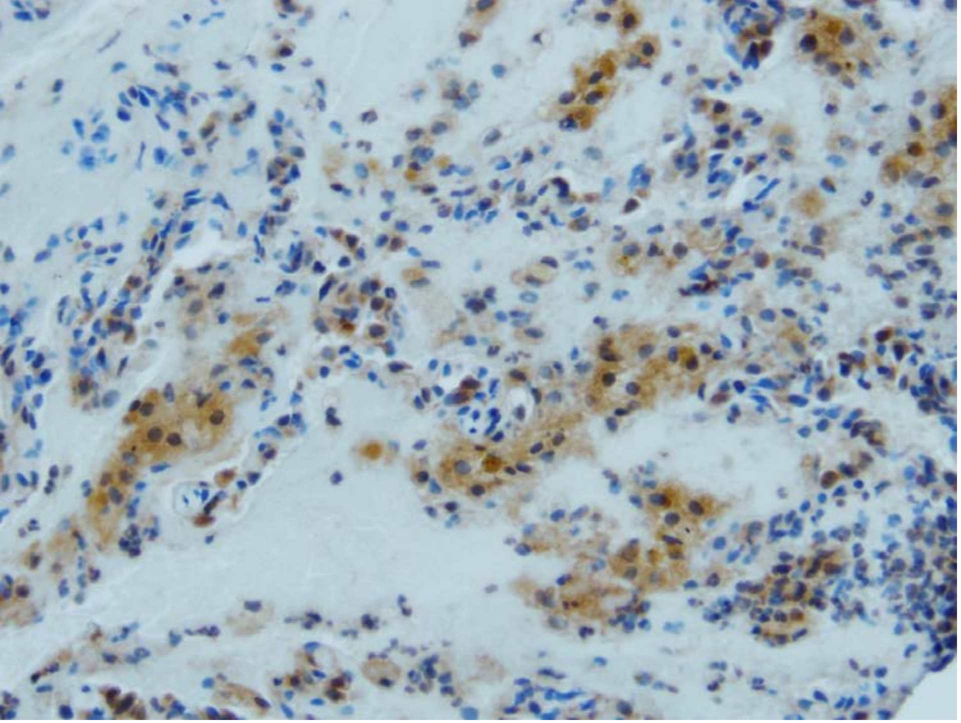

20X

20X

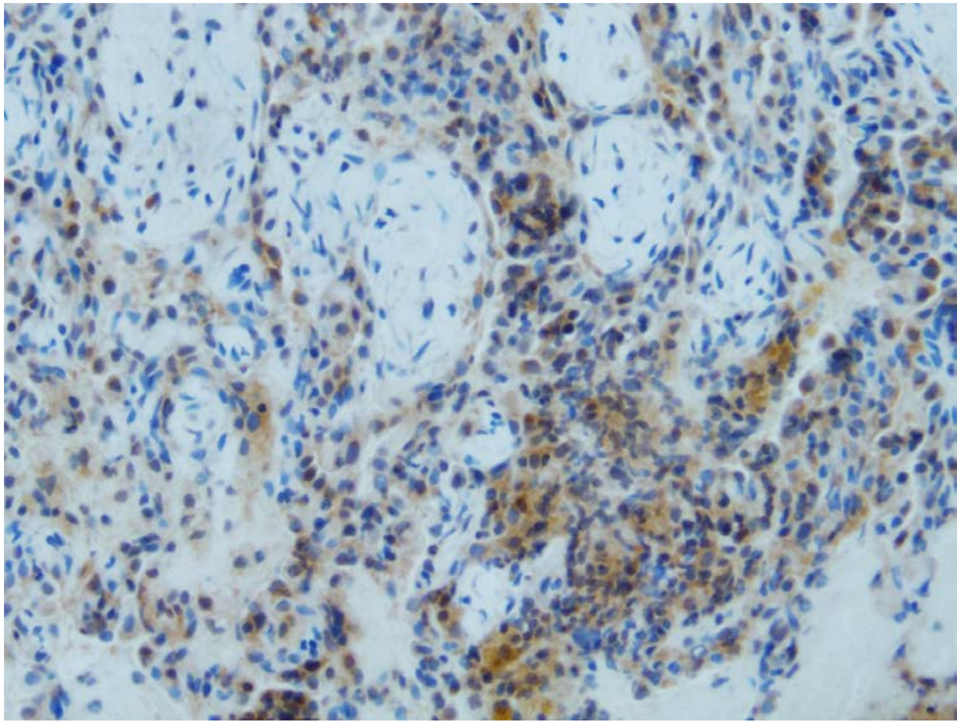

Supplement: S14 Dataset — (PDF) [file pone.0121637.s023.pdf]
